# A population-based resource for intergenerational metabolomics analyses in pregnant women and their children: the Generation R Study

**DOI:** 10.1007/s11306-020-01667-1

**Published:** 2020-03-23

**Authors:** Ellis Voerman, Vincent W. V. Jaddoe, Olaf Uhl, Engy Shokry, Jeannie Horak, Janine F. Felix, Berthold Koletzko, Romy Gaillard

**Affiliations:** 1 The Generation R Study Group, Erasmus MC, University Medical Center, Rotterdam, The Netherlands; 2 Department of Pediatrics, Erasmus MC, University Medical Center, Rotterdam, The Netherlands; 3 Division of Metabolic and Nutritional Medicine, Dr. Von Hauner Children’s Hospital, LMU - Ludwig-Maximilians Universität München, Munich, Germany; 4 The Generation R Study Group, Erasmus MC, University Medical Center, Room Na-2908, PO Box 2040, 3000 CA Rotterdam, The Netherlands

**Keywords:** Metabolomics, Amino acids, Fatty acids, Phospholipids, Carnitines, Birth cohort

## Abstract

**Introduction:**

Adverse exposures in early life may predispose children to cardio-metabolic disease in later life. Metabolomics may serve as a valuable tool to disentangle the metabolic adaptations and mechanisms that potentially underlie these associations.

**Objectives:**

To describe the acquisition, processing and structure of the metabolomics data available in a population-based prospective cohort from early pregnancy onwards and to examine the relationships between metabolite profiles of pregnant women and their children at birth and in childhood.

**Methods:**

In a subset of 994 mothers-child pairs from a prospective population-based cohort study among pregnant women and their children from Rotterdam, the Netherlands, we used LC–MS/MS to determine concentrations of amino acids, non-esterified fatty acids, phospholipids and carnitines in blood serum collected in early pregnancy, at birth (cord blood), and at child’s age 10 years.

**Results:**

Concentrations of diacyl-phosphatidylcholines, acyl-alkyl-phosphatidylcholines, alkyl-lysophosphatidylcholines and sphingomyelines were the highest in early pregnancy, concentrations of amino acids and non-esterified fatty acids were the highest at birth and concentrations of alkyl-lysophosphatidylcholines, free carnitine and acyl-carnitines were the highest at age 10 years. Correlations of individual metabolites between pregnant women and their children at birth and at the age of 10 years were low (range between *r* = − 0.10 and *r* = 0.35).

**Conclusion:**

Our results suggest that unique metabolic profiles are present among pregnant women, newborns and school aged children, with limited intergenerational correlations between metabolite profiles. These data will form a valuable resource to address the early metabolic origins of cardio-metabolic disease.

**Electronic supplementary material:**

The online version of this article (10.1007/s11306-020-01667-1) contains supplementary material, which is available to authorized users.

## Introduction

Cardio-metabolic diseases are of major public health concern (NCD Risk Factor Collaboration 2016, 2017a, b). The pathogenesis of these cardio-metabolic diseases involves adaptations in metabolic pathways. Thus far, studies mainly focused on a small set of conventional biomarkers to assess metabolic status and pathways. Recent developments in high-throughput technologies and analytical methods have enabled the application of metabolomics for detailed characterization of an individual’s metabolic status on a large scale (Bictash et al. 2010; Tzoulaki et al. 2014; van Roekel et al. 2019). Metabolomics measures a large number of low molecular weight metabolites in biological tissues and fluids. The metabolome is the most downstream component of biological processes and closely linked to the phenotype. It carries information about gene expression as well as lifestyle- and environmental factors (Tzoulaki et al. 2014; van Roekel et al. 2019). Metabolomics has already been successfully applied in large-scale epidemiological studies, mainly in adult populations, to identify new biomarkers of cardio-metabolic disease status, development and progression, as well as the underlying pathophysiological mechanisms (Newgard 2017; Rangel-Huerta et al. 2019; Ussher et al. 2016).

Accumulating evidence suggests that cardio-metabolic diseases might originate in early life. Adverse exposures in early life may lead to developmental adaptations in organ structure or function, which may predispose these children to later cardio-metabolic disease (Gluckman et al. 2008). Early-life developmental adaptations in metabolic pathways may underlie these associations. Only a limited amount of metabolomics studies on the early origins of cardio-metabolic disease have been performed. Most of these studies were small and mainly assessed cross-sectional relationships (Hivert et al. 2015; Rauschert et al. 2017a). Also, it is unclear whether metabolite profiles correlate between mothers and their children. The application of metabolomics in longitudinal birth cohort studies may serve as a valuable tool to identify biomarkers of metabolic status, in order to disentangle the mechanisms linking adverse exposures in early life to cardio-metabolic disease later in life (Hivert et al. 2015).

Therefore, in a population-based cohort from early pregnancy onwards among 994 mother–child pairs from Rotterdam, the Netherlands, we obtained serum concentrations of a range of metabolite groups involved in energy metabolism, including amino acids (AA), non-esterified fatty acids (NEFA), phospholipids (PL), and carnitines (Carn) in maternal blood in early-pregnancy, and child’s (cord-) blood at birth and at age 10 years. We provide a detailed description of the data acquisition, processing and data structure and examined the relationships between metabolite profiles of pregnant women and their children at birth and in childhood.

## Methods

### Study population

The Generation R Study is a multi-ethnic population-based prospective cohort study from fetal life until adulthood in Rotterdam, the Netherlands, described in detail previously (Kooijman et al. 2016). The study was approved by the Medical Ethical Committee of the Erasmus Medical Center, University Medical Center, Rotterdam (MEC 198.782/2001/31). Written informed consent was obtained from all mothers at enrollment in the study. Measurement of conventional biomarkers of metabolic status in pregnancy and childhood has been described previously (Adank et al. 2019; Geurtsen et al. 2019; Silva et al. 2019). For metabolomics, 2,395 blood samples were analyzed from a subsample of 1041 Dutch mother–child pairs who had their blood drawn at birth (cord blood) and at least 1 other time point: early pregnancy (mother) or at the age of 10 years (child). A number of blood samples (n = 157) was excluded during data acquisition (e.g. low sample volumes, hemolytic samples) and processing (e.g. duplicate samples, high proportion of missing values, missing or non-Dutch ethnicity), leaving a total of 2,238 blood samples from 994 mother–child pairs available for analysis. Of these 994 mother–child pairs, a total of 814 mothers had early pregnancy data available, and 921 and 503 children had data available at birth and at the age of 10 years, respectively. Of all mothers included, 10 had a twin pregnancy. Metabolomics data was only available for one of the twins. Therefore, mothers with twin pregnancies were included only once in the dataset.

### Sample collection and processing

Maternal early-pregnancy non-fasting blood samples were obtained at enrollment in the study [median gestational age: 12.8 weeks (95% range 9.9, 16.9)] by research nurses at one of the dedicated research centers (Kruithof et al. 2014). Umbilical venous cord blood samples were collected directly after birth [median gestational age at birth: 40.3 weeks (95% range 36.6, 42.4)] by a midwife or obstetrician. Child’s non-fasting blood samples were obtained by research nurses at the 10-year follow-up visit to the research center [median age: 9.8 years (95% range 9.1, 10.6)]. All blood samples were transported to the regional laboratory (STAR-MDC), spun and stored at − 80 °C for further studies within a maximum of 4 h after collection. For metabolite measurements, blood serum samples were transported on dry ice to the Division of Metabolic and Nutritional Medicine of the Dr. von Hauner Children’s Hospital in Munich, Germany.

### Metabolite measurements

A targeted metabolomics approach was adopted to determine serum concentrations (µmol/L) of AA, NEFA, PL and Carn (Hellmuth et al. 2017b). Detailed information is given in Supplemental Text S1 and Table S1. Briefly, AA were analyzed with 1100 high-performance liquid chromatography (HPLC) system (Agilent, Waldbronn, Germany) coupled to a API2000 tandem mass spectrometer (AB Sciex, Darmstadt, Germany) (Harder et al. 2011). IUPAC-IUB Nomenclature was used for notation of AA (IUPAC-IUB Joint Commission on Biochemical Nomenclature 1984). NEFA, PL and Carn were measured with a 1200 SL HPLC system (Agilent, Waldbronn, Germany) coupled to a 4000QTRAP tandem mass spectrometer from AB Sciex (Darmstadt, Germany) (Hellmuth et al. 2012; Uhl et al. 2016). The analytical technique used is capable of determining the total number of total bonds, but not the position of the double bonds and the distribution of the carbon atoms between fatty acid side chains. We used the following notation for NEFA, PL and Carn.a:X:Y, where X denotes the length of the carbon chain, and Y the number of double bonds. The ‘a’ denotes an acyl chain bound to the backbone via an ester bond (‘acyl-’) and the ‘e’ represents an ether bond (‘alkyl-’). For analyses, we categorized metabolites in to general metabolite groups based on chemical structure (AA, NEFA, PC.aa, PC.ae, Lyso.PC.a, Lyso.PC.e, SM, Free Carn and Carn.a) and in detailed metabolite subgroups based on chemical structure and physiological and biological relevance (AA: BCAA, aromatic amino acids (AAA), essential AA, non-essential AA; NEFA, PC.aa, PC.ae, Lyso.PC.a, Lyso.PC.e and SM: saturated, mono-unsaturated, poly-unsaturated; Carn.a: short-chain, medium-chain, long-chain).

### Quality control and pre-processing

To assess the precision of the measurements, six quality control (QC) samples per batch were consistently measured between study samples. After exclusion of outliers, the coefficients of variation (CV; SD/mean) for each batch (intra-batch) and for all batches (inter-batch) of the QC samples were calculated for each metabolite. In line with previous studies (Hellmuth et al. 2017a; Lindsay et al. 2015; Rauschert et al. 2017b; Shokry et al. 2019), for each metabolite we excluded batches with an intra-batch CV higher than 25%. Data on complete metabolites were excluded for metabolites with inter-batch CV higher than 35% or if less than 50% of the batches passed the QC (i.e. had an intra-batch CV lower than 25%). To correct for batch effects, the participant data at each time point were median corrected by dividing the metabolite concentration by the ratio of the intra-batch median and the inter-batch median of the QC samples (Shokry et al. 2019). In line with previous studies, metabolites and participants with more than 50% of missing values were excluded (Hellmuth et al. 2017a; Shokry et al. 2019). Missing values in other participants were imputed using the Random Forest algorithm (R package *missForest*), which has been shown to perform well with MS data (Wei et al. 2018).

### Statistical analysis

First, we calculated the sum of individual metabolite concentrations per general and detailed metabolite group and per time point. In order to explore the variability of the metabolites between participants and between time points, we obtained the median (95% range) for the individual metabolites and the summed metabolite concentrations per general and detailed metabolite group per time point. To enable comparison between time points, only metabolites that were present at each time point were included in the summed variables. Second, we explored the dimensionality of the data, by conducting principal component analyses (PCA) at each time point separately. As log transformations did not sufficiently normalize the metabolite concentrations, we used square root transformations to normalize metabolite concentrations. These normalized metabolite concentrations were subsequently standardized by calculating standard deviation scores [SDS; (observed value − mean)/SD]. Third, as we considered PCA not informative for describing the information contained in our dataset, we further explored the correlation structure of the data by calculating pairwise Pearson’s correlations coefficients between all individual metabolites within each time point and between individual metabolites at different points. These correlations within and between time points were visualized using two circos plots (R package *circlize*) (Chung et al. 2018; Gu et al. 2014). To facilitate presentation, the first plot only includes correlation coefficients < − 0.15 and > 0.15. To display correlation coefficients that are at least of weak magnitude, the second plot displays correlation coefficients < − 0.30 and > 0.30 (Hinkle et al. 2003). To obtain further insight in possible metabolic pathways, we additionally presented correlations between metabolites within a time point as correlation networks, as correlations between metabolites were strongest within time points (Rosato et al. 2018). To provide a numerical summary of the strength of the correlations, we additionally constructed heatmaps of the median absolute correlation coefficients within general and detailed metabolite groups and between general and detailed metabolite groups at each of the time points separately. We calculated the correlation coefficients for correlations between individual metabolites at different time points. Correlations of 0–0.29, 0.3–0.49, 0.5–0.69, 0.7–0.89, and 0.9–1.0 were considered to be very low, low, moderate, high and very high, respectively (Hinkle et al. 2003). As sex differences in metabolite concentrations may exist (Ellul et al. 2019), we repeated steps one and three stratified by child’s sex. The statistical analyses were performed using R version 3.3.4 (R Foundation for Statistical Computing) (R Core Team 2015).

## Results

### Description of the study population

Table 1 provides general characteristics of the study population. Of the 994 mother–child pairs with data available, 125 (12.6%), 494 (49.7%) and 375 (37.7%) had data available at 1, 2, or 3 time points, respectively.

**Table 1:** General characteristics of the study population

	Total sample	Time point		
		Mother early pregnancy	Child at birth	Child age 10 years
	n = 994	n = 814	n = 921	n = 503
(Gestational-) age at blood sample, median (95% range), weeks/years	NA	12.8 (9.8, 16.9)^a^	40.3 (36.6, 42.4)^a^	9.8 (9.1, 10.6)^b^
Maternal characteristics				
Age, mean (SD), years	31.5 (4.2)	31.4 (4.1)	31.5 (4.1)	31.9 (3.9)
Education level, n (%)				
Primary	21 (2.1)	15 (1.9)	20 (2.2)	6 (1.2)
Secondary	342 (34.7)	285 (35.2)	324 (35.4)	165 (32.9)
Higher	623 (63.2)	509 (62.9)	570 (62.4)	330 (65.9)
Pre-pregnancy BMI, median (95% range), kg/m^2^	22.5 (18.5, 33.3)	22.6 (18.5, 33.3)	22.5 (18.5, 33.5)	22.4 (18.6, 33.4)
Early pregnancy glucose, mean (SD), mmol/L	4.4 (0.8)	4.4 (0.8)	NA	NA
Early pregnancy total cholesterol, mean (SD), mmol/L	4.9 (0.8)	4.7 (0.8)	NA	NA
Early pregnancy triglycerides, median (95% range), mmol/L	1.2 (0.7, 2.5)	1.3 (0.7, 2.5)	NA	NA
Early pregnancy HDL-cholesterol, mean (SD), mmol/L	1.8 (0.3)	1.8 (0.3)	NA	NA
Early pregnancy LDL-cholesterol, mean (SD), mmol/L	2.5 (0.7)	2.5 (0.7)	NA	NA
Child’s characteristics				
Gestational age at birth, median (95% range), weeks	40.3 (36.4, 42.4)	40.3 (36.1, 42.4)	40.3 (36.6, 42.4)	40.3 (37.1, 42.4)
Birth weight, median (95% range), g	3545 (2465, 4546)	3550 (2470, 4549)	3548 (2500, 4560)	3560 (2591, 4509)
Sex, male (%)	532 (53.5)	441 (54.2)	497 (54.0)	259 (51.4)
Body mass index at age 10 years, median (95% range), kg/m^2^	16.7 (14.0, 22.2)	NA	NA	16.6 (14.1, 21.8)
Glucose at age 10 years, mean (SD), mmol/L	5.3 (0.9)	NA	NA	5.3 (0.9)
Total cholesterol at age 10 years, mean (SD), mmol/L	4.3 (0.6)	NA	NA	4.3 (0.6)
Triglycerides at age 10 years, median (95% range), mmol/L	0.9 (0.4, 2.4)	NA	NA	0.9 (0.4, 2.4)
HDL-cholesterol at age 10 years, mean (SD), mmol/L	1.5 (0.3)	NA	NA	1.5 (0.3)
LDL-cholesterol at age 10 years, mean (SD), mmol/L	2.3 (0.6)	NA	NA	2.3 (0.6)

Values represent mean (SD), median (95% range) or number of participants (valid %)

*NA* not applicable

^a^Represents gestational age in weeks

^b^Represents age in years

### Variability

**Fig. 1:**
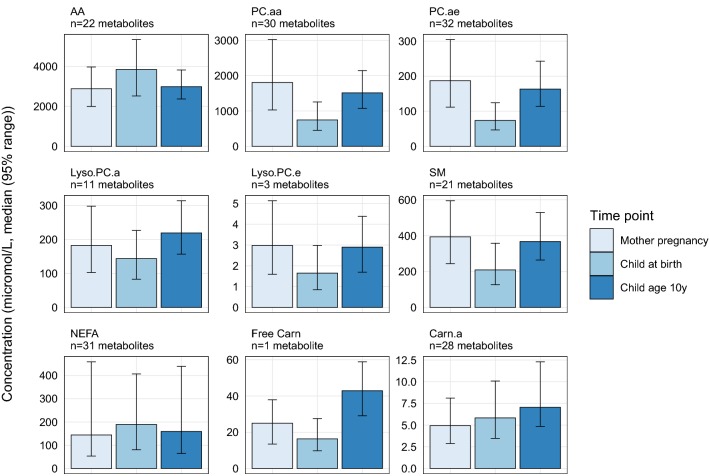
Median metabolite concentrations by metabolite group and time point. Values represent the median (95% range) of the sum of the individual metabolite concentrations in each of the metabolite groups, by time point. Sums only include metabolites with data at all time points, and therefore do not include concentrations of lyso.PC.a.C20.2, PC.aa.C32.3, PC.aa.C34.5, PC.aa.C36.0, PC.aa.C38.2, PC.aa.C40.3, PC.ae.C34.4, SM.a.C30.1, SM.a.C35.0, SM.a.C37.1, SM.a.C38.3, SM.a.C39.2, SM.a.C40.5, SM.a.C42.4, SM.a.C44.6, SM.e.C36.2, and SM.e.C40.5. SM includes SM.a plus one SM.e. *AA* amino acids, *PC.aa* diacyl-phosphatidylcholines, *PC.ae* acyl-alkyl-phosphatidylcholines, *Lyso.PC.a* acyl-lysophosphatidylcholines, *Lyso.PC.e* alkyl-lysophosphatidylcholines, *SM* sphingomyelines, *NEFA* non-esterified fatty acids, *Free Carn* free carnitine, *Carn.a* acyl-carnitines

Data was available on a total of 196 metabolites, of which 195 metabolites in early pregnancy, 194 metabolites at birth and 181 metabolites at child’s age 10 years. Descriptive information is provided in Supplemental Table S2. Figure 1 shows that the summed metabolite concentration for each general metabolite group varied considerably by time point. Summed concentrations of PC.aa, PC.ae, Lyso.PC.e and SM were highest in maternal blood in early pregnancy, compared to the other time points. Summed concentrations of AA and NEFA were highest in children at birth, whereas summed concentrations of Lyso.PC.a, Free Carn and Carn.a were highest in children of age 10. Supplemental Table S2 gives the summed concentrations of the detailed metabolite subgroups, which followed similar patterns. Supplemental Fig. S1 shows that the summed metabolite concentrations did not differ by child’s sex.

### Dimensionality

Table 2 shows the number of components (PCs) required to explain percentages of cumulative variance at each time point. At each time point, a relatively high number of PCs was needed to explain > 85% of the variance. The obtained PCs did not clearly represent specific metabolic pathways (Supplemental Figs. S2–S4).

**Table 2:** Number of components required to explain percentages of cumulative proportions of variance at each time point

Time point	Number of metabolites	Number of PCs				
		50%	75%	85%	95%	99.5%
Mother early pregnancy	195	3	15	35	88	163
Child at birth	194	4	21	46	101	169
Child age 10 years	181	6	27	50	98	157

Values represent the number of principal components (PCs) derived from principal component analyses required to explain 50, 75, 85, 95, and 99.5%, respectively, of the variances of the data at each of the time points

### Correlation structure

**Fig. 2:**
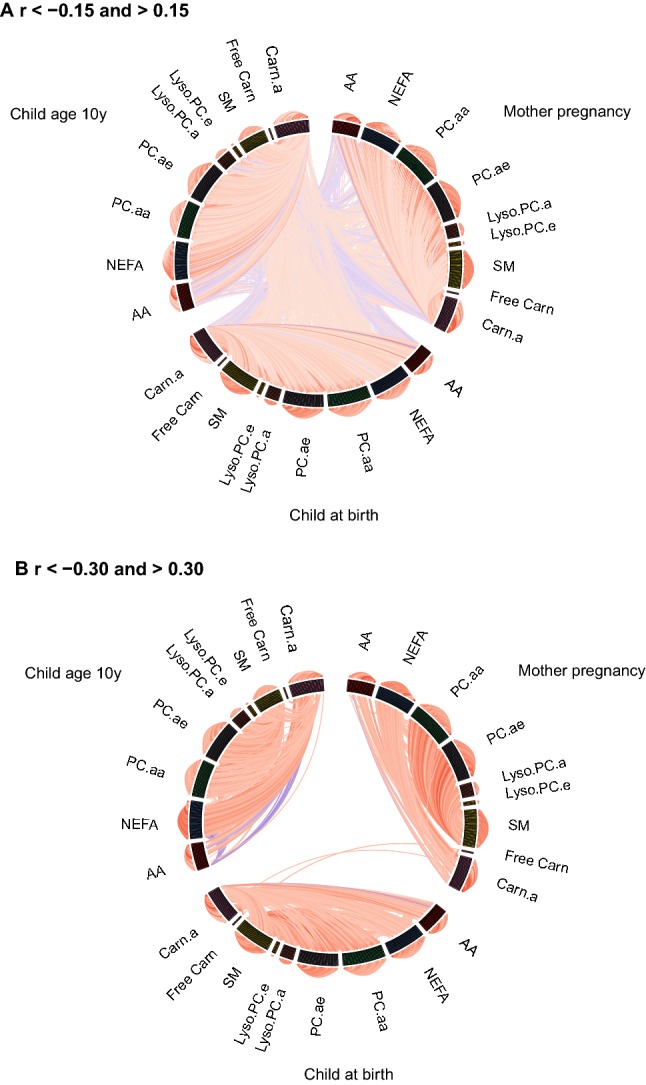
Circos plots of correlations between individual metabolite concentrations. Lines represent Pearson’s correlation coefficients between the individual metabolite concentrations within metabolite groups (outer circle), between metabolite groups (inner circle) and between time points (lines going through the middle of the circle). Red lines represent positive correlations and blue lines represent negative correlations. The brightness of the lines indicates the strength of the correlations, with brighter colors for stronger correlations. **a** Shows only correlation coefficients lower than − 0.15 and higher than 0.15 and **b** shows only correlation coefficients lower than − 0.30 and higher than 0.30. *AA* amino acids, *NEFA* non-esterified fatty acids, *PC.aa* diacyl-phosphatidylcholines, *PC.ae* acyl-alkyl-phosphatidylcholines, *Lyso.PC.a* acyl-lysophosphatidylcholines, *Lyso.PC.e* alkyl-lysophosphatidylcholines, *SM* sphingomyelines, *Free Carn* free carnitine, *Carn.a* acyl-carnitines

Figure 2 provides an overview of the correlations between individual metabolite concentrations within general metabolite groups (outer circle), between metabolites concentrations in different general metabolite groups (inner circle) and metabolite concentrations at different time points (lines going through the middle of the circle). Figure 2a shows all correlations lower than − 0.15 or higher than 0.15, whereas Fig. 2b shows all correlations lower than − 0.30 or higher than 0.30. At all time points, relatively high correlations were observed of individual metabolites within general metabolite groups and between individual metabolites from the different PL groups (PC.aa, PC.ae, Lyso.PC.a, Lyso.PC.e, and SM), between AA and Carn.a, and between NEFA and Carn.a. These correlations were mainly of positive direction, except some of the correlations between AA and Carn.a. In children of age 10 years only, some of the AA were negatively correlated with NEFA. Presentation of these correlations within pregnant women, children at birth and children at age 10 years as correlation networks showed the strongest correlations for individual metabolites within general metabolite groups (Supplemental Fig. S5).

**Fig. 3:**
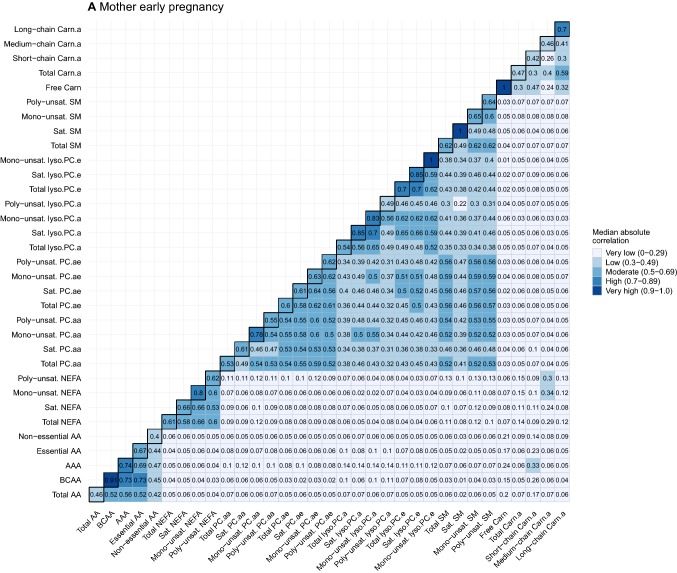

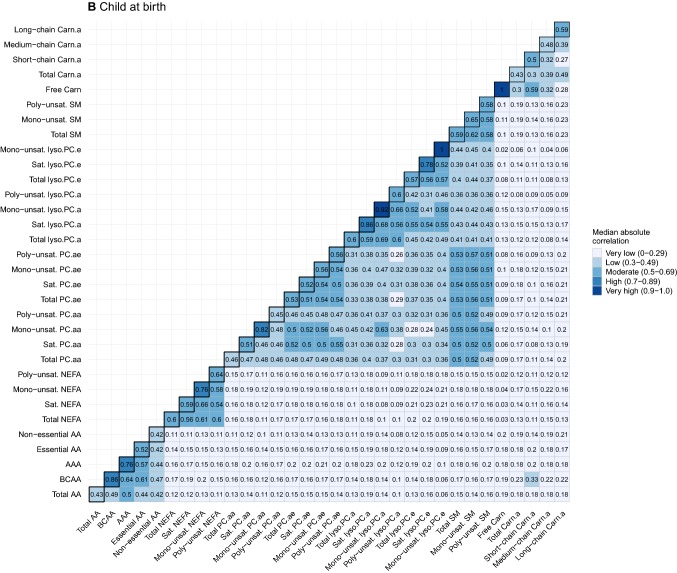

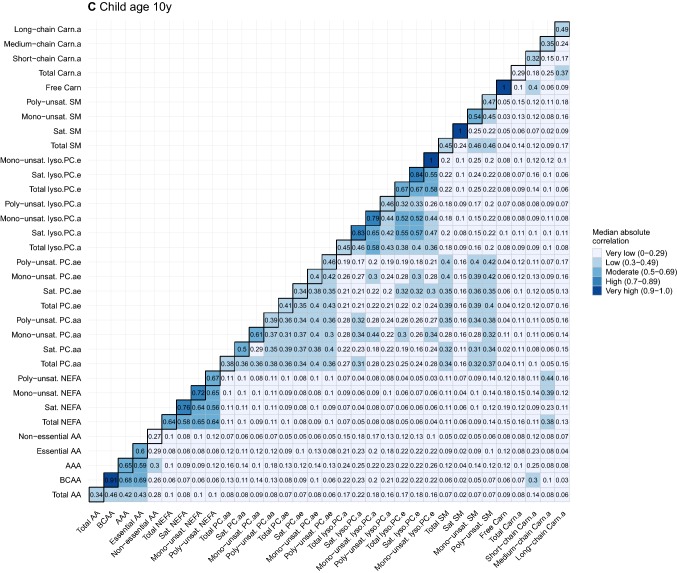
Heatmaps of median absolute correlation of individual metabolites within and between metabolite groups by time point. Values represent median absolute correlation coefficients of individual metabolite concentrations within metabolite groups (diagonal) and between metabolite groups (off-diagonal) by time point. Mono-unsaturated lyso.PC.e, saturated SM and Free Carn include 1 metabolite, resulting in a correlation coefficient of 1 for within-group correlations. For child at birth, no data on saturated SM is available. *AA* amino acids, *BCAA* branched-chain amino acids, *AAA* aromatic amino acids, *NEFA* non-esterified fatty acids, *PC.aa* diacyl-phosphatidylcholines, *PC.ae* acyl-alkyl-phosphatidylcholines, *Lyso.PC.a* acyl-lysophosphatidylcholines, *Lyso.PC.e* alkyl-lysophosphatidylcholines, *SM* sphingomyelines, *Free Carn* free carnitine, *Carn.a* acyl-carnitines, *Sat.* Saturated, *Unsat.* Unsaturated

To provide further insight into the strength of these correlations, Fig. 3a–c summarizes the correlations as the median absolute correlations of individual metabolites within general and detailed metabolite groups (diagonal) per time point. The median absolute correlations between general and detailed metabolite groups per time point are shown off-diagonal. Median absolute correlations within general and detailed metabolite groups at the same time point were low to high, and ranged between *r* = 0.27 and *r* = 0.92. The strength of these within-group median correlations differed by detailed metabolite subgroup, with BCAA, mono-unsaturated NEFA, mono-unsaturated PC.aa, mono-unsaturated PC.ae, saturated Lyso.PC.e, mono-unsaturated SM and long-chain Carn.a generally having the highest median correlations within their respective general groups. Median absolute correlations between subgroups of different metabolite groups were very low, except for correlations between NEFA detailed subgroups and medium-chain Carn.a in early pregnancy (*r* ranging between 0.24–0.34) and at age 10 years (*r* ranging between 0.23–0.44), between BCAA and AAA and short-chain Carn.a in early pregnancy (*r* = 0.26 and *r* = 0.33, respectively) and at age 10 years (*r* = 0.30 and *r* = 0.25, respectively), and between BCAA and short-chain Carn.a (*r* = 0.33) at birth.

Table 3 shows correlations of individual metabolites between each of the time points. For presentation purposes, this table only gives the 30 strongest correlations at each combination of time points, all correlations given in Supplemental Table S3. Correlations between early pregnancy and child’s metabolites at birth mainly included Free Carn, and Carn.a, and some long chain- and very long chain NEFA and some mainly non-essential AA. Correlations between early pregnancy and child age 10 years included a few AA and some PC.aa. In children, metabolites correlated between birth and age 10 years mainly included phospholipids. Almost all correlations were very weak, except the correlations between early pregnancy and birth Free Carn (*r* = 0.35) and Carn.a C9:0 (*r* = 0.32). Supplemental Figs. S6 and S7 show that the correlations between individual metabolites and median absolute correlations, respectively, were similar for boys and girls.

**Table 3:** Correlations of individual metabolite concentrations between time points, subset of 30 strongest correlations

A. Mother early pregnancy—child at birth				B. Mother early pregnancy—child age 10 years				C. Child at birth—child age 10 years			
Metabolite	n	r	p-value	Metabolite	n	r	p-value	Metabolite	n	r	p-value
Free Carn	749	0.35	< 0.001	Free Carn	413	0.24	< 0.001	Cit	457	0.24	< 0.001
Carn.a C9:0	749	0.32	< 0.001	PC.aa C36:6	413	0.23	< 0.001	SM.a C34:2	457	0.21	< 0.001
Carn.a C8:1	749	0.28	< 0.001	Carn.a C14:2	413	0.22	< 0.001	lyso.PC.a C22:6	457	0.20	< 0.001
Carn.a C4:0	749	0.26	< 0.001	Cit	413	0.21	< 0.001	SM.a C42:6	457	0.20	< 0.001
NEFA C26:0	749	0.24	< 0.001	Orn	413	0.21	< 0.001	His	457	0.19	< 0.001
Gly	749	0.22	< 0.001	Asn	413	0.17	< 0.001	Carn.a C4:0	457	0.19	< 0.001
Carn.a C10:1	749	0.22	< 0.001	PC.aa C38:4	413	0.17	0.001	PC.aa C38:6	457	0.18	< 0.001
Carn.a C15:0	749	0.22	< 0.001	PC.aa C38:6	413	0.17	< 0.001	PC.ae C32:2	457	0.18	< 0.001
Carn.a C3:0	749	0.21	< 0.001	NEFA C24:4	413	0.16	0.001	SM.a C32:2	457	0.18	< 0.001
Cit	749	0.20	< 0.001	PC.aa C38:0	413	0.16	0.001	Free Carn	457	0.18	< 0.001
NEFA C20:5	749	0.20	< 0.001	PC.aa C36:5	413	0.15	0.002	PC.aa C36:5	457	0.17	< 0.001
Carn.a C8:0	749	0.20	< 0.001	PC.ae C34:3	413	0.15	0.002	PC.aa C38:0	457	0.17	< 0.001
NEFA C22:6	749	0.19	< 0.001	NEFA C20:5	413	0.14	0.004	PC.ae C34:1	457	0.17	< 0.001
Carn.a C2:0	749	0.19	< 0.001	NEFA C24:5	413	0.14	0.004	Orn	457	0.16	< 0.001
Carn.a C14:2	749	0.19	< 0.001	PC.aa C40:6	413	0.14	0.003	PC.aa C43:6	457	0.16	0.001
His	749	0.18	< 0.001	PC.aa C43:6	413	0.14	0.005	PC.ae C32:0	457	0.16	0.001
Carn.a C10:0	749	0.18	< 0.001	PC.ae C36:5	413	0.14	0.004	NEFA C26:1	457	0.15	0.001
Carn.a C18:2	749	0.18	< 0.001	PC.ae C40:0	413	0.14	0.005	NEFA C26:2	457	0.15	0.001
Carn.a C20:3	749	0.18	< 0.001	SM.a C35:1	413	0.14	0.005	PC.ae C38:6	457	0.15	0.001
Carn.a.C20.4	749	0.18	< 0.001	Carn.a C4:0	413	0.14	0.004	PC.ae C42:3	457	0.15	0.001
Pro	749	0.17	< 0.001	Carn.a C15:0	413	-0.14	0.004	SM.a C33:1	457	0.15	0.001
PC.ae C42:4	749	0.17	< 0.001	Carn.a C16:0	413	0.14	0.004	Carn.a C8:1	457	0.15	0.002
lyso.PC.a C22:6	749	0.17	< 0.001	Ala	413	0.13	0.007	NEFA C20:5	457	0.14	0.003
Carn.a C12:0	749	0.17	< 0.001	Thr	413	0.13	0.007	NEFA C24:1	457	0.14	0.003
NEFA C26:1	749	0.16	< 0.001	PC.ae C30:0	413	0.13	0.007	NEFA C24:4	457	0.14	0.003
PC.aa C44:12	749	0.16	< 0.001	PC.ae C38:0	413	0.13	0.009	PC.ae C42:5	457	0.14	0.002
Ala	749	0.15	< 0.001	PC.ae C40:1	413	0.13	0.009	PC.ae C42:6	457	0.14	0.003
Phe	749	0.15	< 0.001	PC.ae C42:5	413	0.13	0.008	SM.a C32:1	457	0.14	0.004
PC.aa C38:6	749	0.15	< 0.001	Carn.a C16:0.Oxo	413	0.13	0.008	SM.a C36:2	457	0.14	0.002
lyso.PC.a C20:5	749	0.15	< 0.001	Gln	413	0.12	0.018	NEFA C22:3	457	0.13	0.006

Values represent Pearson’s correlation coefficients (r), and corresponding p-values and number of participants for correlations between metabolites at different time points. For presentation purposes, only the 30 strongest correlations at each combination of time points were presented. A complete list of correlations is given in Supplemental Table S3

## Discussion

We described the data acquisition, processing and structure of the metabolomics data available in the Generation R Study and assessed the relationships between metabolite profiles of pregnant women and their children at birth and in childhood. Metabolite concentrations vary considerably between pregnant women and their children at birth and at the age of 10 years. The individual metabolites correlate within groups of metabolites with similar chemical structures, but to a lesser extent between groups of metabolites with different chemical structures. The correlations of individual metabolites between pregnant women and their children at birth and age 10 years are relatively low.

### Interpretation of main findings

Metabolomics studies targeting cardio-metabolic diseases have already been successfully applied in adults (Newgard 2017; Rangel-Huerta et al. 2019; Ussher et al. 2016), but only a limited number of metabolomics studies have been performed on the early origins of these diseases (Hivert et al. 2015; Rauschert et al. 2017a). We obtained intergenerational metabolomics data at three different time points during pregnancy and postnatal life, that may provide more detailed insights in the early origins of cardio-metabolic disease, the underlying mechanisms and identify potential novel biomarkers.

Maternal metabolic profile during pregnancy might influence fetal metabolic profile, either directly through placental transfer, or indirectly by influences on hormone levels or placental function (Hivert et al. 2015). Maternal blood metabolite concentrations generally tend to decrease across pregnancy, likely reflecting increased circulating volume, tissue biosynthesis and placental uptake (Lindsay et al. 2015). Fetal metabolite concentrations are the result of both placental transfer and endogenous synthesis. Concentrations of AA, Carn and NEFA, particularly long-chain poly-unsaturated fatty acids (LC-PUFA), tend to be higher in fetal blood than in maternal blood (Larque et al. 2011; Regnault et al. 2002; Schmidt-Sommerfeld et al. 1985). This might be indicative of an active transport mechanism across the placenta or increased fetal synthesis. Although the large time differences between the metabolite measurements in our study should be noted and preclude direct conclusions about placental transfer, our observation that the summed concentrations of AA, NEFA and Carn.a were higher in cord blood than in maternal early pregnancy blood is in line with these previous studies. The lower PL concentrations observed in cord blood in comparison to maternal early pregnancy blood might be explained by the fact that PL do not cross the placenta, but are hydrolyzed to NEFA that in turn cross the placental barrier (Herrera and Ortega-Senovilla 2010; Larque et al. 2011; Rice et al. 1998). Relatively high correlations between individual metabolites within known general and detailed metabolite subgroups in pregnant women as well as in cord blood were observed, as expected from the shared precursors and biosynthesis pathways. However, correlations of individual metabolites between these two time points were relatively weak. These results are in line with those from a multi-ethnic study among 1600 participants that showed mostly weak correlations of these metabolites between maternal blood at 28 weeks of gestation and cord blood (Lowe et al. 2017). In our study, there is a large time difference between the metabolite measurements in mothers and newborns. Therefore, the relatively low correlations between maternal and cord blood metabolites might result from changes in metabolism in both pregnant women and the fetus that occur throughout pregnancy (Herrera and Ortega-Senovilla 2010; Lindsay et al. 2015). In addition, placental transfer of nutrients throughout pregnancy is tightly regulated by various transport mechanisms to ensure stable fetal metabolite concentrations at the expense of variations in maternal metabolite concentrations (Larque et al. 2013; Rossary et al. 2014). The relatively high correlations for carnitines in our study might be explained by the main source of carnitines for the fetus being placental transfer, rather than endogenous synthesis (Alexandre-Gouabau et al. 2013). Thus, individual metabolite concentrations correlate within mothers and newborns, but barely between mothers and newborns. This might result from changes in maternal and fetal metabolism throughout pregnancy and from tightly regulated active trans-placental transport mechanisms resulting in distinct metabolite profiles in pregnant women and their children at birth.

Less is known about the metabolite profiles from birth throughout childhood and the influence of maternal metabolite profiles in pregnancy on these profiles. A study among 127 children from Sweden showed that concentrations of conventional lipids, including total cholesterol, LDL cholesterol and HDL cholesterol increased between the age of 6 months and 4 years, whereas triglyceride concentrations decreased (Ohlund et al. 2011). A study among 500 children and adolescents aged 0 to 19 years observed that concentrations of AA, NEFA, and Carn.a dropped after the neonatal period. However, some of these Carn.a increased again from the age of 7 years and returned to neonatal concentrations at age 19 years (Teodoro-Morrison et al. 2015). A large familial resemblance in metabolite concentrations has been suggested, which seems to be largely genetic (Draisma et al. 2013; Kettunen et al. 2016; Rueedi et al. 2014). In cross-sectional studies, correlations of metabolites between parents and their offspring vary strongly, ranging from weak to relatively strong (Ellul et al. 2019; Halvorsen et al. 2015; Ohlund et al. 2011). Partly in line with these previous studies, we observed that AA and NEFA concentrations were lower in childhood as compared to cord blood samples, whereas concentrations of PL and Carn were higher in childhood. However, the correlations between individual metabolite concentrations of children at birth and at the age of 10 years as well as between mothers in early pregnancy and their children at the age of 10 years were very weak. This might be explained by the large timespan between the measurements. Also, previous research has indicated that metabolite concentrations are highly influenced by nutritional factors, physical activity and the gut microbiome (Hellmuth et al. 2019; Lau et al. 2018; Palmnas et al. 2018; Pedersen et al. 2016; Wang et al. 2011). Differences in these factors between mothers and their children and over time might explain the weak correlations between different time points. Previous studies observed sex differences in metabolite concentrations in both children and adults (Ellul et al. 2019; Teodoro-Morrison et al. 2015). We did not observe metabolite concentrations to vary between the sexes. This could be explained by the relatively young age of the participants, as sex differences in metabolite concentrations have been shown to be more pronounced in adolescence and adulthood (Ellul et al. 2019; Teodoro-Morrison et al. 2015). Thus, correlations between individual metabolites between pregnant women and their children at school-age and within children over time are very low. This might suggest strong influences of external factors and limited intergenerational correlations of metabolite profiles.

We provided the first explorative analyses of a unique large longitudinal dataset consisting of metabolomics data of pregnant women and their children at birth and in childhood, and studied correlations between a large number of metabolites at these different time points. Not much is known yet about the correlations of metabolites between pregnant women and their children and the metabolite profiles in children from birth until childhood. We observed relatively low correlations of metabolite concentrations between time points. We explored whether offspring sex affected these correlations as this is an important baseline characteristic which has been suggested to influence metabolite profiles in children and adults, but this did not affect our findings. Other maternal and childhood factors are likely to influence metabolite profiles in pregnant women, and the development of metabolites profiles from birth until childhood. Further studies are needed to obtain detailed insight into the influence of maternal and offspring socio-demographic, lifestyle and physical factors on the stability of metabolites profiles in pregnancy and from birth throughout childhood. Future studies using these data should take into account the correlations of metabolites within the same metabolite group. PCA, a data reduction approach commonly used in metabolomics, showed that the data were highly dimensional. This indicates that the variability in the data is difficult to capture in a lower number of components and that each metabolite contributes unique information. In addition, the obtained components did not describe specific metabolic pathways. Therefore, we do not consider the PCs informative in describing the information contained in this dataset. Given the high dimensionality of the data and the relatively high correlation of metabolites within metabolite groups, it seems that future studies focused on relating these data to exposures and outcomes of interest should analyze the data per individual metabolite and per metabolite group with structural, physiological and biological relevance. In addition, correlation networks based on correlations between individual metabolites or more advanced pathway analysis may be useful for identifying metabolic pathways involved in these associations. Due to the longitudinal nature of the data and the large amount of data on relevant exposures and outcomes available in the cohort, these data will form an important population-based resource for future metabolomics analyses on the developmental origins of cardio-metabolic disease.

### Methodological considerations

We obtained metabolomics data in a subgroup of the cohort, which consists of Dutch, relatively high educated and healthy participants, as compared to the full cohort (Kooijman et al. 2016). This may affect the generalizability of our sample to the full cohort and the general population. We adopted a targeted metabolomics approach, which enabled us to study absolute metabolite concentrations of metabolites known a priori to be relevant for obesity and cardio-metabolic disease. However, the targeted design might also be a limitation in future association studies, as relevant biological pathways might be missed. The blood samples used in our study were non-fasting and taken during non-fixed times of the day for logistic and ethical reasons (relatively young age of the children). Metabolite concentrations are dependent on fasting status. Fasting blood samples are usually preferred, as they are more reliable over time (Carayol et al. 2015). The use of non-fasting blood samples in our study might influence precision and power to detect associations of interest. However, non-fasting blood samples appear to be more informative of metabolic status throughout the day. Also, non-fasting lipids have been shown to perform equally or even better than fasting lipids in predicting the risk of cardiovascular disease (Nordestgaard et al. 2016). We therefore still consider non-fasting metabolite concentrations to be of interest. Due to the longitudinal design of the study, we were able to measure metabolite concentrations at 3 different time points during pregnancy and early postnatal life. However, due to the large time intervals between the blood samples and differences in the nature of the blood samples, small differences in procedures and handling of the blood samples may exist. As previous studies showed that different pre-storage temperatures and durations only minimally affected measured concentrations of most metabolites, we consider it unlikely that this strongly influenced our results.

## Conclusions

Metabolite concentrations vary between pregnant women and their children at birth and at the age of 10 years. Correlations of individual metabolites between pregnant women and their children at birth and in childhood are relatively low. This may suggest that unique metabolic profiles are present among pregnant women, newborns and school aged children, with limited intergenerational correlations between metabolite profiles. These data are an important population-based resource for future metabolomics analyses to address the early origins of cardio-metabolic disease.

## Electronic supplementary material

Below is the link to the electronic supplementary material.

Supplemental Text S1 (PDF 296 kb)

Supplemental Table S1 (XLSX 17 kb)

Supplemental Figure S1 (PDF 377 kb)

Supplemental Table S2 (XLSX 28 kb)

Supplemental Figure S2 (PDF 102 kb)

Supplemental Table S3 (XLSX 29 kb)

Supplemental Figure S3 (PDF 106 kb)

Supplemental Figure S4 (PDF 99 kb)

Supplemental Figure S5 (PDF 1357 kb)

Supplemental Figure S6 (PDF 665 kb)

Supplemental Figure S7 (PDF 1942 kb)

## Data Availability

The datasets generated and analyzed during the current study are not publicly available due to privacy restrictions, but are available from the corresponding author upon reasonable request.

## Abbreviations

AA: Amino acids
AAA: Aromatic amino acids
APCI: Atmospheric pressure chemical ionization
BCAA: Branched-chain amino acids
Carn: Carnitines
Carn.a: Acyl-carnitines
CV: Coefficient of variation
ESI: Electrospray ionization
FIA: Flow-injection analysis
Free Carn: Free carnitine
HDL: High-density lipoprotein
HPLC: High performance liquid chromatography
LC–MS/MS: Liquid chromatography tandem mass spectrometry
LC-PUFA: Long-chain poly-unsaturated fatty acids
LDL: Low-density lipoprotein
Lyso.PC.a: Acyl-lysophosphatidylcholines
Lyso.PC.e: Alkyl-lysophosphatidylcholines
MS: Mass spectrometry
NEFA: Non-esterified fatty acids
PC: Principal component
PCA: Principal component analysis
PC.aa: Diacyl-phosphatidylcholines
PC.ae: Acyl-alkyl-phosphatidylcholines
PL: Phospholipids
SD: Standard deviation
SDS: Standard deviation scores
SM: Sphingomyelines
QC: Quality control
