# Supplementary material for: A population-based resource for intergenerational metabolomics analyses in pregnant women and their children: the Generation R Study
Source: Metabolomics. 2020 Mar 23;16(4):43. doi: 10.1007/s11306-020-01667-1 (PMC7089886; doi:10.1007/s11306-020-01667-1)
Supplement: Supplementary file 1 — Supplemental Text S1 (PDF 296 kb) [file 11306_2020_1667_MOESM1_ESM.pdf]

Supplemental Text S1. Metabolite measurements

A targeted metabolomics approach was adopted to determine serum concentrations (µmol/L) of AA, NEFA, PL and Carn, as described previously (Hellmuth et al. 2017). Proteins of 50 µL serum were precipitated by adding 450 µL methanol including internal standards: labeled amino acid standards set A (NSK-A-1, Cambridge Isotope Laboratories (CIL), USA), 15N2-L-asparagine (NLM-3286-0.25, CIL, USA), indole-D5-L-tryptophan (DLM-1092-0.5, CIL, USA), U-13C16-palmitic acid (CLM-409-MPT-PK, CIL, USA), D3-acetyl-carnitine (DLM-754-PK, CIL, USA), D3-octanoyl-carnitine (DLM-755-0.01, CIL, USA) and D3-palmitoyl-carnitine (DLM-1263-0.01, CIL, USA), tridecanoyl-2-hydroxy-sn-glycero-3-phosphocholine (855476, Avanti Polar Lipids, USA) and 1,2-dimyristoyl-sn-glycero-3-phosphocholine (850345, Avanti Polar Lipids, USA). If sample volume was less than optimal, the concentrations were corrected by the respective factor. Sample volumes less than 25 µL were not used and considered missing. After centrifugation the supernatant was split into aliquots. AA were analyzed by liquid chromatography tandem mass spectrometry (LC-MS/MS) as described previously (Harder et al. 2011). An aliquot of the supernatant was used for the derivatization to AA butylester with hydrochloric acid in 1-butanol. After evaporation, the residues were dissolved in water/methanol (80:20; (v/v)) with 0.1% formic acid. The samples were analyzed with 1100 high-performance liquid chromatography (HPLC) system (Agilent, Waldbronn, Germany) equipped with 150 x 2.1 mm, 3.5 µm particle size C18 HPLC column (X-Bridge, Waters, Milford, USA) and 0.1% heptafluorobutyric acid as and ion pair reagent in the mobile phases A and B (A: water, B: methanol). Mass spectrometric (MS) detection was performed with an API2000 tandem mass spectrometer (AB Sciex, Darmstadt, Germany) equipped with an atmospheric pressure chemical ionization (APCI) source operating in positive ion ionization mode. IUPAC-IUB Nomenclature was used for notation of the AA (1984).

NEFA, PL and Carn were measured with a 1200 SL HPLC system (Agilent, Waldbronn, Germany) coupled to a 4000QTRAP tandem mass spectrometer from AB Sciex (Darmstadt, Germany) (Hellmuth et al. 2012; Uhl et al. 2016). NEFA were analyzed by injection of the supernatant to a LC-MS/MS operating in negative electrospray ionization (ESI) mode where they were separated by gradient elution on a 100 x 3.0 mm, 1.9 µm particle size Purusuit UPS Diphenyl column from Varian (Darmstadt, Germany) using 5 mM ammonium acetate in water as mobile phase A and acetonitrile/ isopropanol (80:20; (v/v)) as mobile phase B. NEFA species were quantified using GLC-85 reference standard mixture (Nu-Chek Prep, USA). PL were analyzed by flow-injection analysis (FIA) with LC-MS/MS coupled with ESI (Rauschert et al. 2016). The system was run in positive ionization mode with 5% water in isopropanol as mobile phase A and 5% water in methanol as mobile phase B. The analysis was performed for diacyl-phosphatidylcholines (PC.aa), acyl-alkyl-phosphatidylcholines (PC.ae) , acyl-

lysophosphatidylcholines (Lyso.PC.a), alkyl-lysophosphatidylcholines (Lyso.PC.e) and sphingomyelines (SM)). Carn (Free carnitine (Free Carn) and acyl-carnitines (Carn.a)) were analyzed by flow-injection analysis of the supernatant into a LC-MS/MS system using an isocratic elution with 76% isopropanol, 19% methanol and 5% water. The mass spectrometer was equipped with electrospray ionization and operated in positive ionization mode. PL and acyl-carn were quantified using aliquots of a commercial available lyophilized control plasma (ClinChek®, Recipe, Germany), where the concentrations have been determined by AbsoluteIDQ p150 Kit from Biocrates®, a previous published LC-MS/MS method (Uhl et al. 2011) and by in-house quantification with various standards. The calibrators used are given in **Supplemental Table S1**. The analytical technique used is capable of determining the total number of total bonds, but not the position of the double bonds and the distribution of the carbon atoms between fatty acid side chains. We used the following notation for NEFA, PL and Carn.a: X:Y, where X denotes the length of the carbon chain, and Y the number of double bonds. The 'a' denotes an acyl chain bound to the backbone via an ester bond ('acyl-') and the 'e' represents an ether bond ('alkyl-').

References

Harder, U., B. Koletzko, W. Peissner (2011). Quantification of 22 plasma amino acids combining derivatization and ion-pair LC-MS/MS. J Chromatogr B Analyt Technol Biomed Life Sci 879, 495-504.|

Hellmuth, C., M. Weber, B. Koletzko, W. Peissner (2012). Nonesterified fatty acid determination for functional lipidomics: comprehensive ultrahigh performance liquid chromatography-tandem mass spectrometry quantitation, qualification, and parameter prediction. Anal Chem 84, 1483-90

Hellmuth, C., O. Uhl, M. Standl, H. Demmelmair, J. Heinrich, B. Koletzko, et al. (2017). Cord Blood Metabolome Is Highly Associated with Birth Weight, but Less Predictive for Later Weight Development. Obes Facts 10, 85-100.

Rauschert, S., O. Uhl, B. Koletzko, F. Kirchberg, T. A. Mori, R. C. Huang, et al. (2016). Lipidomics Reveals Associations of Phospholipids With Obesity and Insulin Resistance in Young Adults. J Clin Endocrinol Metab 101, 871-9

Uhl, O., C. Glaser, H. Demmelmair, B. Koletzko (2011). Reversed phase LC/MS/MS method for targeted quantification of glycerophospholipid molecular species in plasma. J Chromatogr B Analyt Technol Biomed Life Sci 879, 3556-64

Uhl, O., M. Fleddermann, C. Hellmuth, H. Demmelmair, B. Koletzko (2016). Phospholipid Species in Newborn and 4 Month Old Infants after Consumption of Different Formulas or Breast Milk. PLoS One 11, e0162040
