# Supplementary material for: A population-based resource for intergenerational metabolomics analyses in pregnant women and their children: the Generation R Study
Source: Metabolomics. 2020 Mar 23;16(4):43. doi: 10.1007/s11306-020-01667-1 (PMC7089886; doi:10.1007/s11306-020-01667-1)
Supplement: Supplementary file 3 — Supplemental Figure S1 (PDF 377 kb) [file 11306_2020_1667_MOESM3_ESM.pdf]

**Figure S1. Median metabolite concentrations by metabolite group and time point, stratified by child's sex**

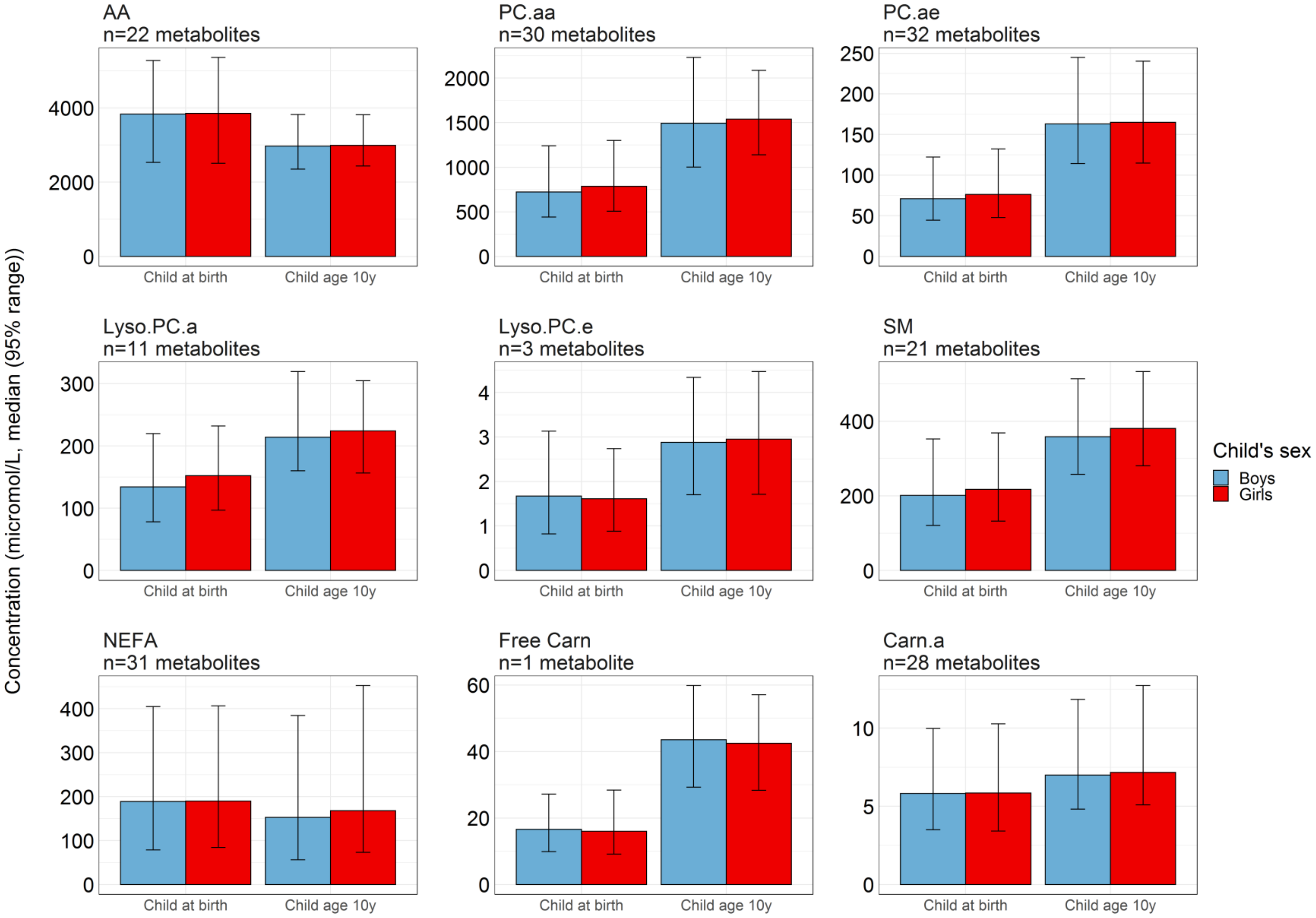


Values represent the median (95% range) of the sum of the individual metabolite concentrations in each of the metabolite groups, by time point and child's sex. Sums only include metabolites with data at all time points, and therefore do not include concentrations of lyso.PC.a.C20.2, PC.aa.C32.3, PC.aa.C34.5, PC.aa.C36.0, PC.aa.C38.2, PC.aa.C40.3, PC.ae.C34.4, SM.a.C30.1, SM.a.C35.0, SM.a.C37.1, SM.a.C38.3, SM.a.C39.2, SM.a.C40.5, SM.a.C42.4, SM.a.C44.6, SM.e.C36.2, and SM.e.C40.5. SM includes SM.a plus one SM.e. AA: amino acids, PC.aa: diacyl-phosphatidylcholines, PC.ae: acyl-alkyphosphatidylcholines, Lyso.PC.a: acyl-lysophosphatidylcholines, Lyso.PC.e: alkyl-lysophosphatidylcholines, SM: sphingomyelines, NEFA: non-esterified fatty acids, Free Carn: free carnitine, Carn.a: acyl-carnitines.
