# Supplementary material for: A population-based resource for intergenerational metabolomics analyses in pregnant women and their children: the Generation R Study
Source: Metabolomics. 2020 Mar 23;16(4):43. doi: 10.1007/s11306-020-01667-1 (PMC7089886; doi:10.1007/s11306-020-01667-1)

Mother early pregnancy − PC 1 Loadings

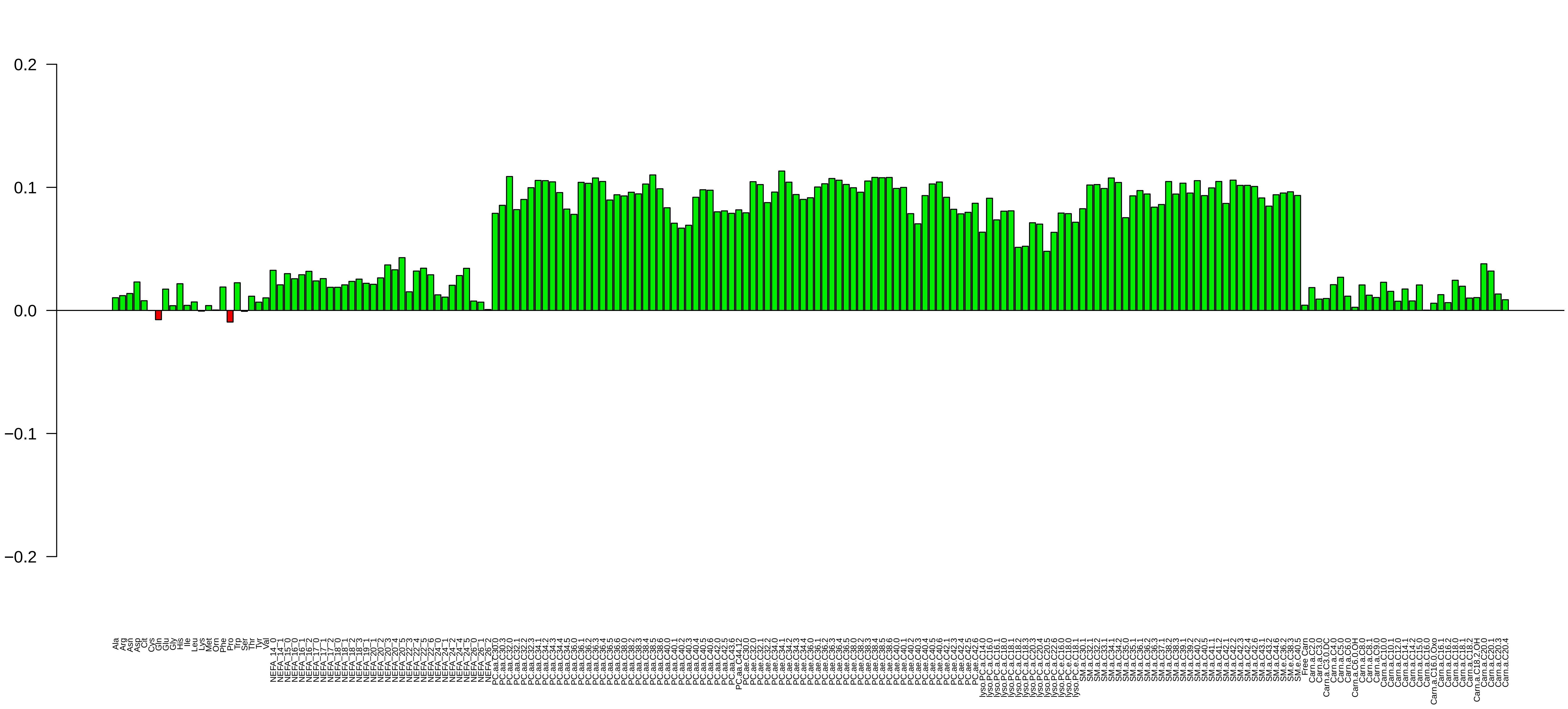

Mother early pregnancy − PC 2 Loadings

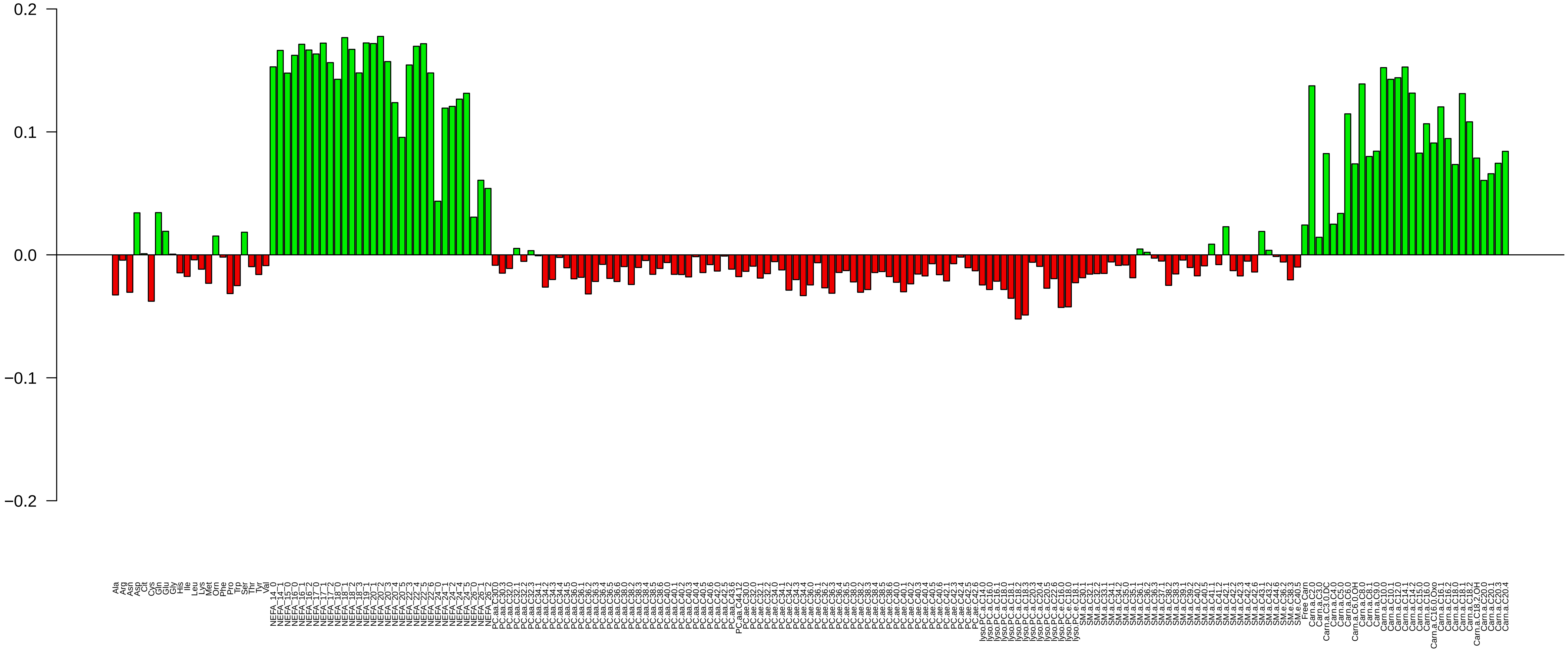

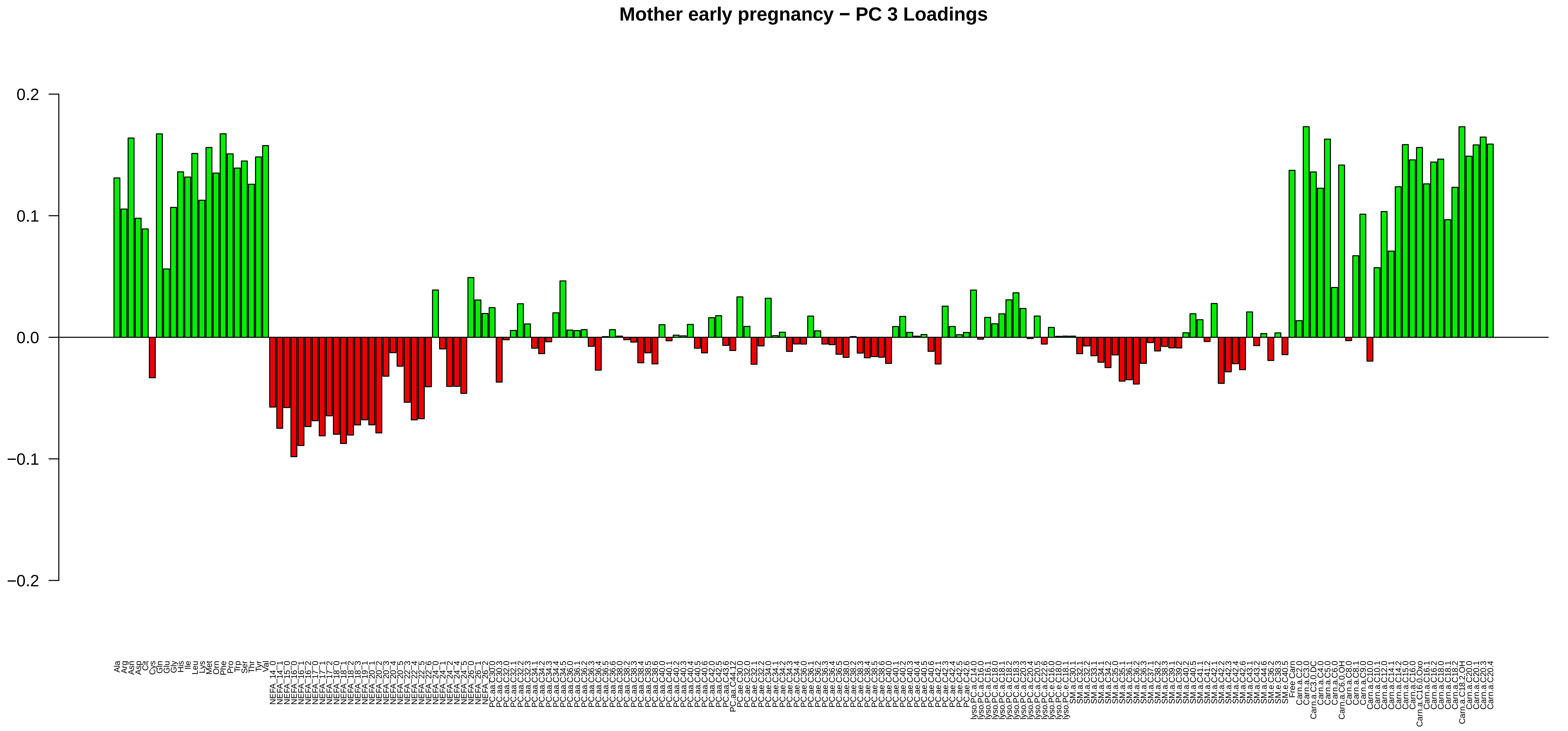
Mother early pregnancy − PC 3 Loadings
0.2
0.1
0.0
−0.1
−0.2

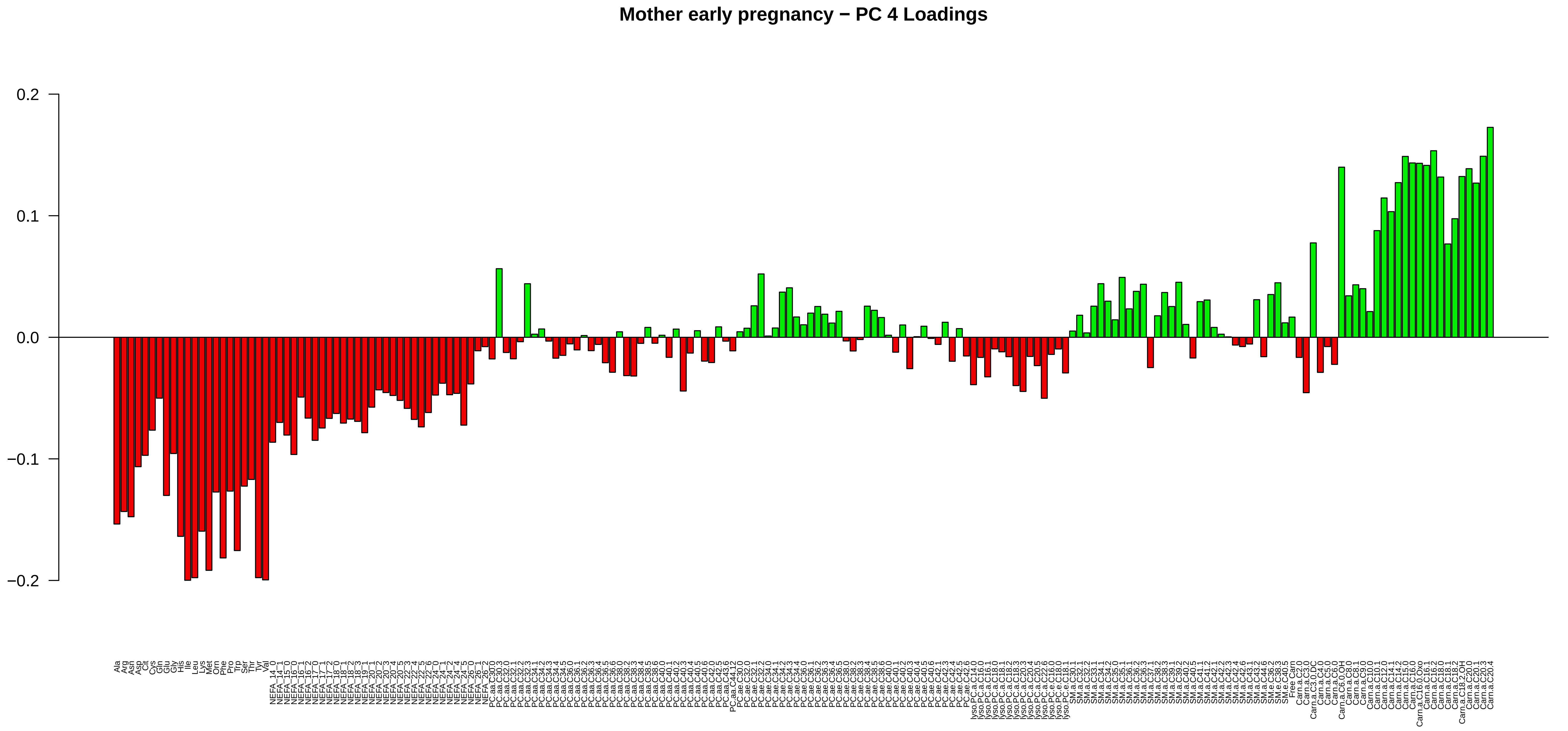
Mother early pregnancy − PC 4 Loadings
0.2
0.1
0.0
−0.1
−0.2

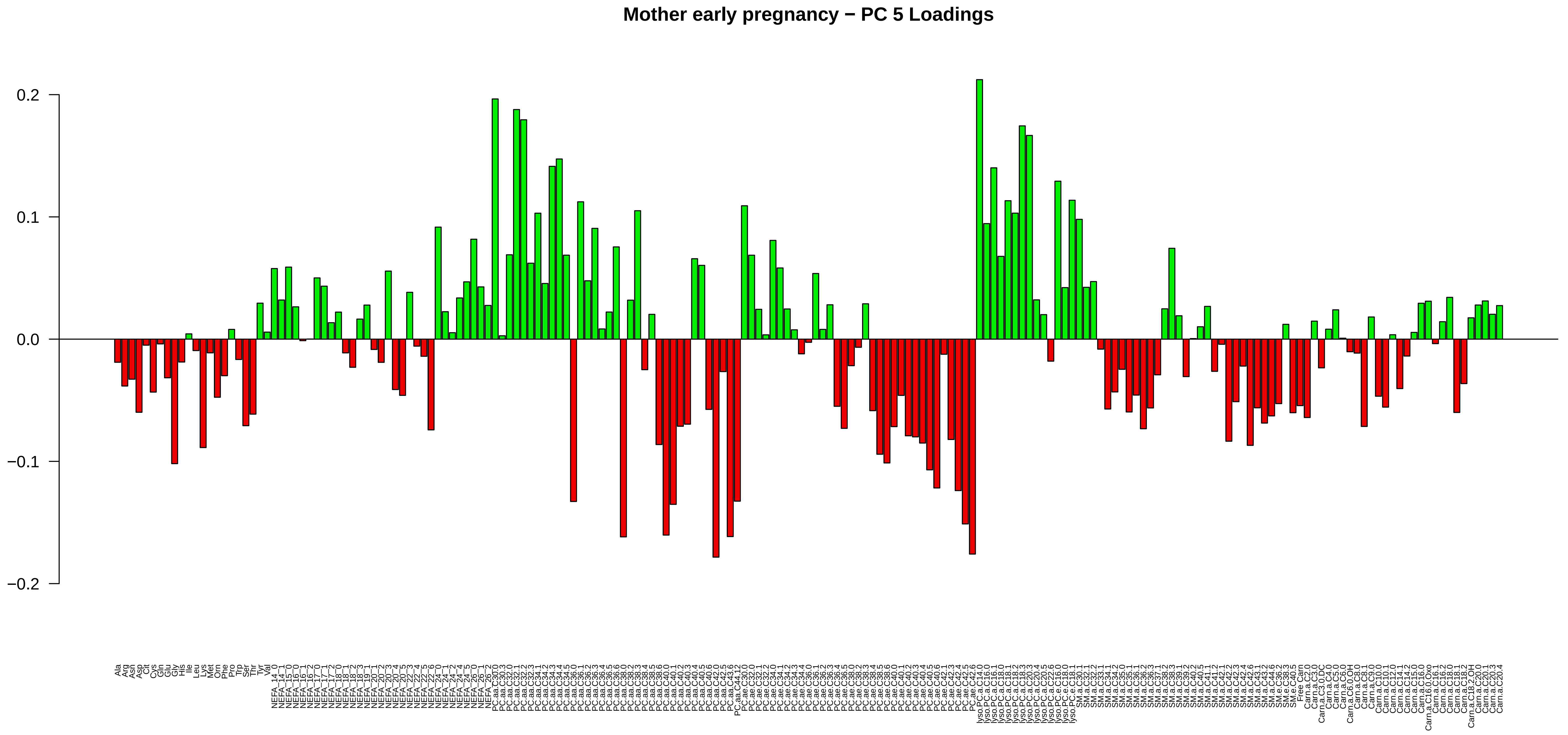
Mother early pregnancy − PC 5 Loadings
0.2
0.1
0.0
−0.1
−0.2

Mother early pregnancy − PC 6 Loadings

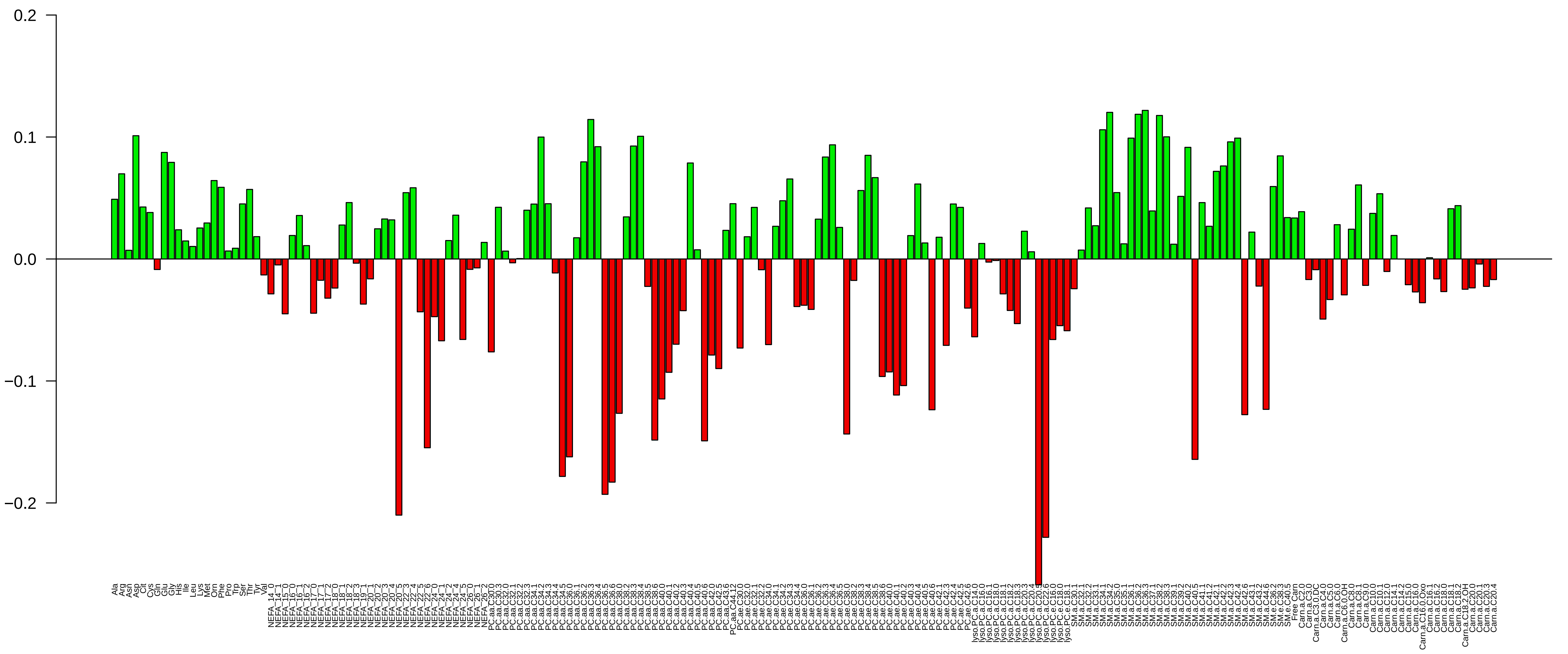

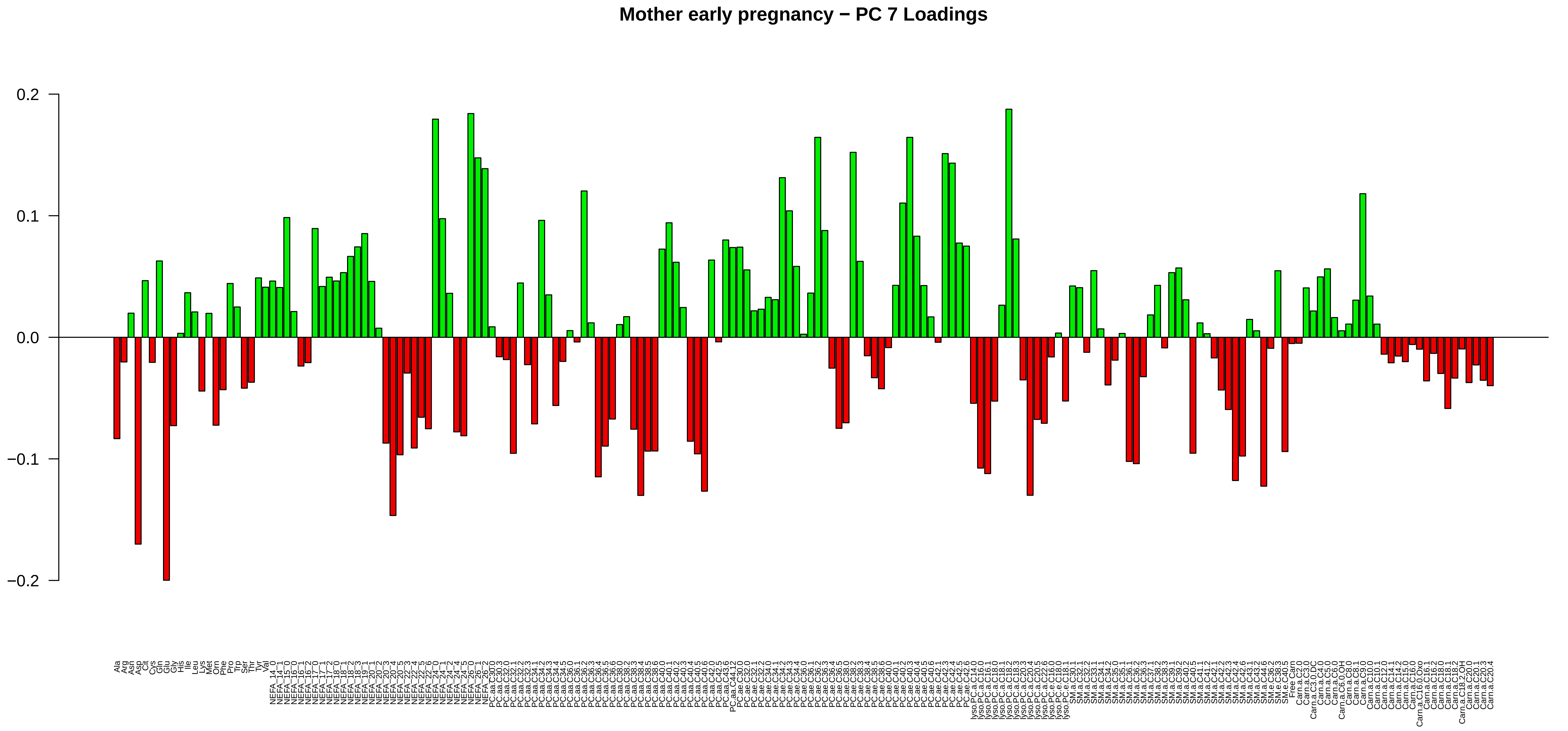
Mother early pregnancy − PC 7 Loadings
0.2
0.1
0.0
−0.1
−0.2

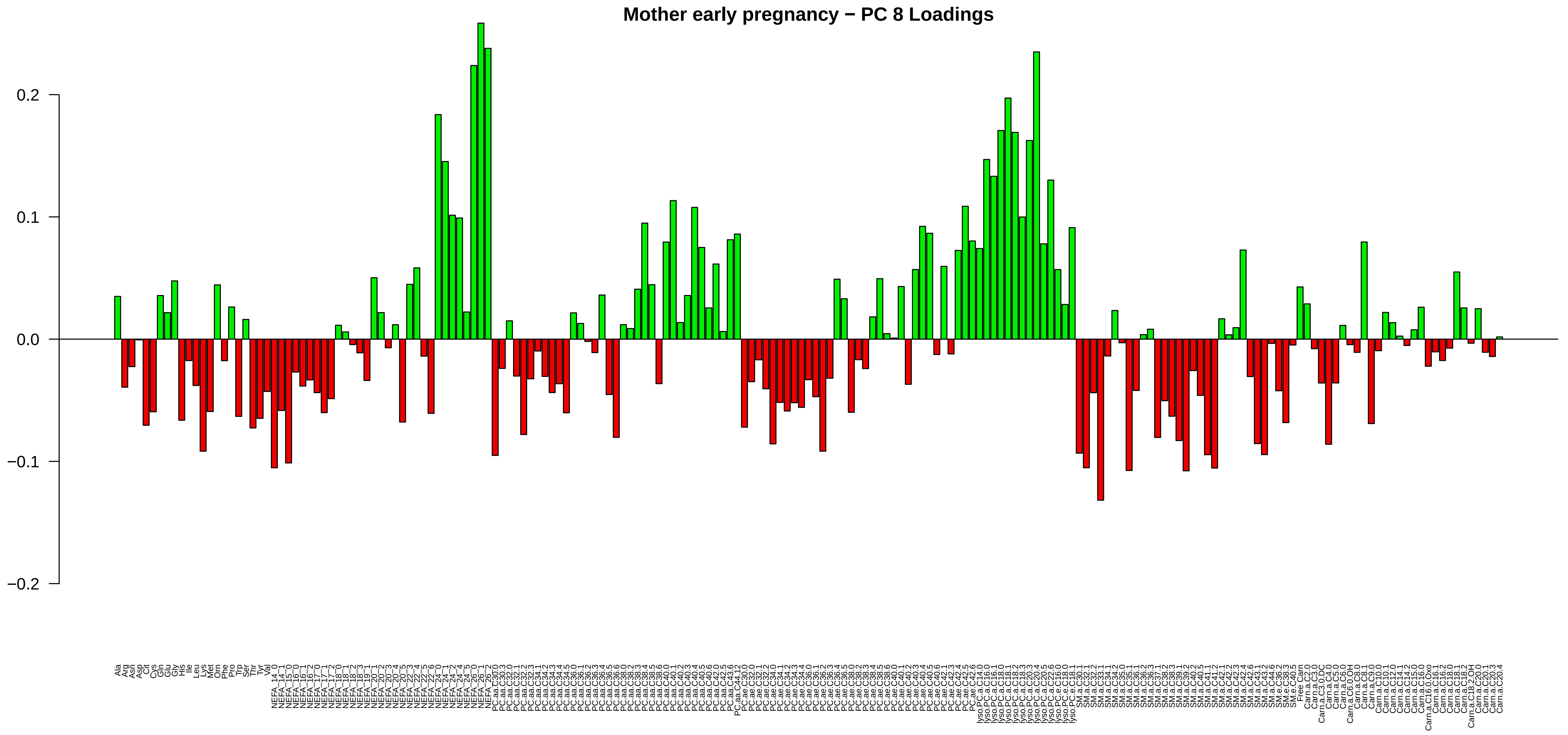
Mother early pregnancy − PC 8 Loadings
0.2
0.1
0.0
−0.1
−0.2

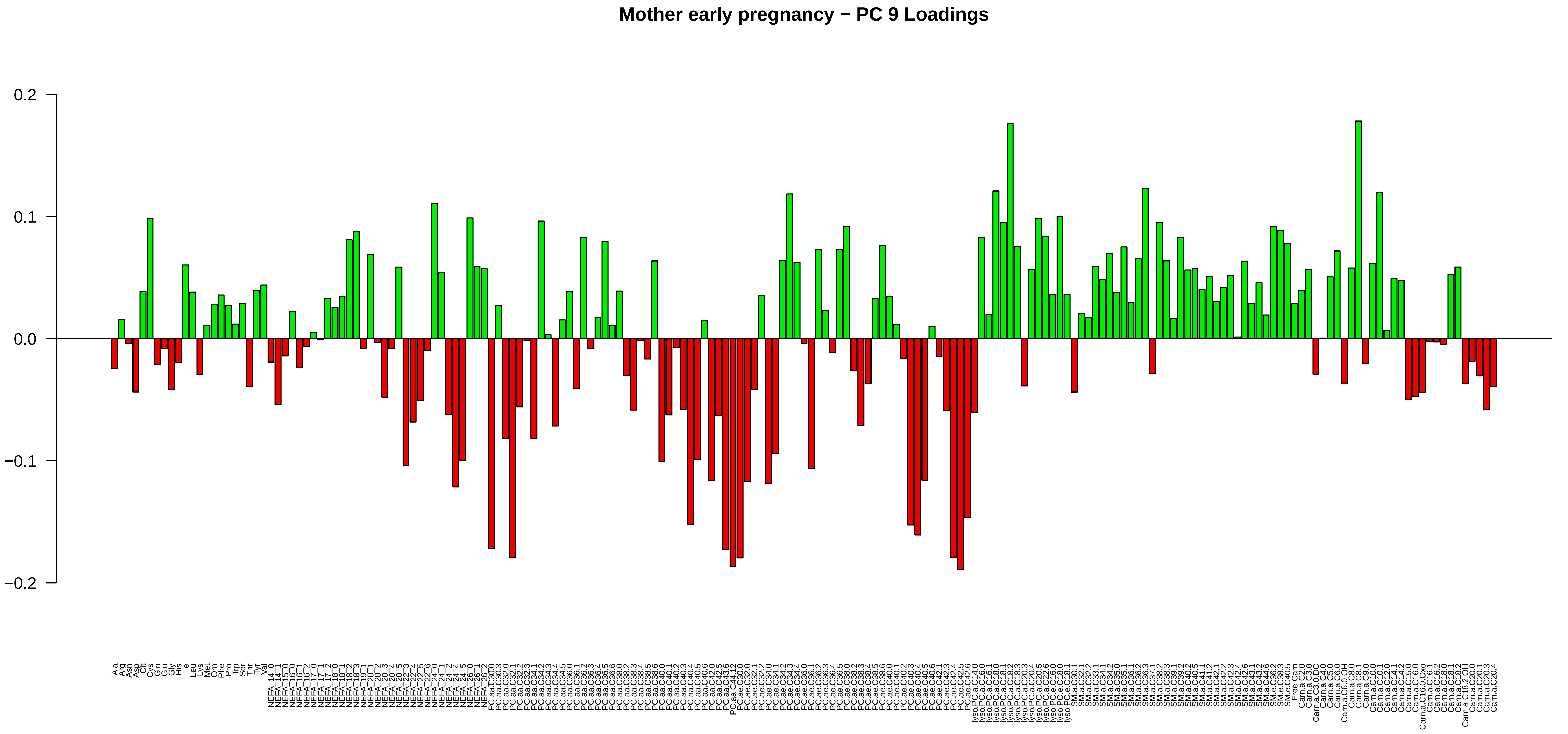
Mother early pregnancy − PC 9 Loadings
0.2
0.1
0.0
−0.1
−0.2

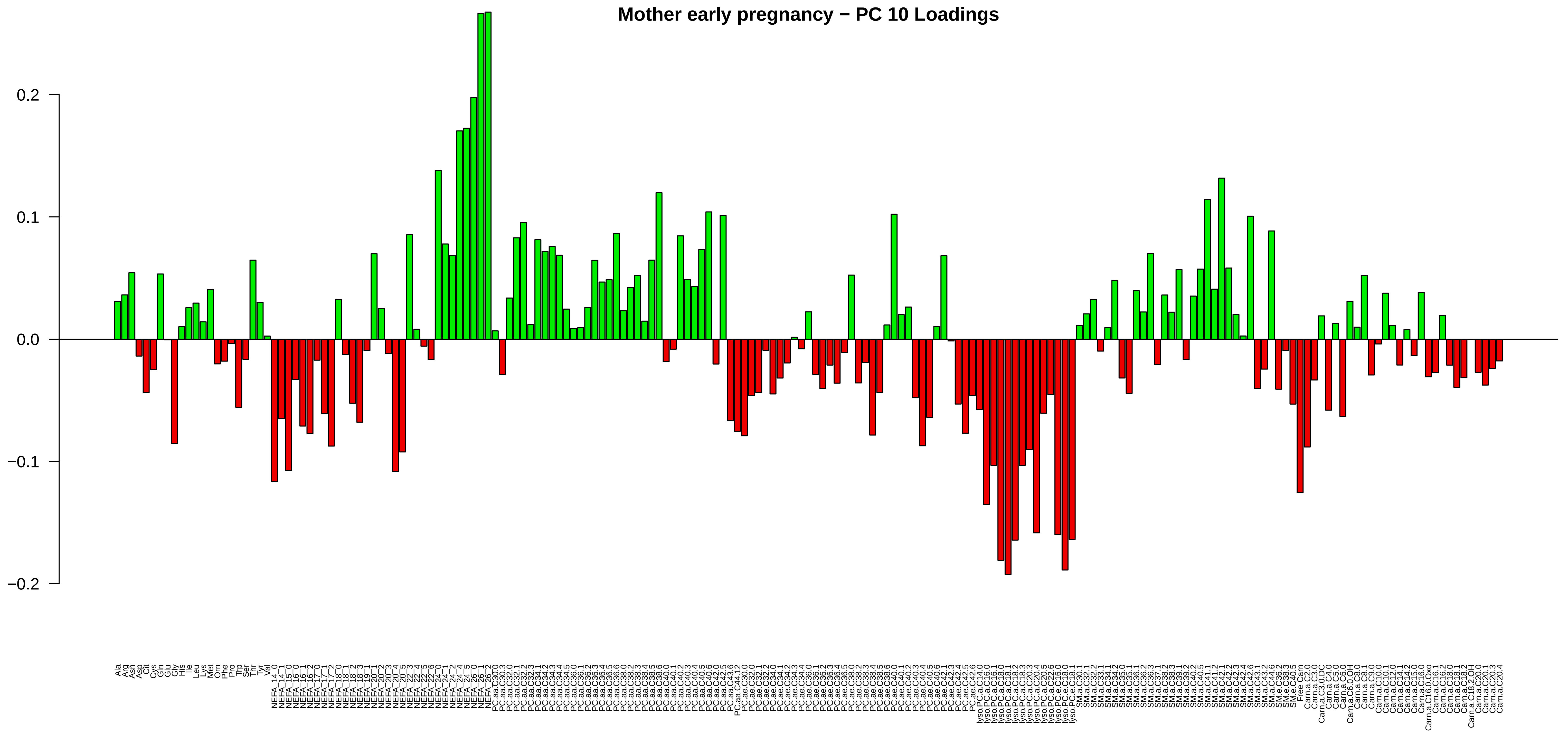
Mother early pregnancy − PC 10 Loadings
0.2
0.1
0.0
−0.1
−0.2

Mother early pregnancy − PC 11 Loadings

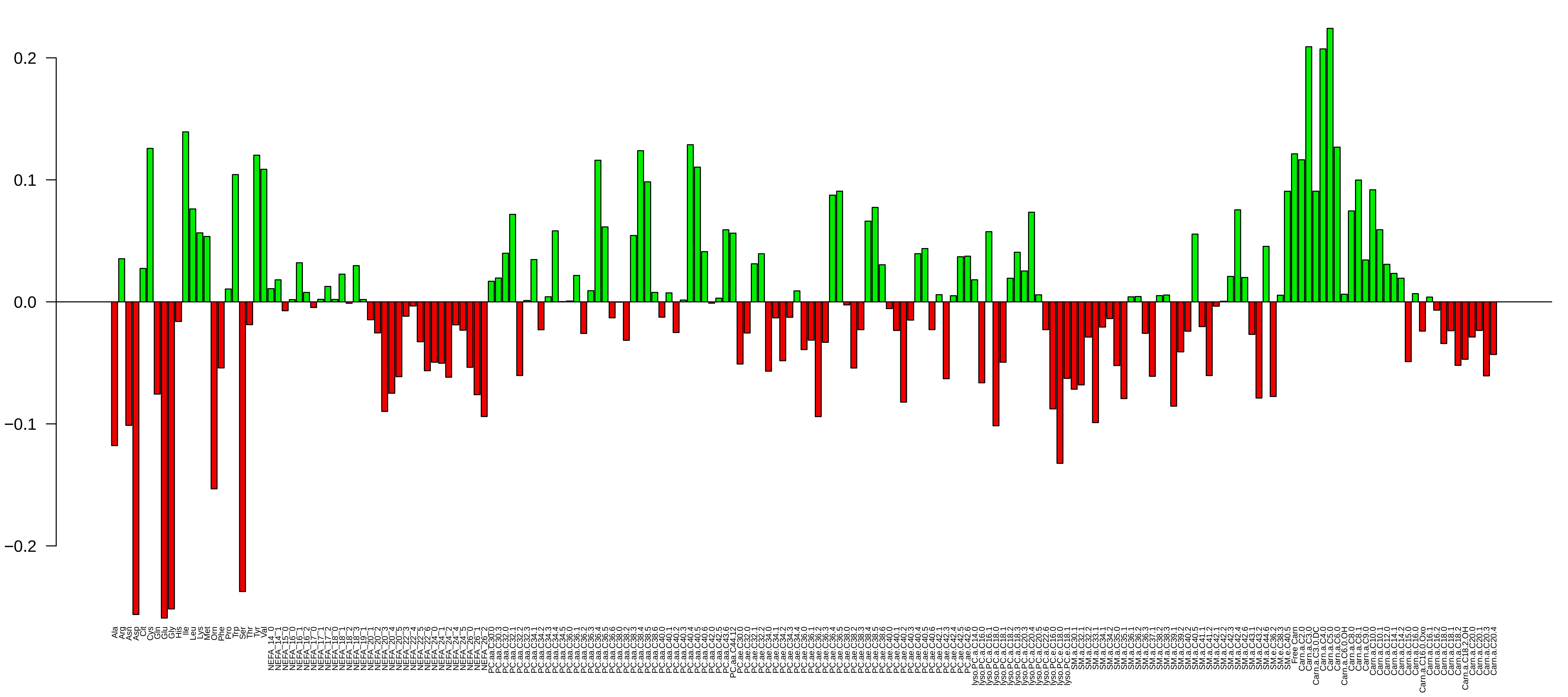

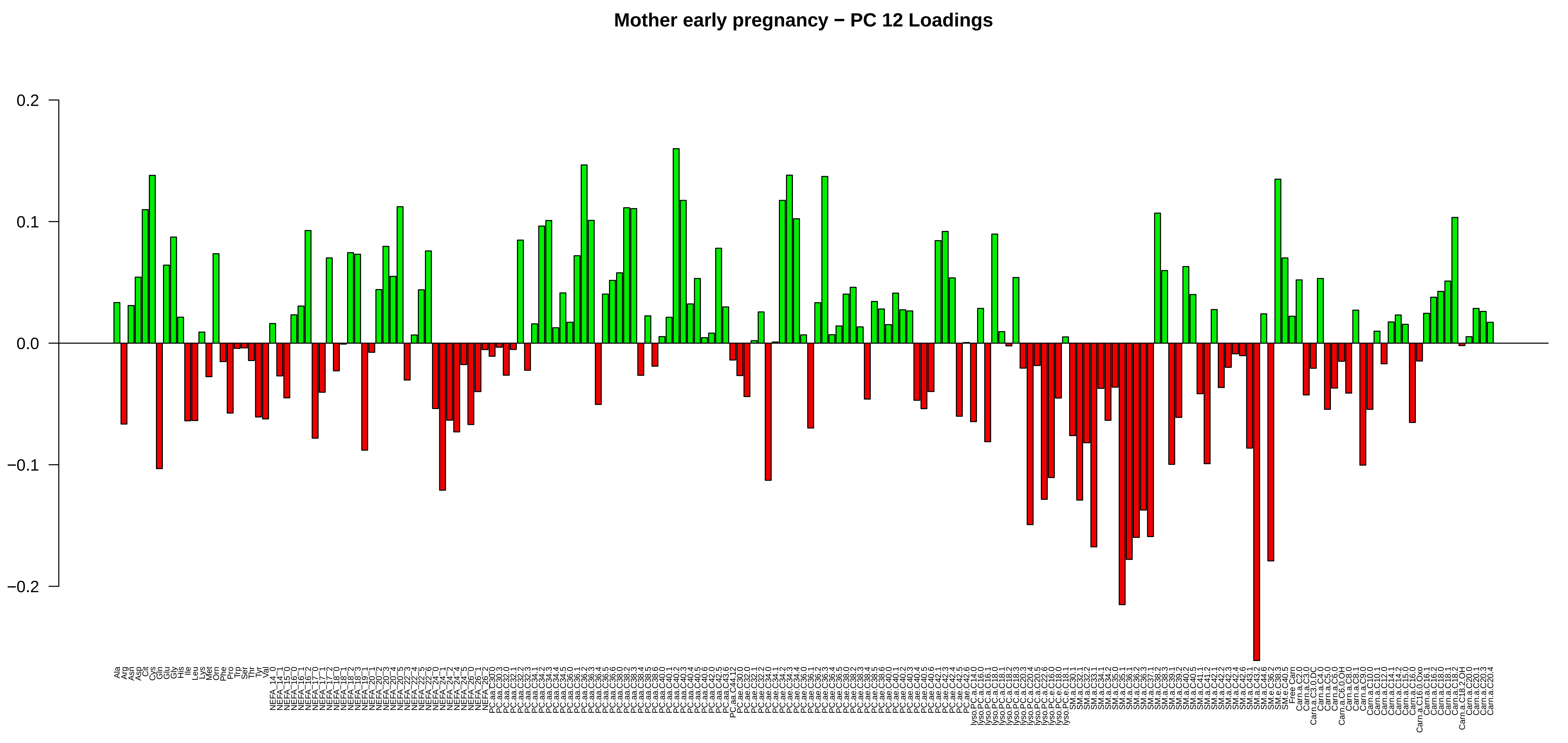
Mother early pregnancy − PC 12 Loadings
0.2
0.1
0.0
−0.1
−0.2
Ala
Arg
Asn
Asp
Cit
Cys
Gln
Glu
Gly
His
Ile
Leu
Lys
Met
Orn
Phe
Pro
Trp
Ser
Thr
Tyr
Val
NEFA_14_0
NEFA_14_1
NEFA_15_0
NEFA_16_0
NEFA_16_1
NEFA_16_2
NEFA_17_0
NEFA_17_1
NEFA_17_2
NEFA_18_0
NEFA_18_1
NEFA_18_2
NEFA_18_3
NEFA_19_1
NEFA_20_1
NEFA_20_2
NEFA_20_3
NEFA_20_4
NEFA_20_5
NEFA_22_3
NEFA_22_4
NEFA_22_5
NEFA_22_6
NEFA_24_0
NEFA_24_1
NEFA_24_2
NEFA_24_4
NEFA_24_5
NEFA_26_0
NEFA_26_1
NEFA_26_2
PC.aa.C30.0
PC.aa.C30.3
PC.aa.C32.0
PC.aa.C32.1
PC.aa.C32.2
PC.aa.C32.3
PC.aa.C34.1
PC.aa.C34.2
PC.aa.C34.3
PC.aa.C34.4
PC.aa.C34.5
PC.aa.C36.0
PC.aa.C36.1
PC.aa.C36.2
PC.aa.C36.3
PC.aa.C36.4
PC.aa.C36.5
PC.aa.C36.6
PC.aa.C38.0
PC.aa.C38.2
PC.aa.C38.3
PC.aa.C38.4
PC.aa.C38.5
PC.aa.C38.6
PC.aa.C40.0
PC.aa.C40.1
PC.aa.C40.2
PC.aa.C40.3
PC.aa.C40.4
PC.aa.C40.5
PC.aa.C40.6
PC.aa.C42.0
PC.aa.C42.5
PC.aa.C43.6
PC.aa.C44.12
PC.ae.C30.0
PC.ae.C32.0
PC.ae.C32.1
PC.ae.C32.2
PC.ae.C34.0
PC.ae.C34.1
PC.ae.C34.2
PC.ae.C34.3
PC.ae.C34.4
PC.ae.C36.0
PC.ae.C36.1
PC.ae.C36.2
PC.ae.C36.3
PC.ae.C36.4
PC.ae.C36.5
PC.ae.C38.0
PC.ae.C38.2
PC.ae.C38.3
PC.ae.C38.4
PC.ae.C38.5
PC.ae.C38.6
PC.ae.C40.0
PC.ae.C40.1
PC.ae.C40.2
PC.ae.C40.3
PC.ae.C40.4
PC.ae.C40.5
PC.ae.C40.6
PC.ae.C42.1
PC.ae.C42.3
PC.ae.C42.4
PC.ae.C42.5
PC.ae.C42.6
lyso.PC.a.C14.0
lyso.PC.a.C16.0
lyso.PC.a.C16.1
lyso.PC.a.C18.0
lyso.PC.a.C18.1
lyso.PC.a.C18.2
lyso.PC.a.C18.3
lyso.PC.a.C20.3
lyso.PC.a.C20.4
lyso.PC.a.C20.5
lyso.PC.a.C22.6
lyso.PC.e.C16.0
lyso.PC.e.C18.0
lyso.PC.e.C18.1
SM.a.C30.1
SM.a.C32.1
SM.a.C32.2
SM.a.C33.1
SM.a.C34.1
SM.a.C34.2
SM.a.C35.0
SM.a.C35.1
SM.a.C36.1
SM.a.C36.2
SM.a.C36.3
SM.a.C37.1
SM.a.C38.2
SM.a.C38.3
SM.a.C39.1
SM.a.C39.2
SM.a.C40.2
SM.a.C40.5
SM.a.C41.1
SM.a.C41.2
SM.a.C42.1
SM.a.C42.2
SM.a.C42.3
SM.a.C42.4
SM.a.C42.6
SM.a.C43.1
SM.a.C43.2
SM.a.C44.6
SM.e.C36.2
SM.e.C38.3
SM.e.C40.5
Free Carn
Carn.a.C2.0
Carn.a.C3.0
Carn.a.C3.0.DC
Carn.a.C4.0
Carn.a.C5.0
Carn.a.C6.0
Carn.a.C6.0.OH
Carn.a.C8.0
Carn.a.C8.1
Carn.a.C9.0
Carn.a.C10.0
Carn.a.C10.1
Carn.a.C12.0
Carn.a.C14.1
Carn.a.C14.2
Carn.a.C15.0
Carn.a.C16.0
Carn.a.C16.0.Oxo
Carn.a.C16.1
Carn.a.C16.2
Carn.a.C18.0
Carn.a.C18.1
Carn.a.C18.2
Carn.a.C18.2.OH
Carn.a.C20.0
Carn.a.C20.1
Carn.a.C20.3
Carn.a.C20.4

Mother early pregnancy − PC 13 Loadings

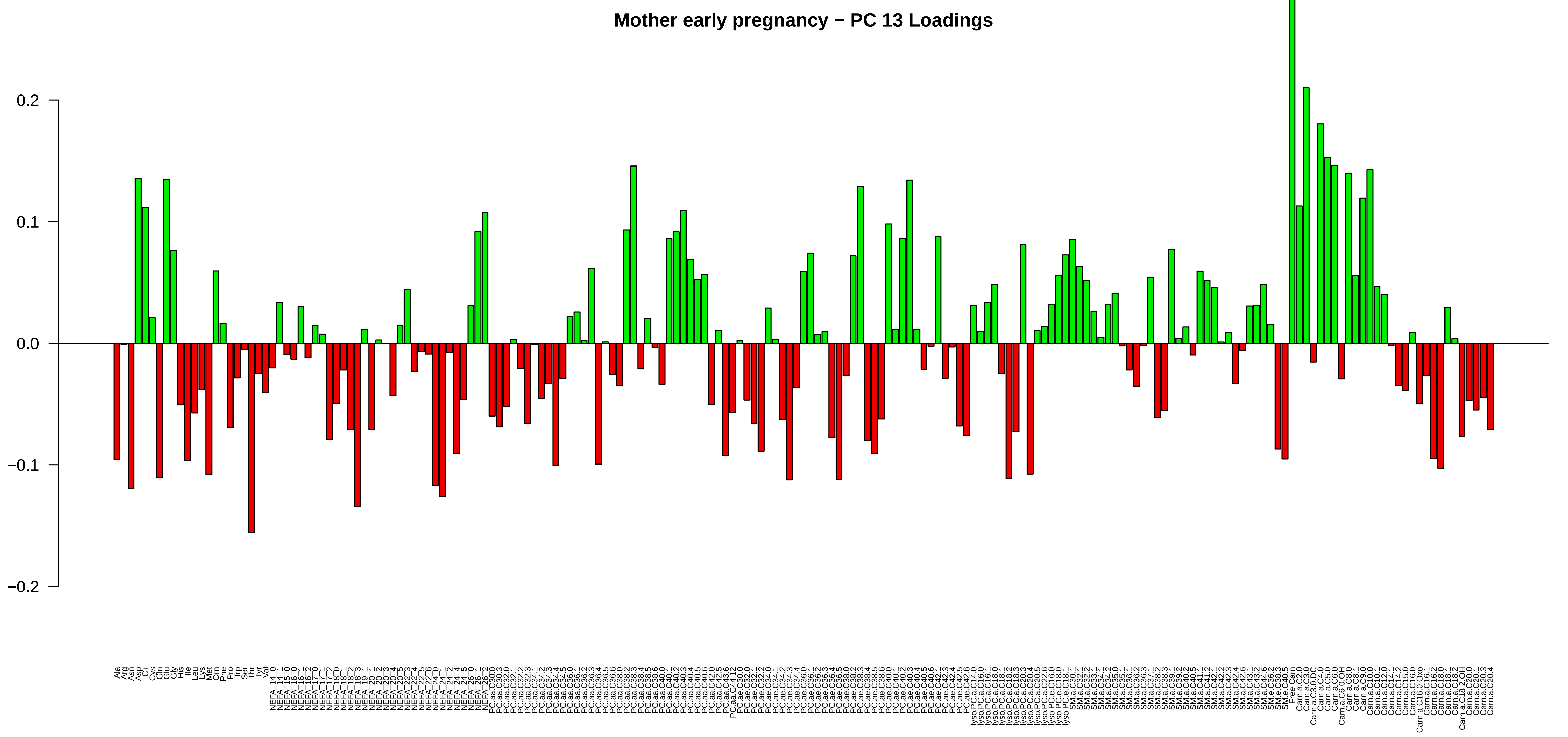

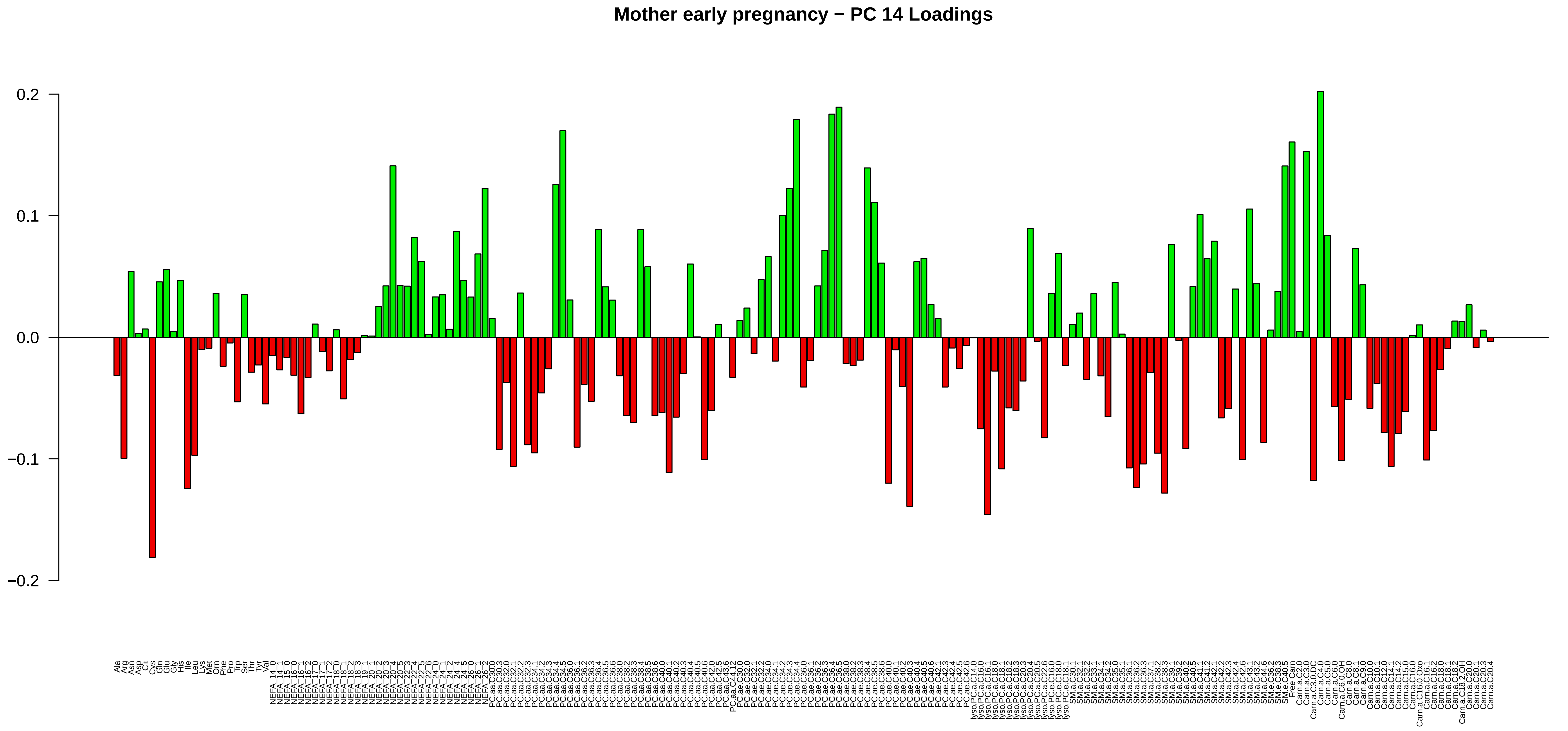
Mother early pregnancy − PC 14 Loadings
0.2
0.1
0.0
−0.1
−0.2

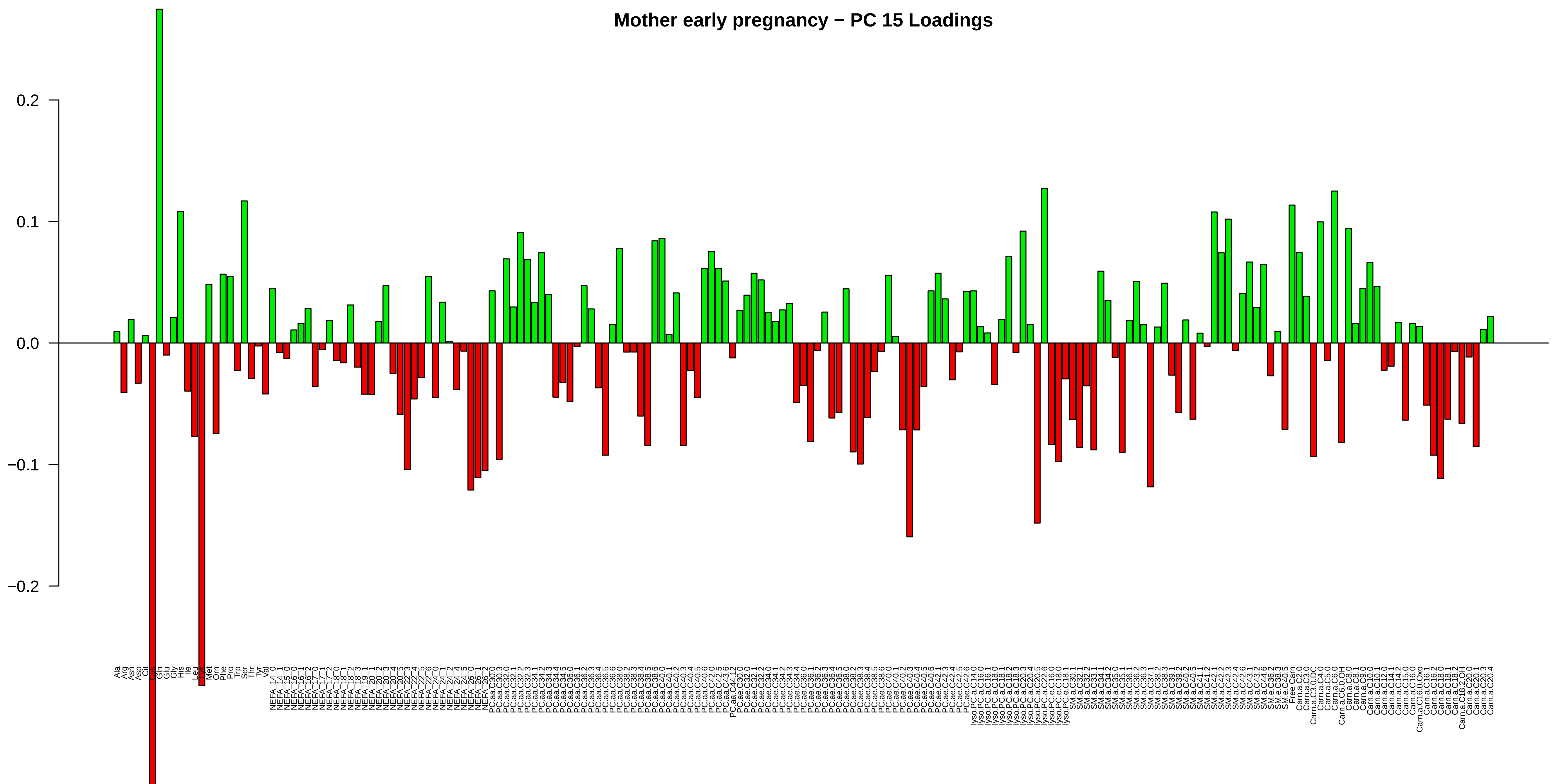
Mother early pregnancy − PC 15 Loadings
0.2
0.1
0.0
−0.1
−0.2

Mother early pregnancy − PC 16 Loadings

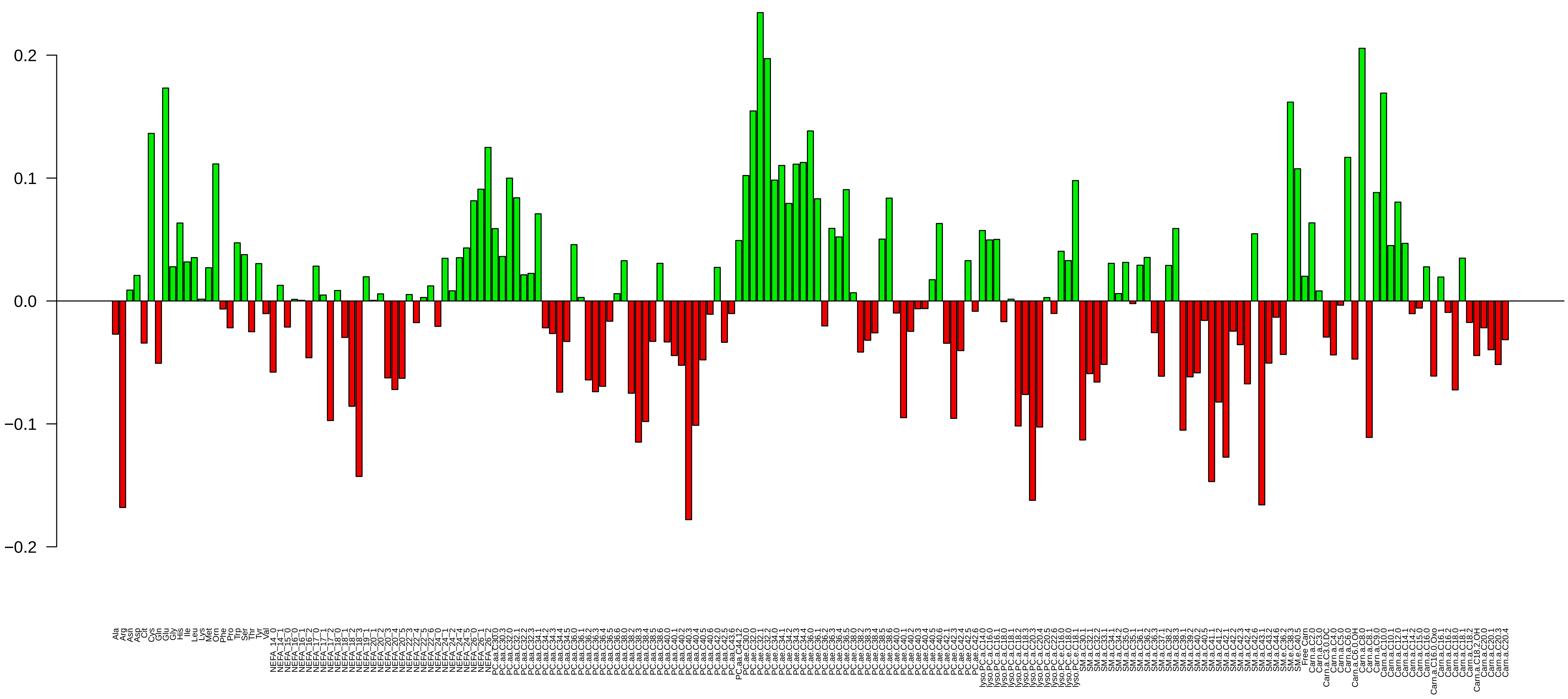

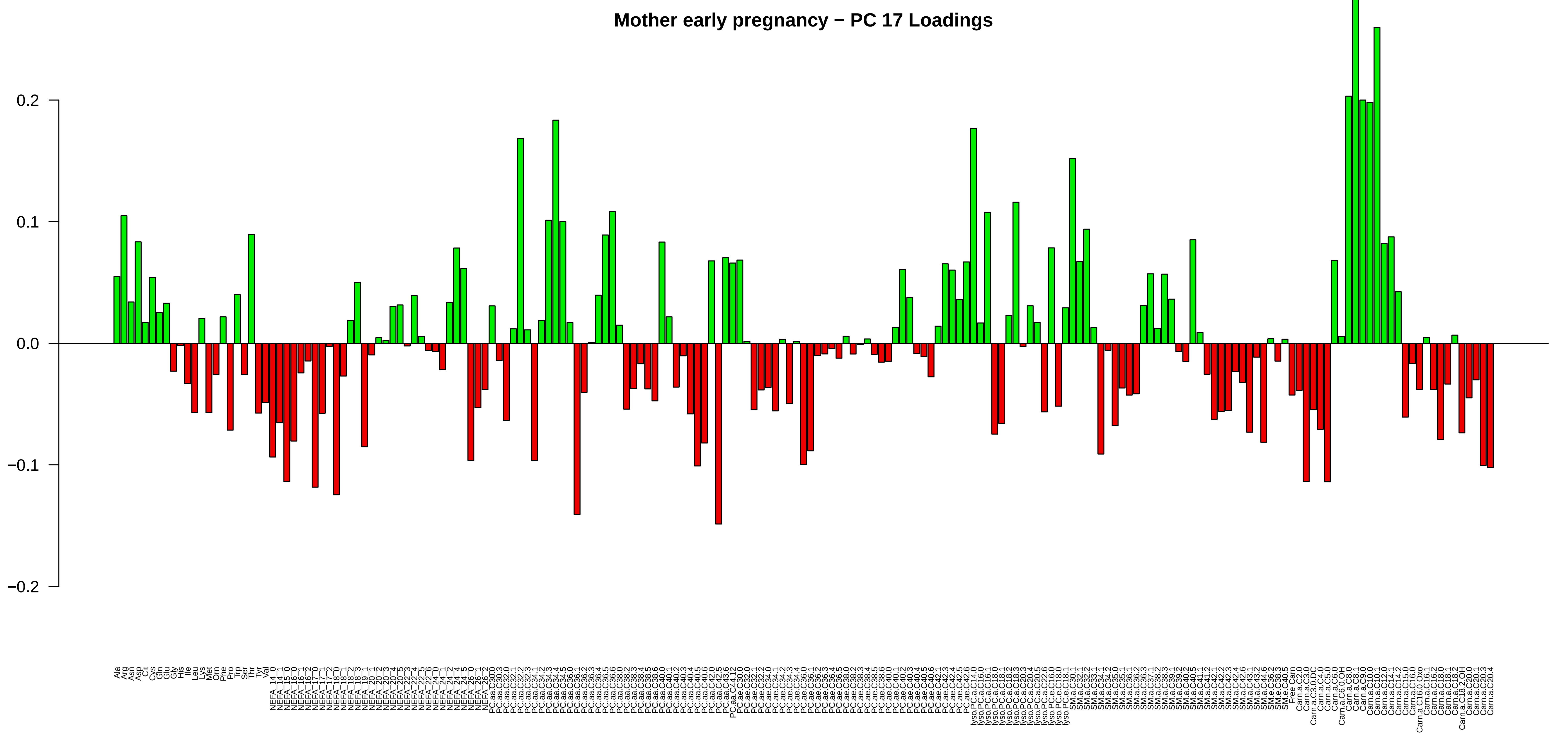
Mother early pregnancy − PC 17 Loadings
0.2
0.1
0.0
−0.1
−0.2

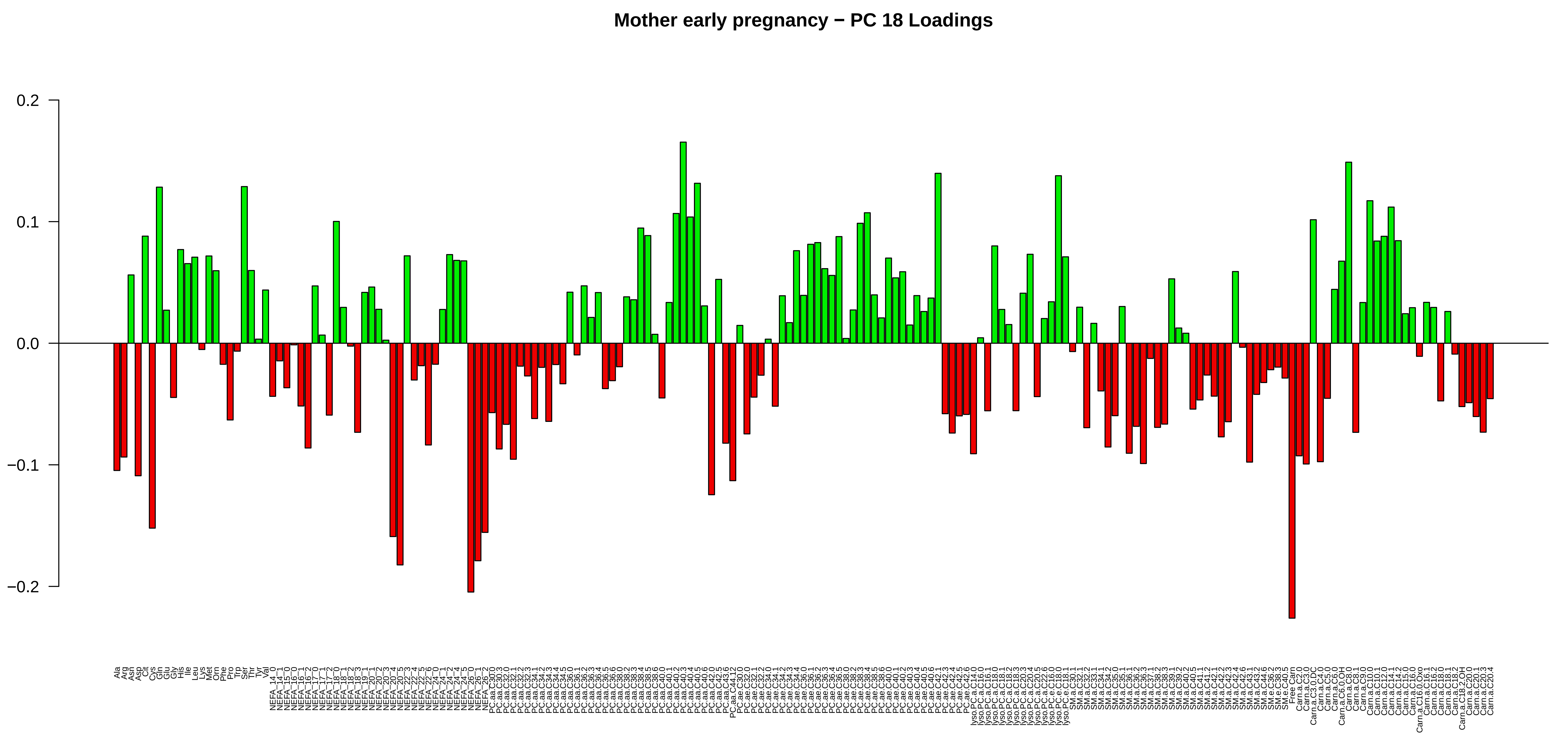
Mother early pregnancy − PC 18 Loadings
0.2
0.1
0.0
−0.1
−0.2

Mother early pregnancy − PC 19 Loadings

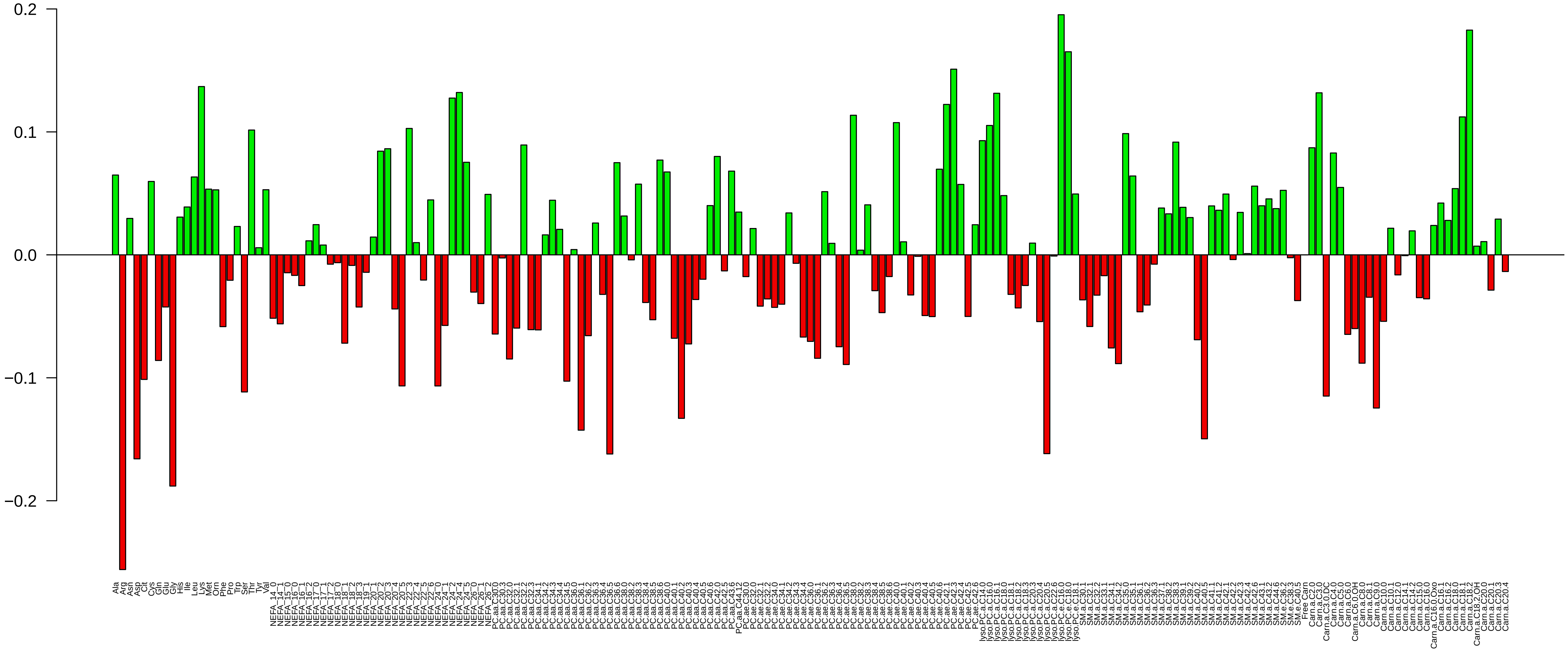

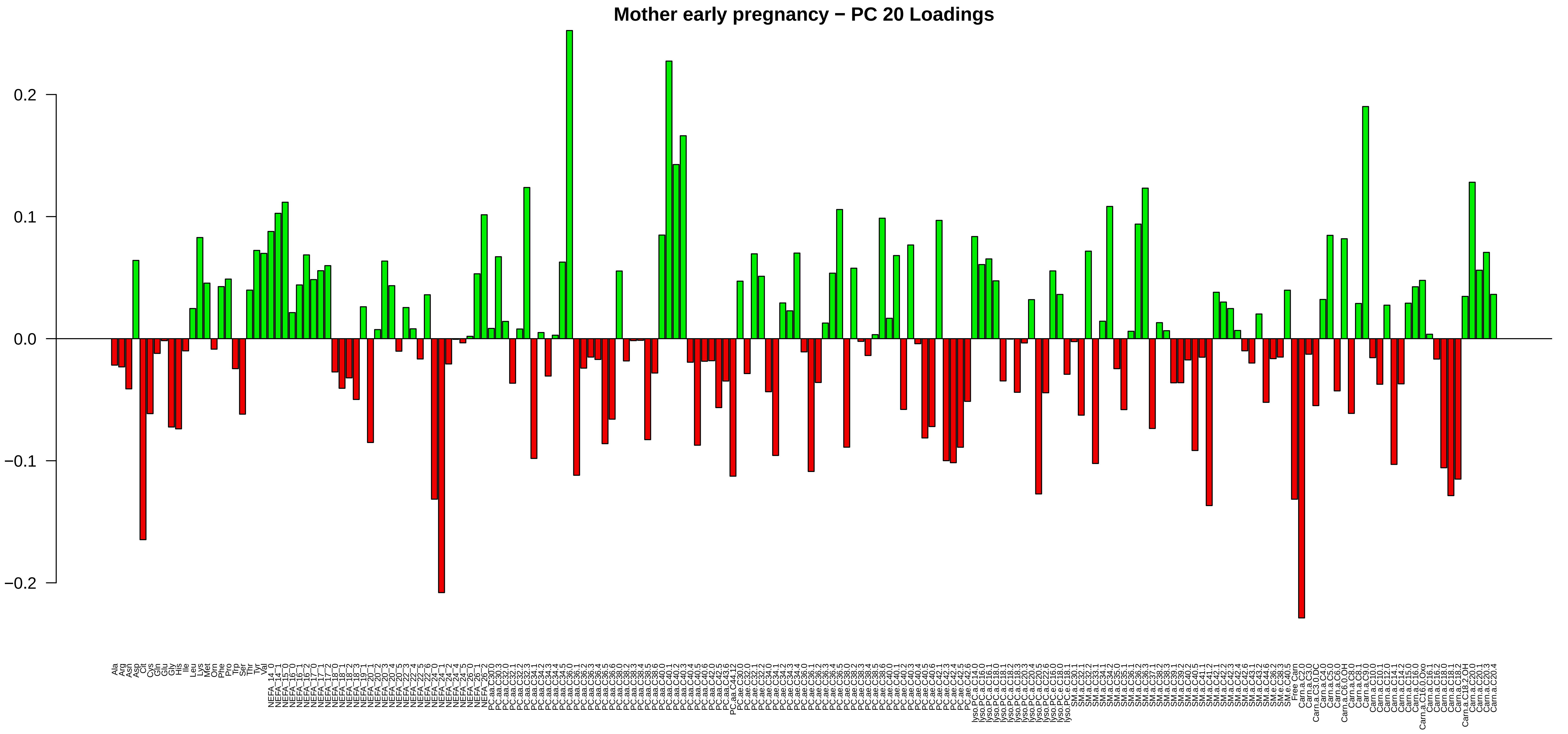
Mother early pregnancy − PC 20 Loadings
0.2
0.1
0.0
−0.1
−0.2

Mother early pregnancy − PC 21 Loadings

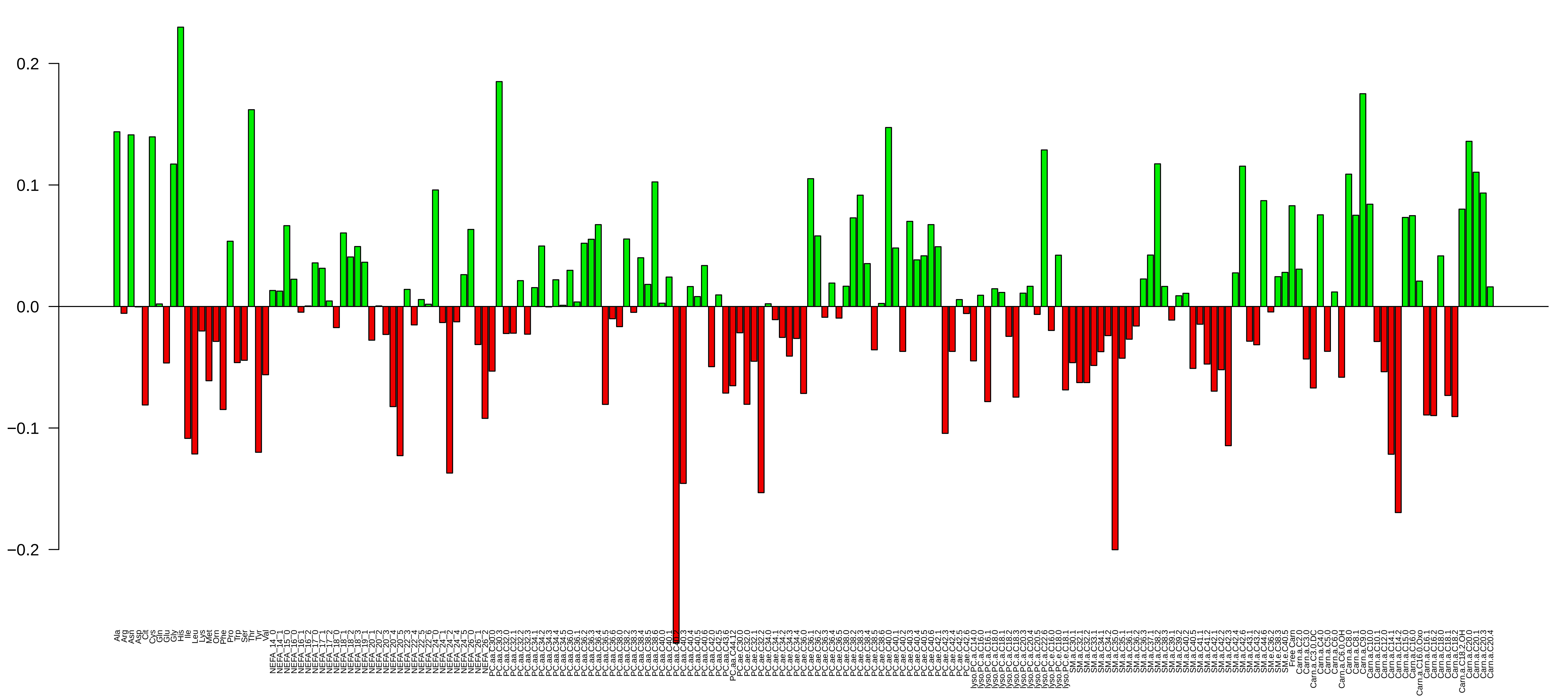

Mother early pregnancy − PC 22 Loadings

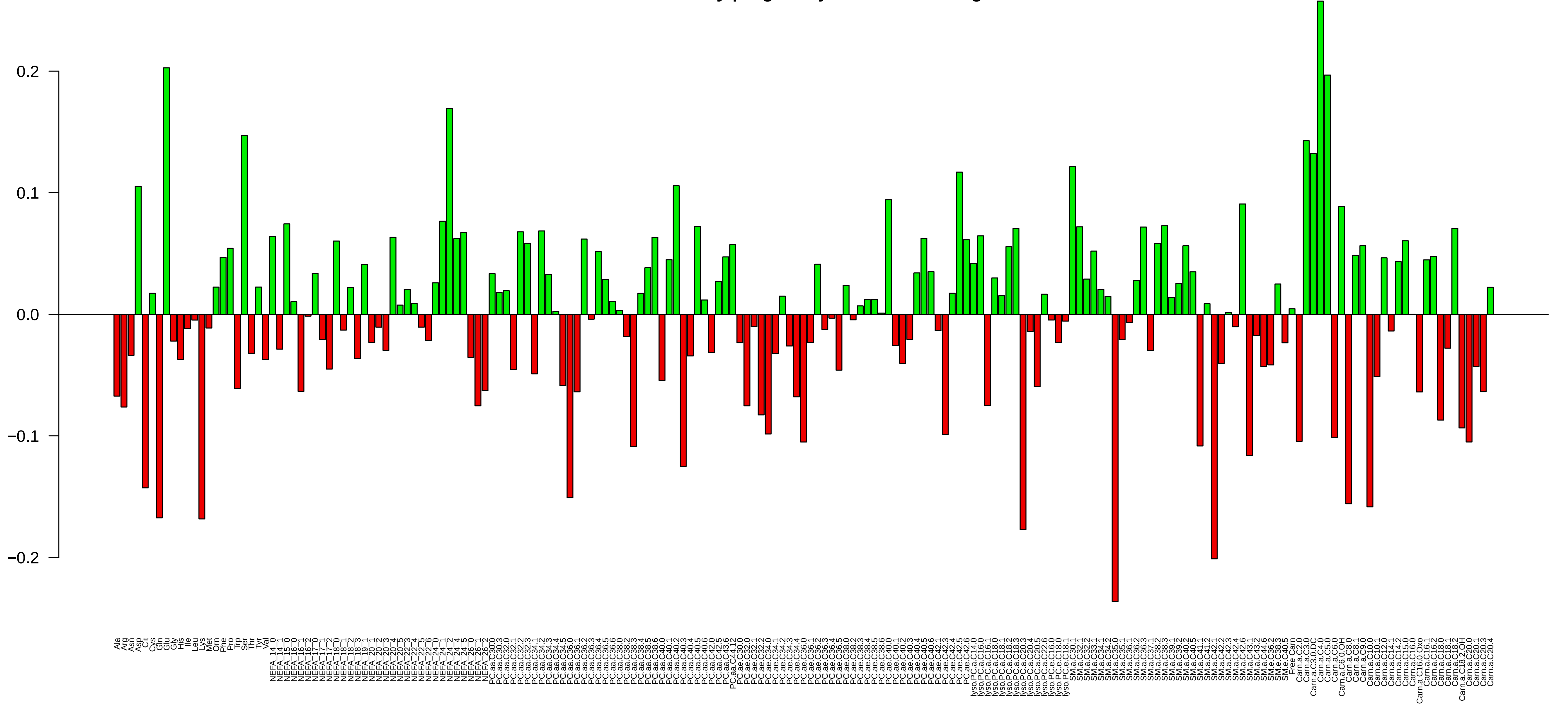

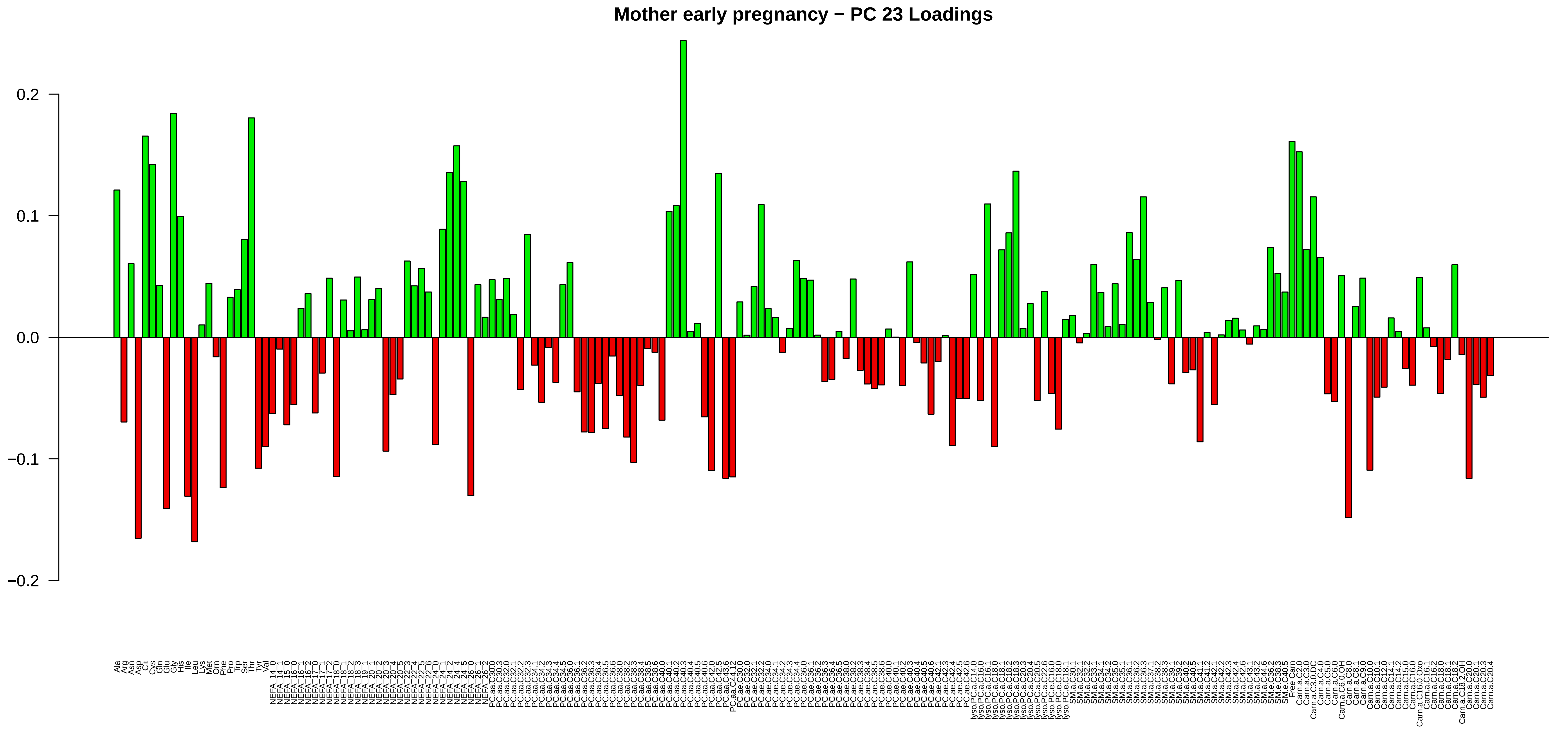

Mother early pregnancy − PC 23 Loadings
0.2
0.1
0.0
−0.1
−0.2

Mother early pregnancy − PC 24 Loadings

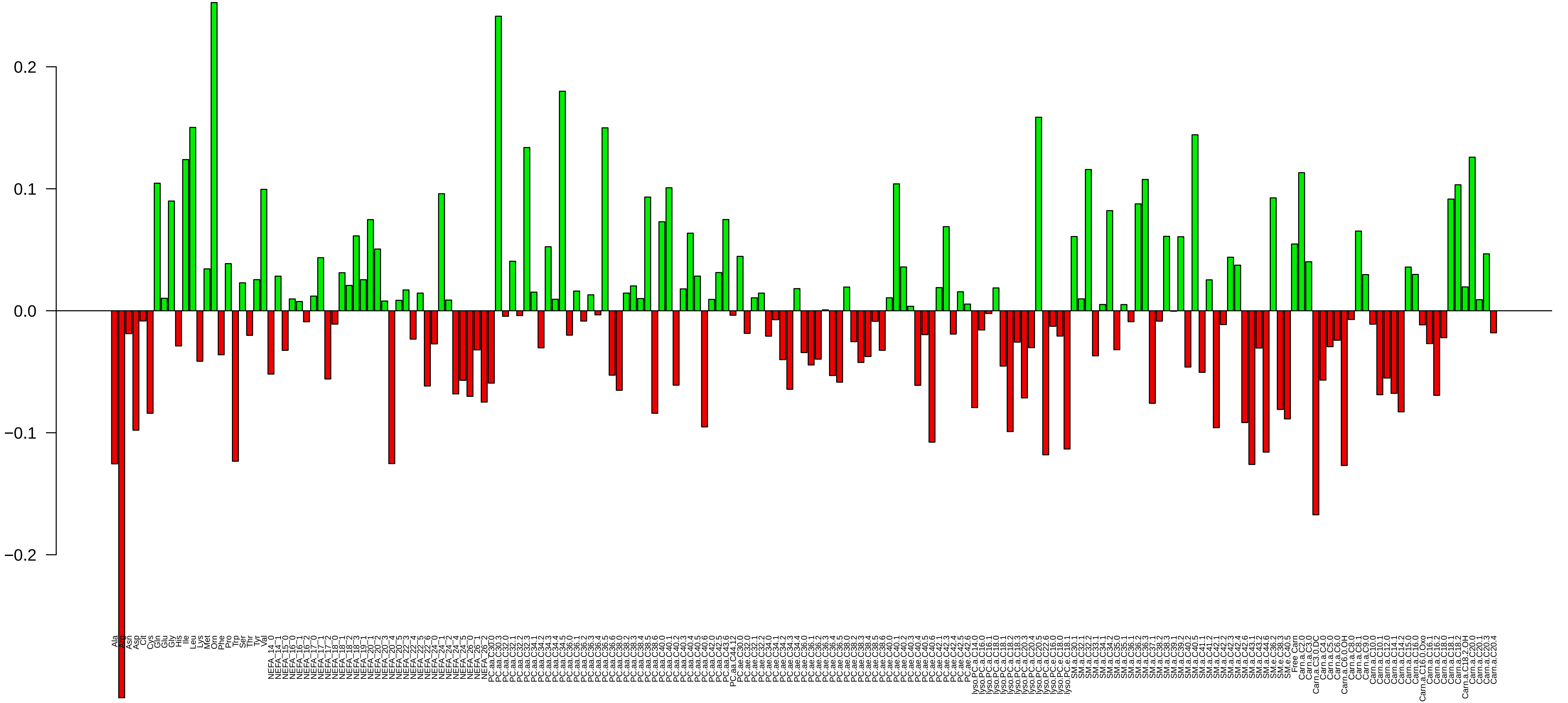

Mother early pregnancy − PC 25 Loadings

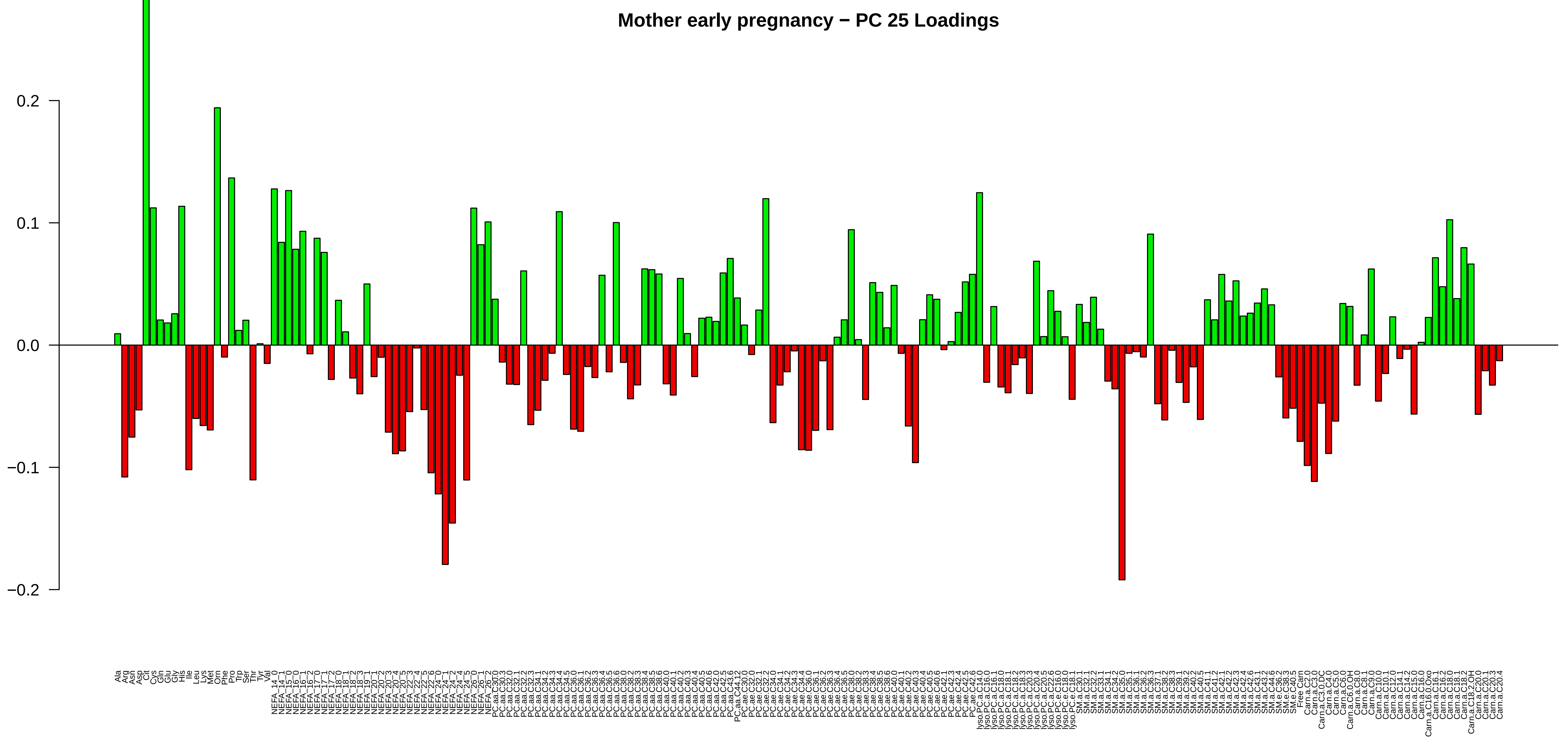

Supplement: Supplementary file 5 — Supplemental Figure S2 (PDF 102 kb) [file 11306_2020_1667_MOESM5_ESM.pdf]
