# Supplementary material for: A population-based resource for intergenerational metabolomics analyses in pregnant women and their children: the Generation R Study
Source: Metabolomics. 2020 Mar 23;16(4):43. doi: 10.1007/s11306-020-01667-1 (PMC7089886; doi:10.1007/s11306-020-01667-1)

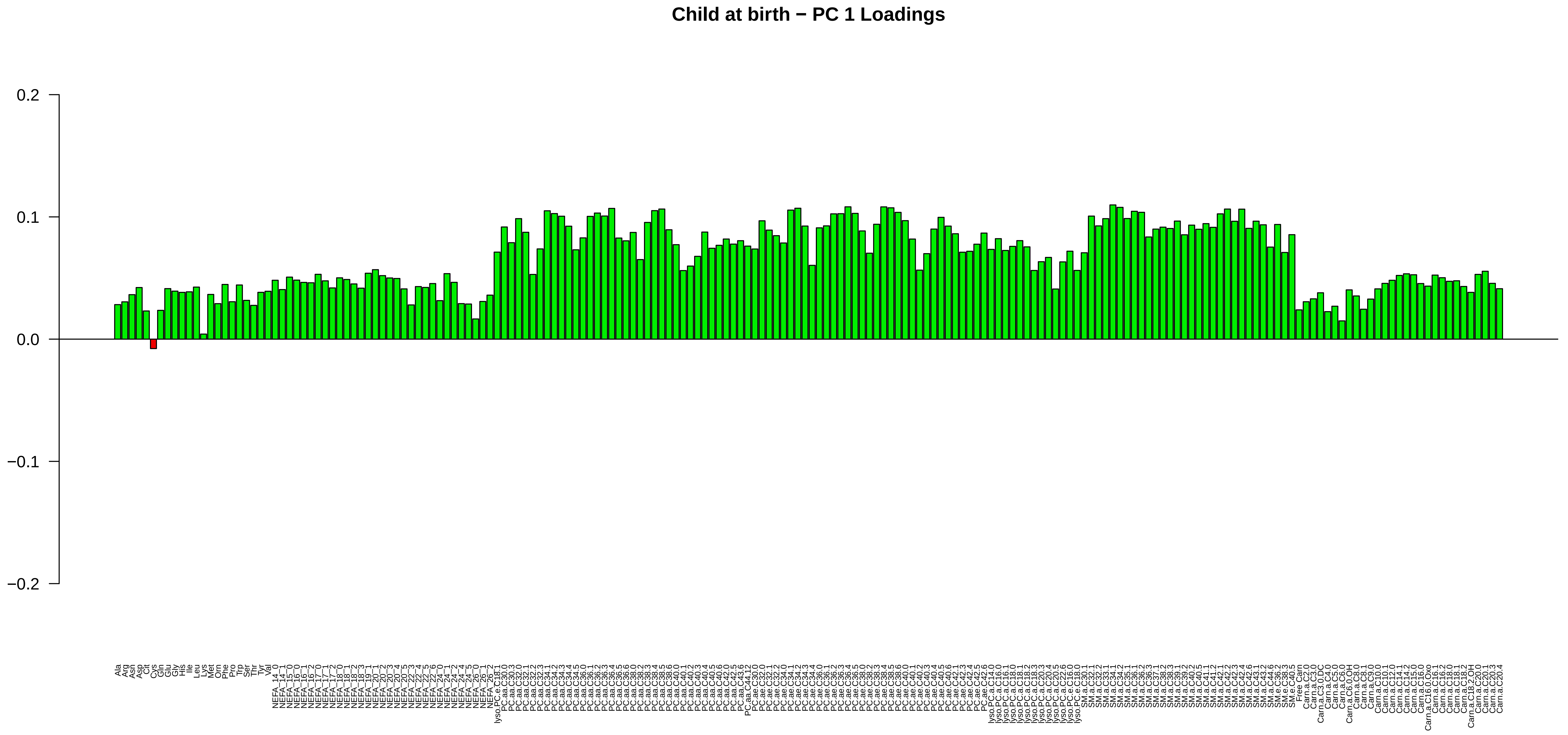
Child at birth − PC 1 Loadings
0.2
0.1
0.0
−0.1
−0.2

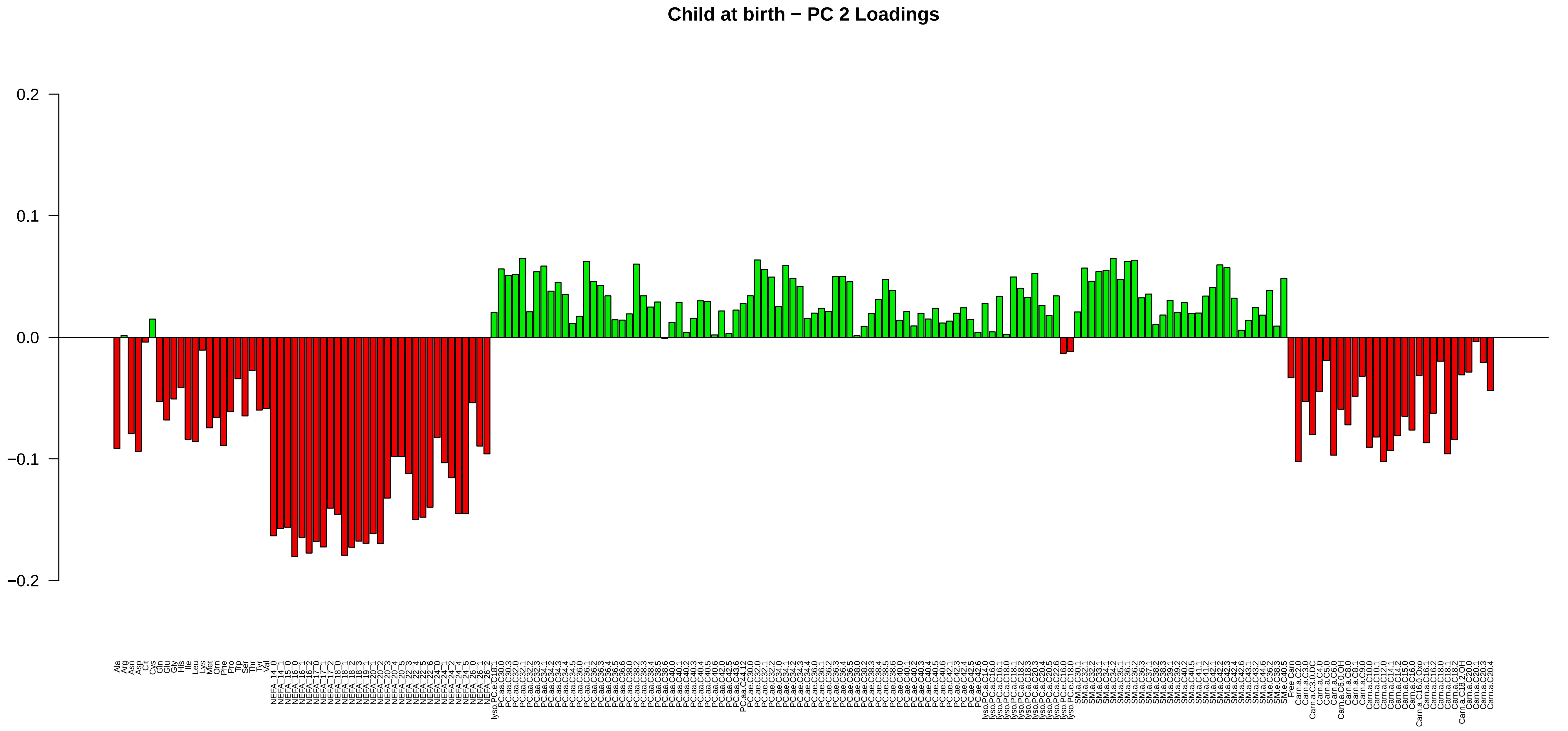
Child at birth − PC 2 Loadings
0.2
0.1
0.0
−0.1
−0.2

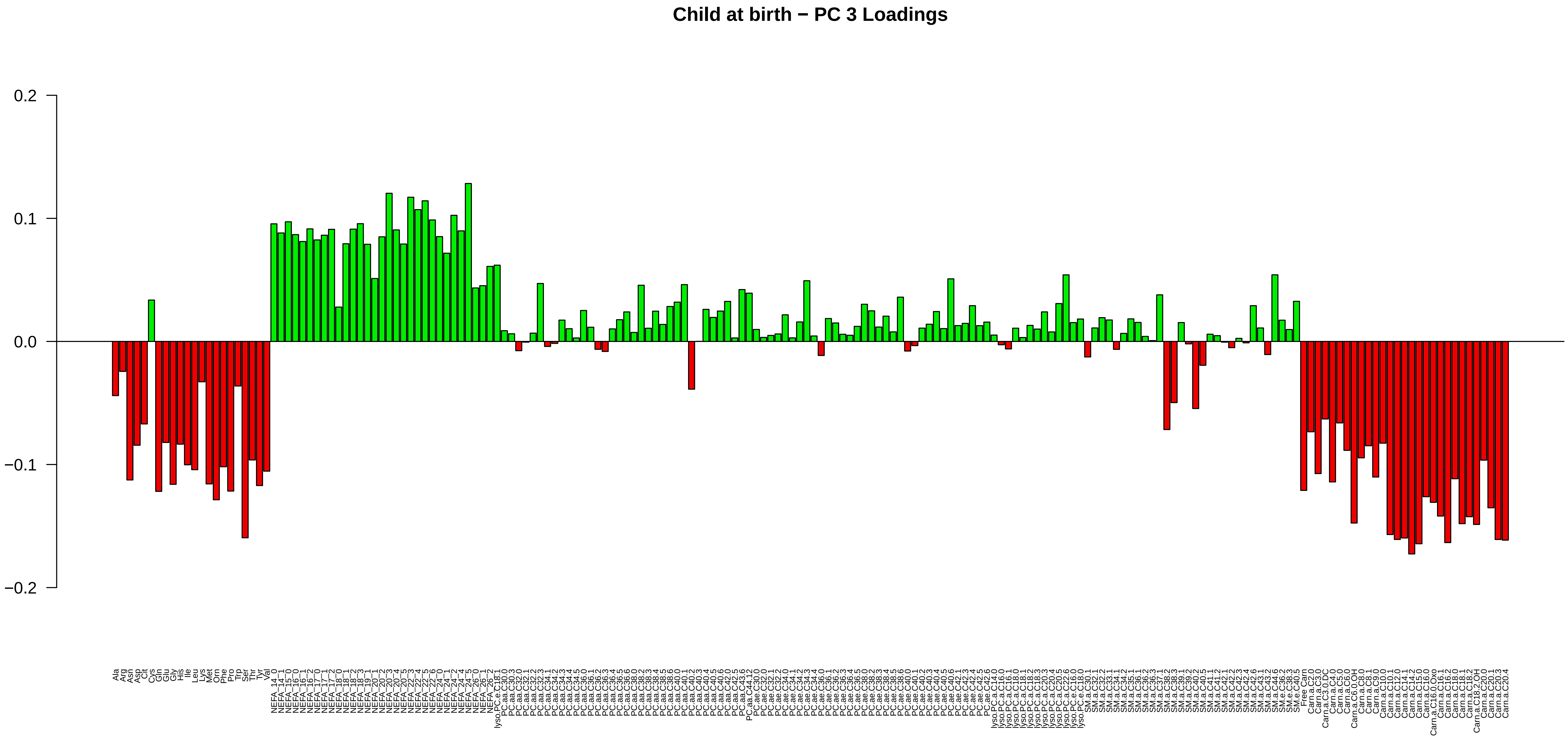
Child at birth − PC 3 Loadings
0.2
0.1
0.0
−0.1
−0.2

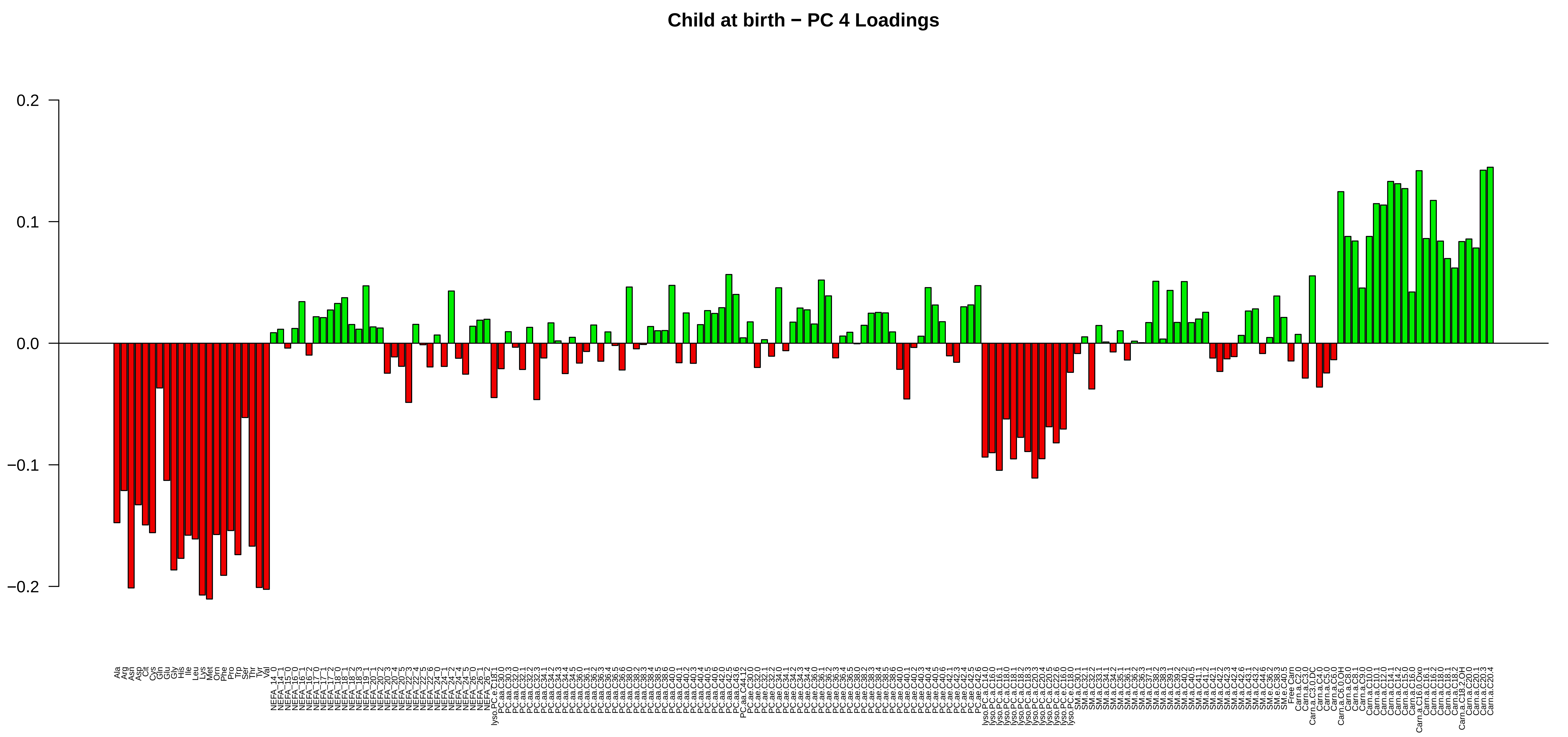
Child at birth − PC 4 Loadings
0.2
0.1
0.0
−0.1
−0.2

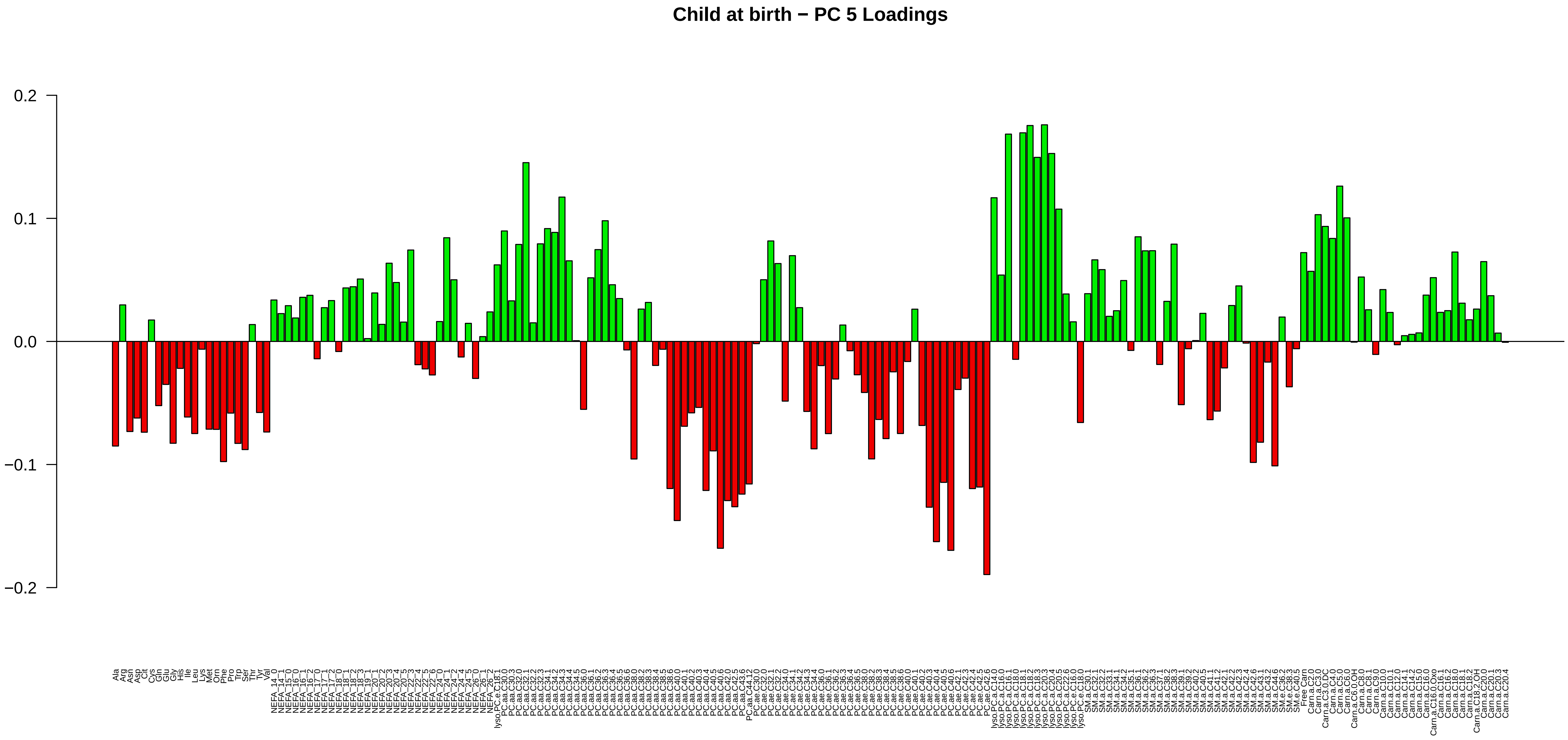
Child at birth − PC 5 Loadings
0.2
0.1
0.0
−0.1
−0.2

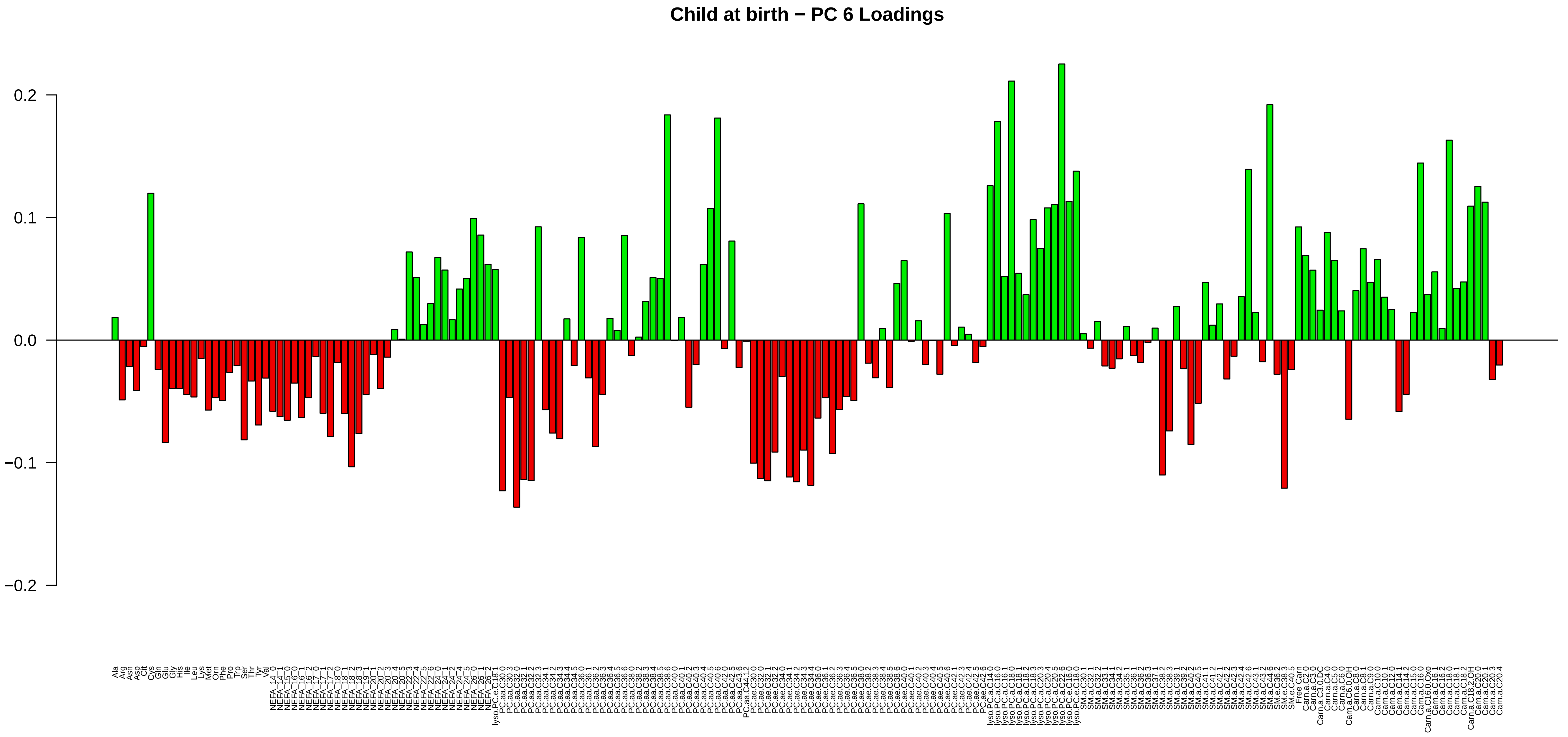
Child at birth − PC 6 Loadings
0.2
0.1
0.0
−0.1
−0.2

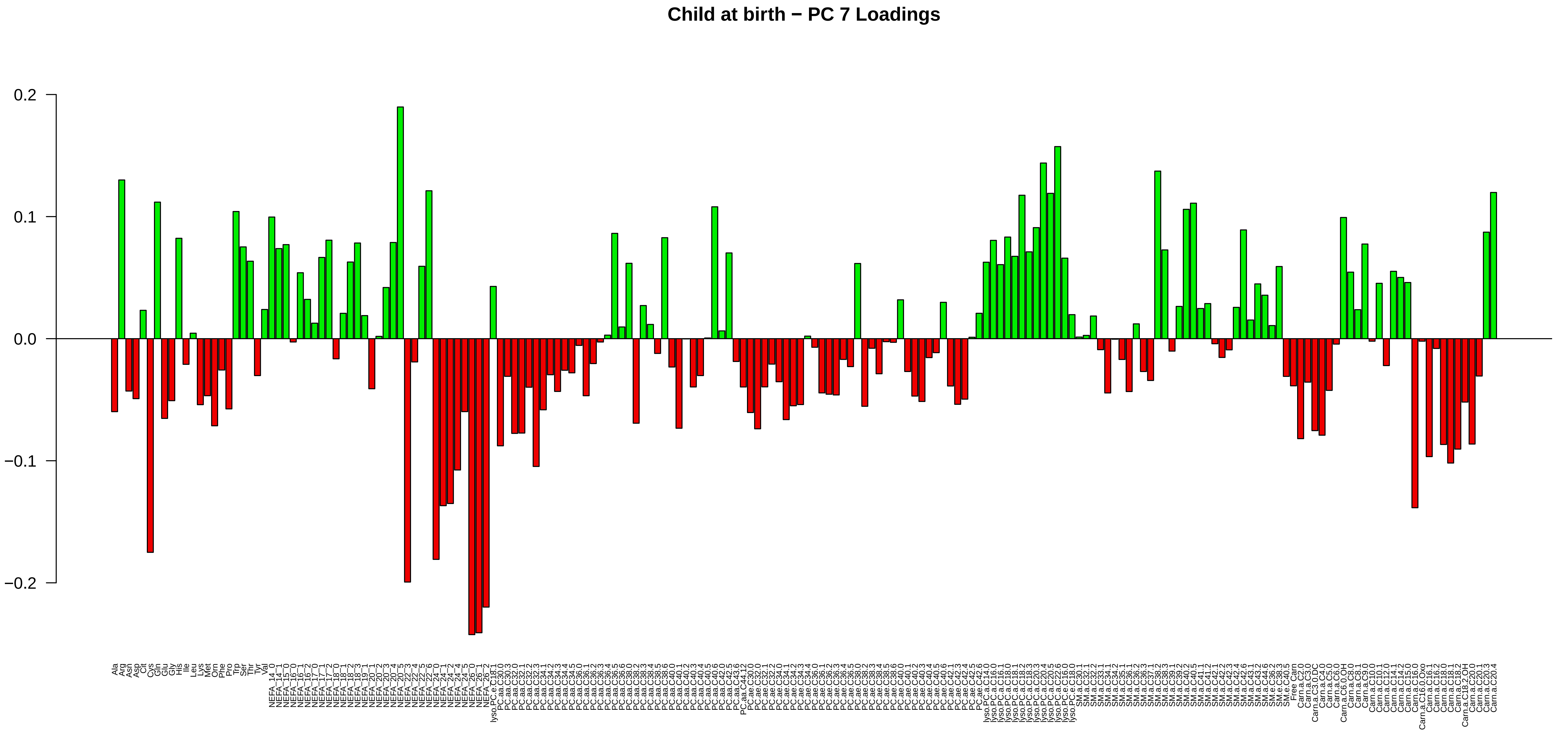
Child at birth − PC 7 Loadings
0.2
0.1
0.0
−0.1
−0.2

Child at birth − PC 8 Loadings

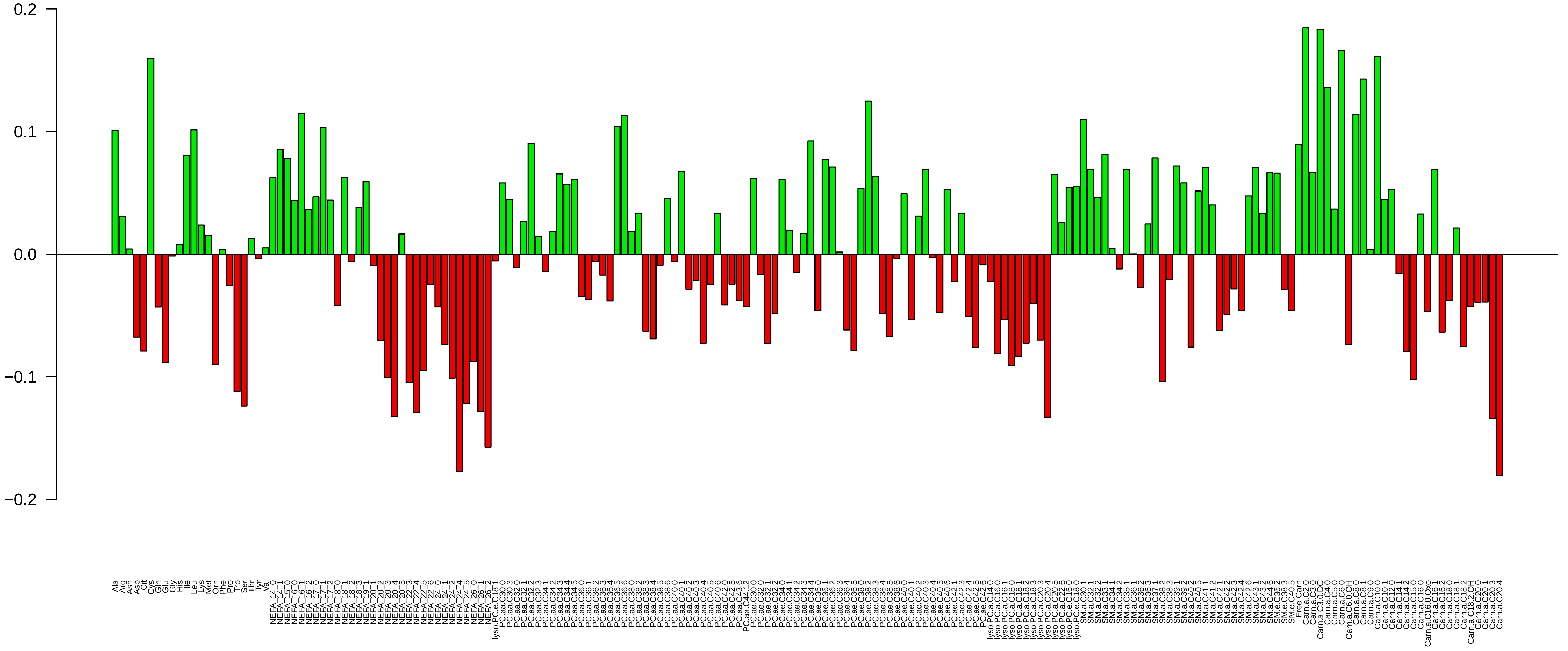

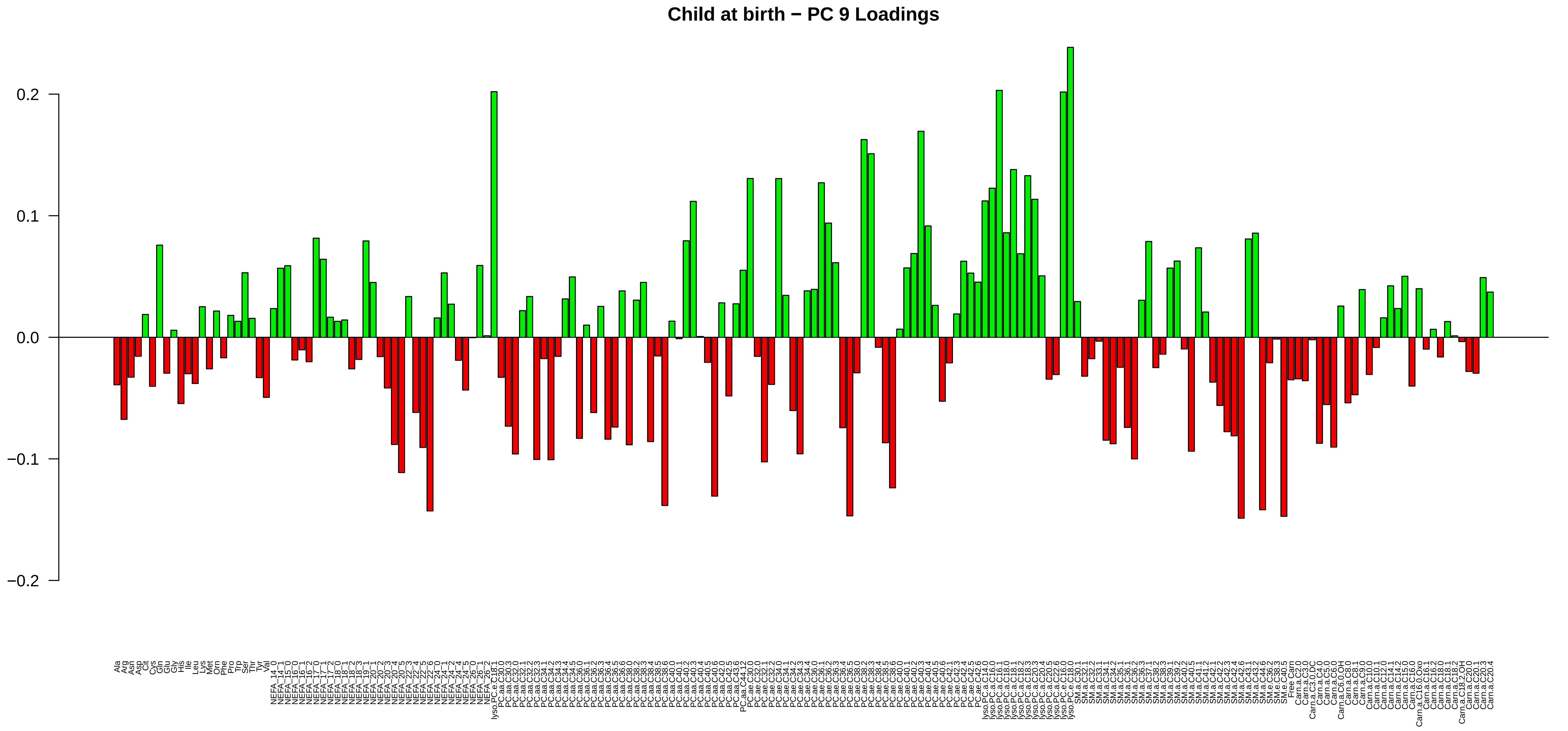
Child at birth − PC 9 Loadings
0.2
0.1
0.0
−0.1
−0.2

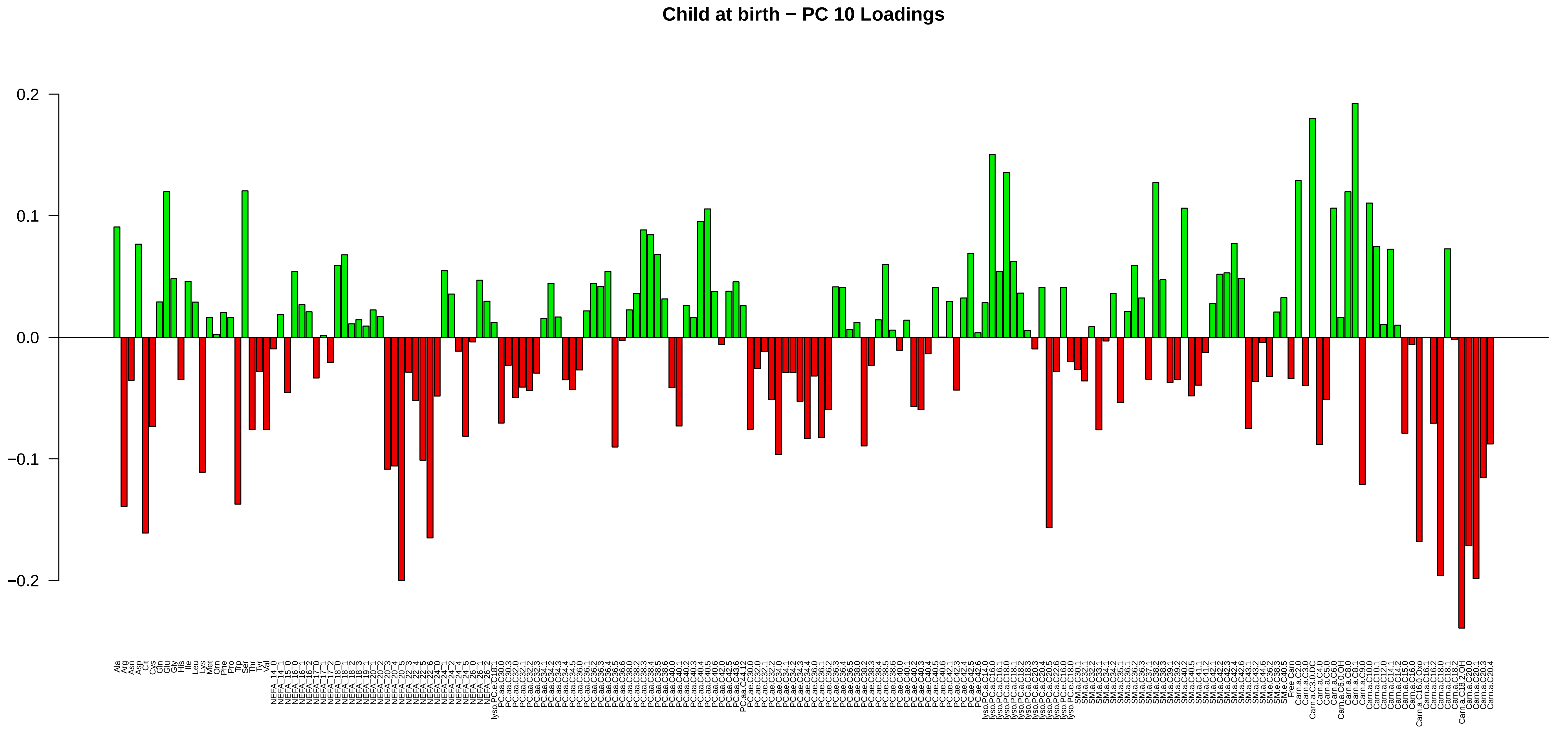
Child at birth − PC 10 Loadings
0.2
0.1
0.0
−0.1
−0.2

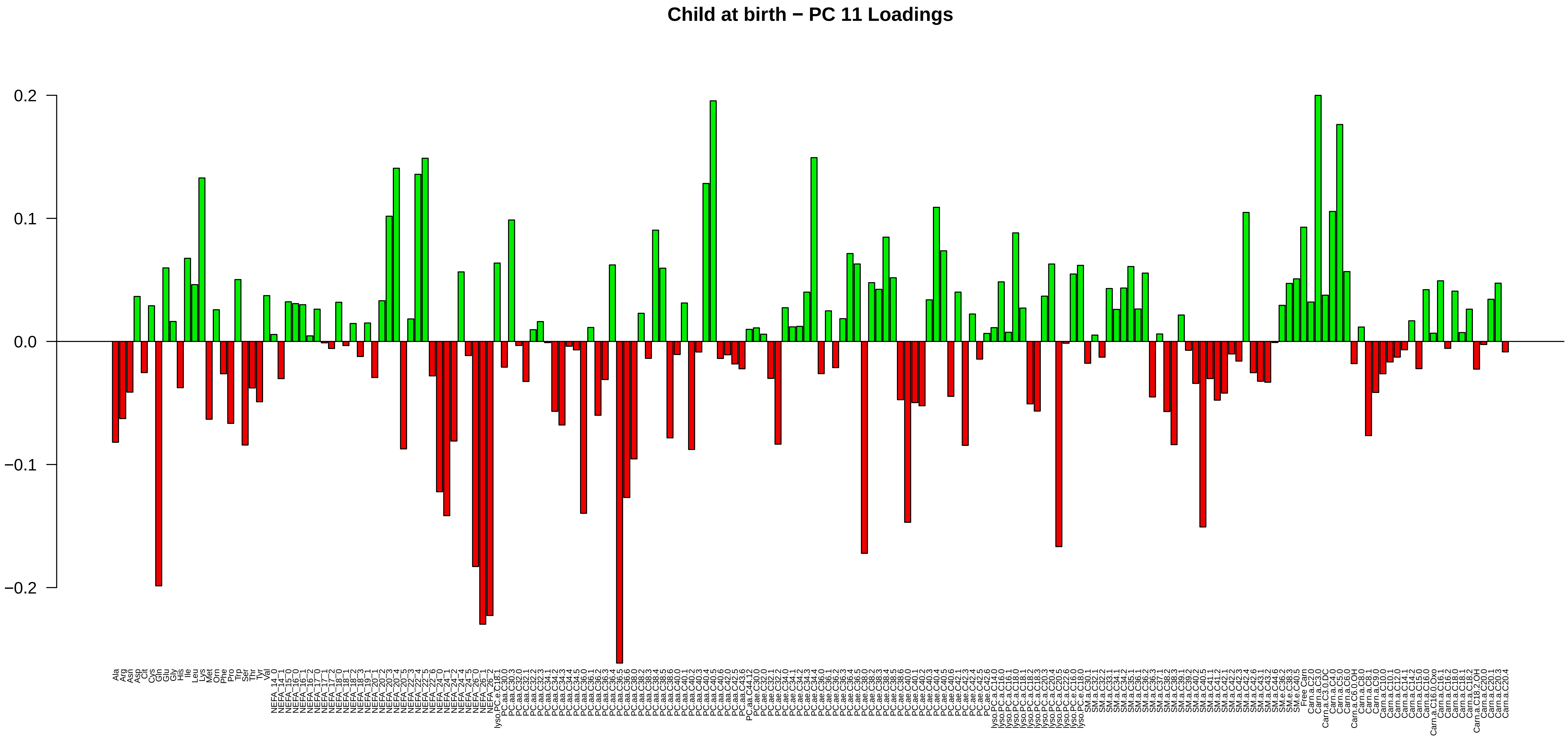

Child at birth − PC 11 Loadings
0.2
0.1
0.0
−0.1
−0.2

Child at birth − PC 12 Loadings

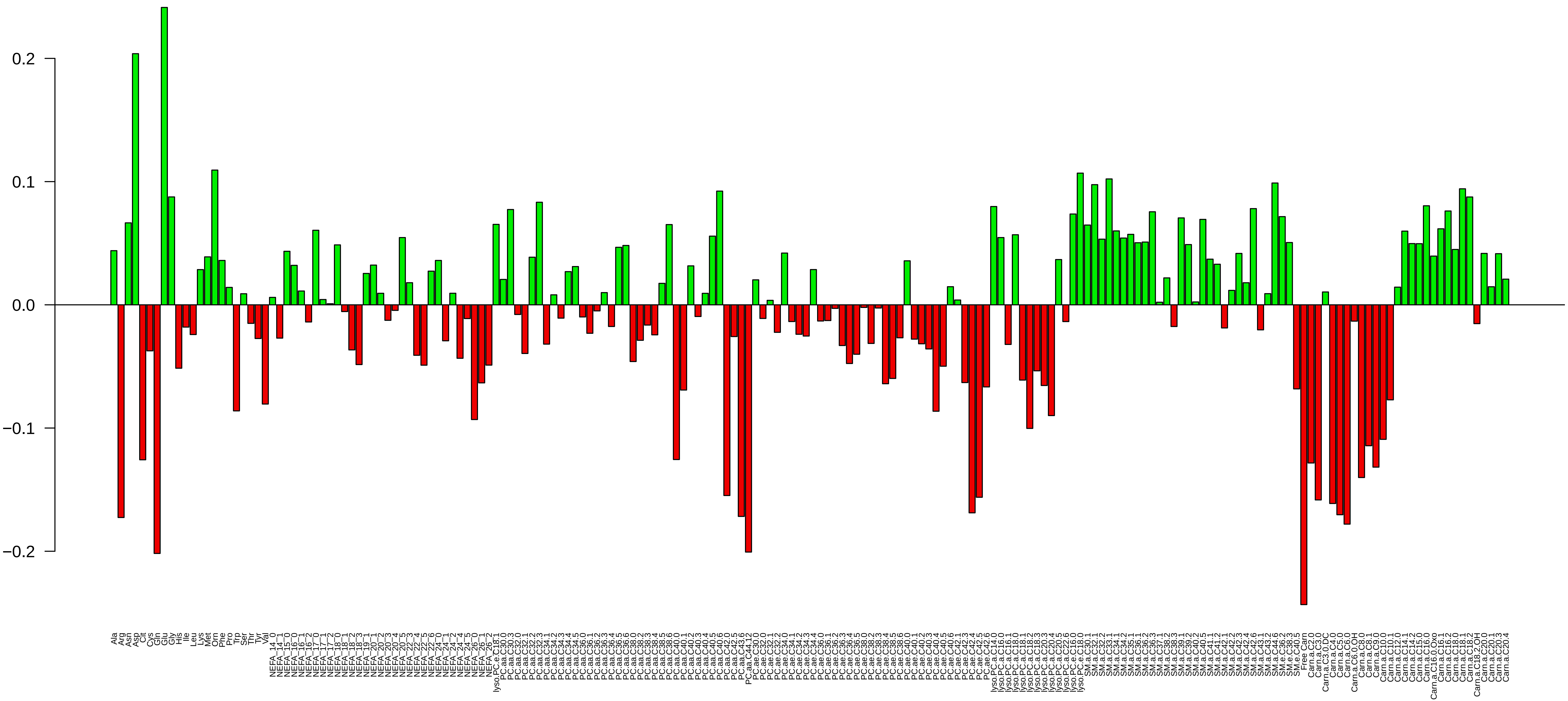

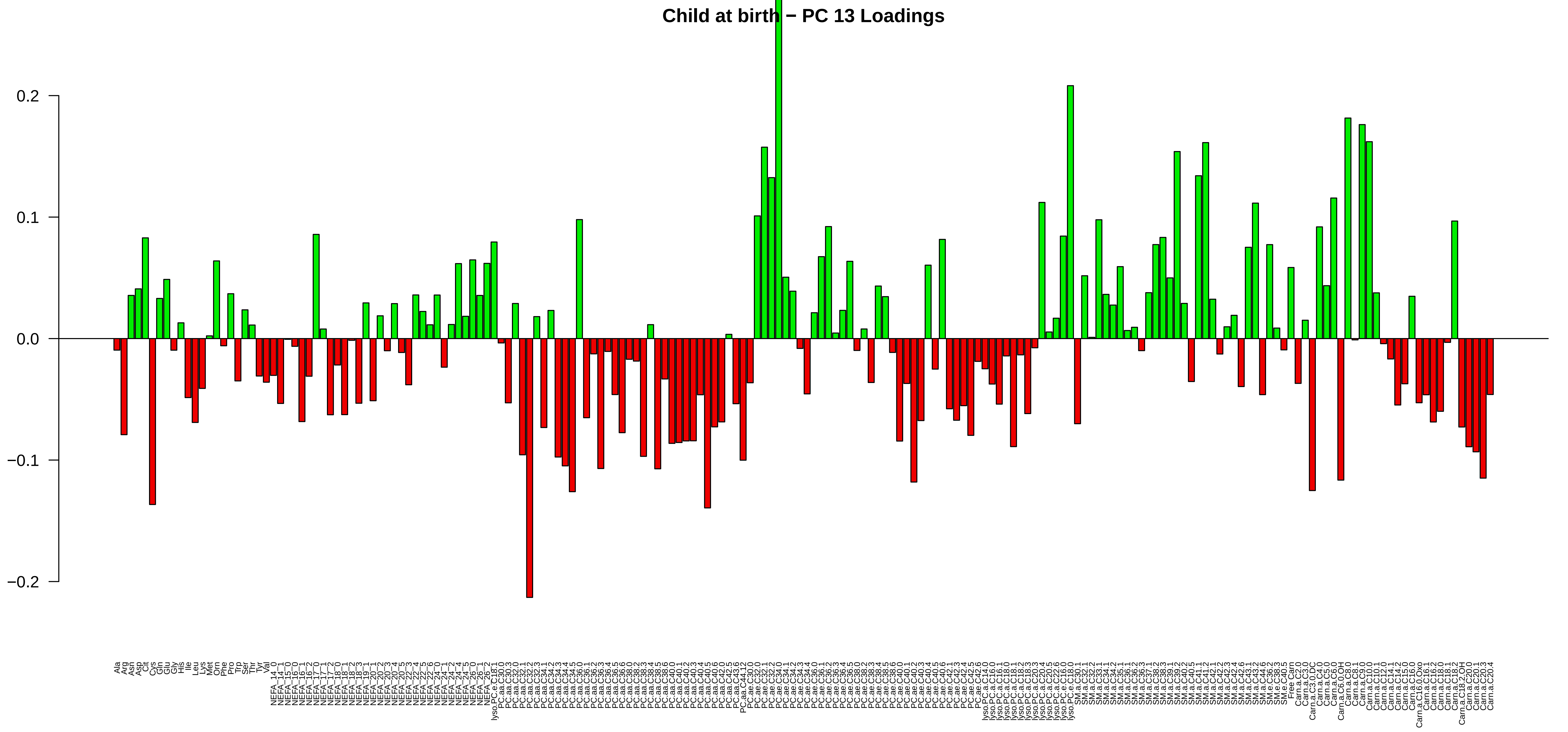
Child at birth − PC 13 Loadings
0.2
0.1
0.0
−0.1
−0.2

Child at birth − PC 14 Loadings

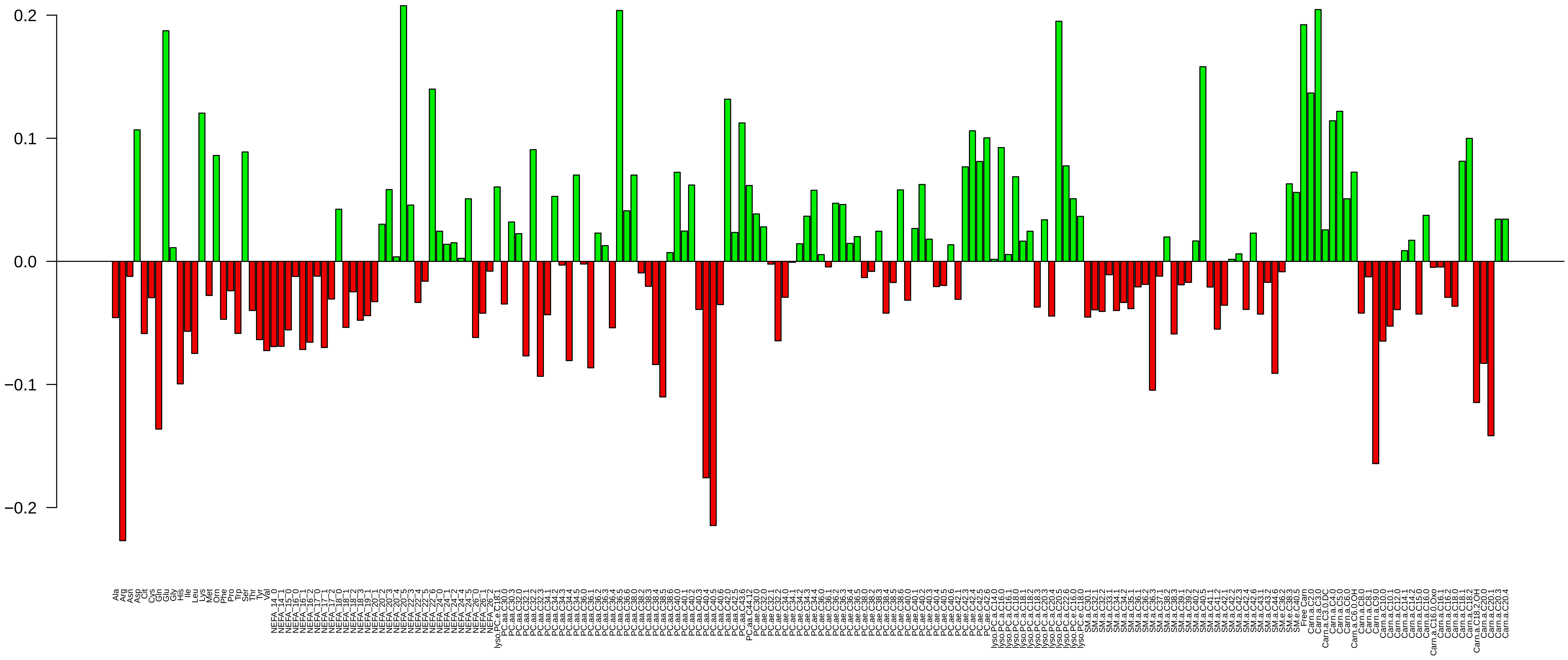

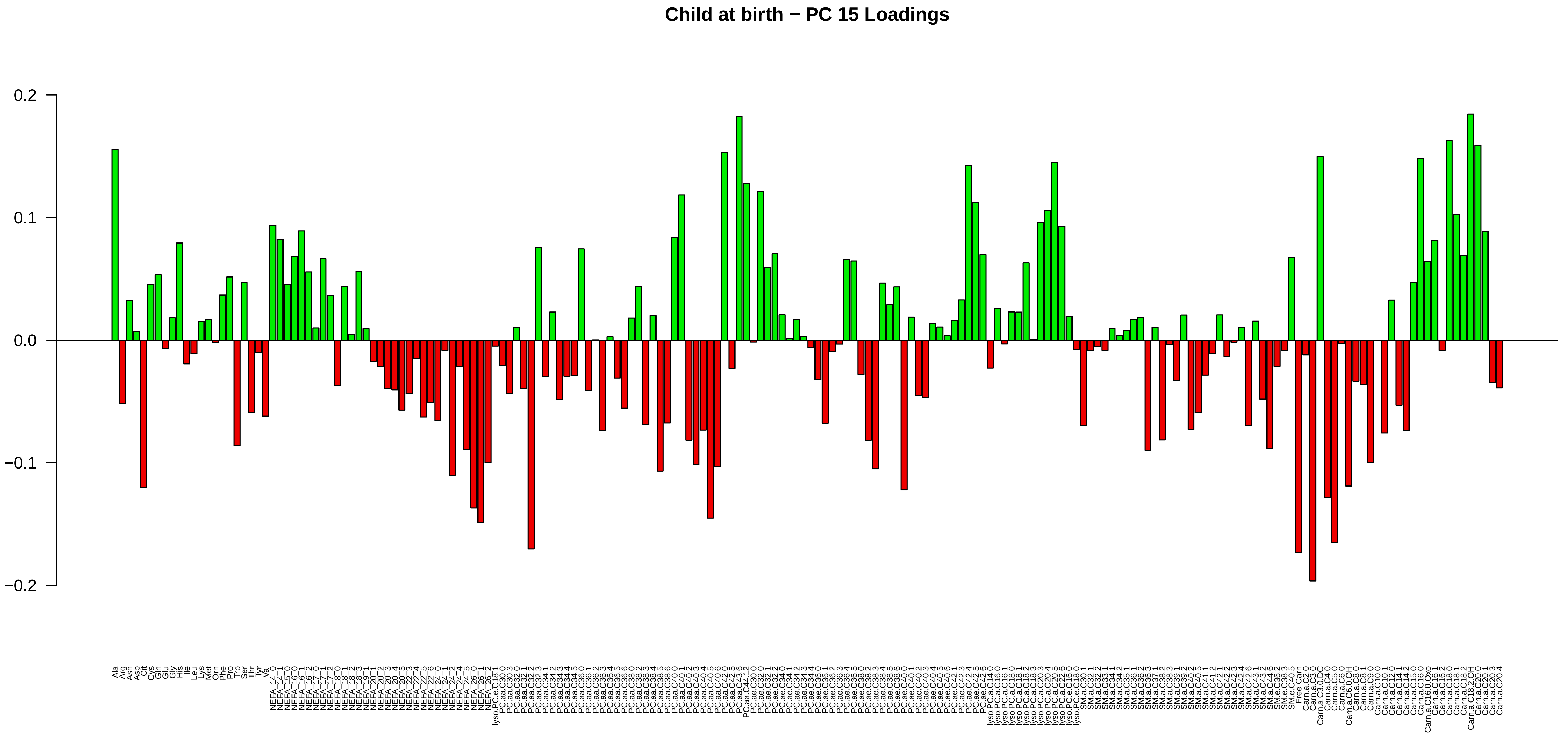

Child at birth − PC 15 Loadings
0.2
0.1
0.0
−0.1
−0.2

Child at birth − PC 16 Loadings

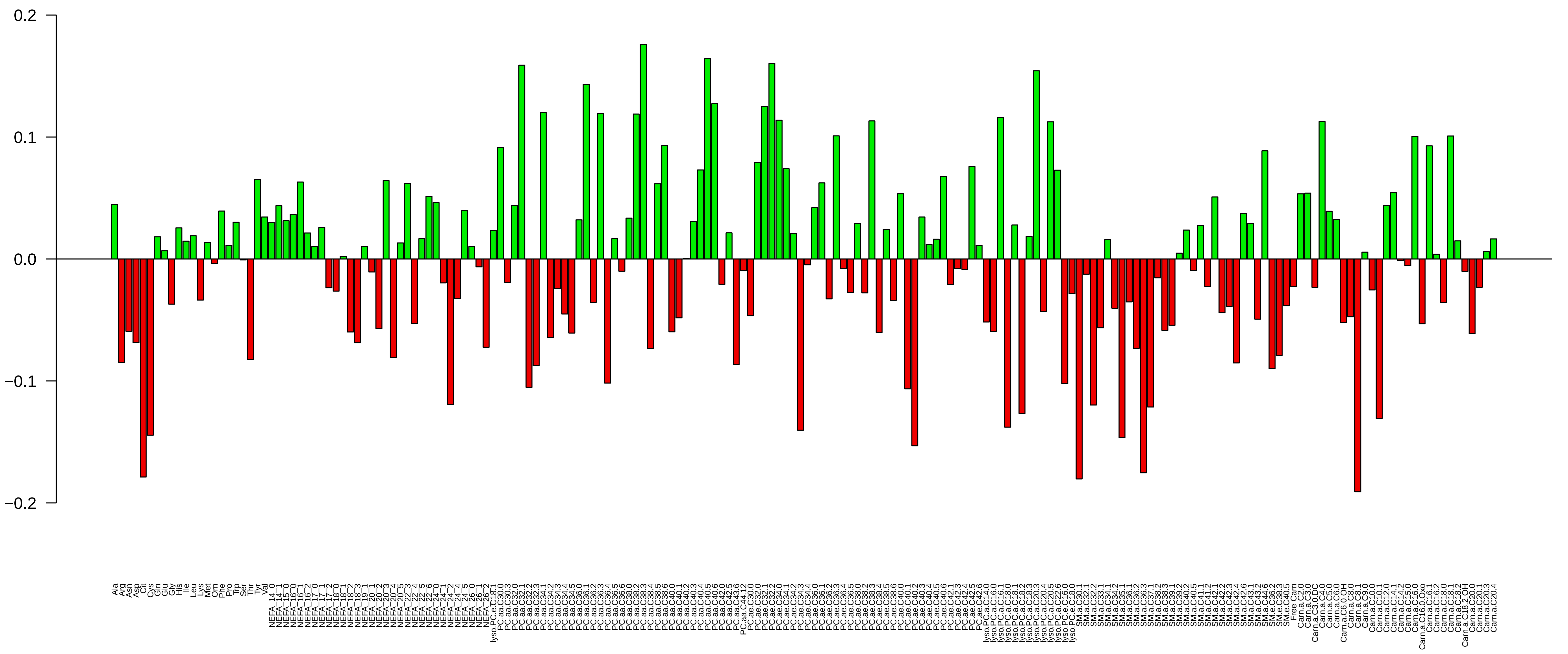

Child at birth − PC 17 Loadings

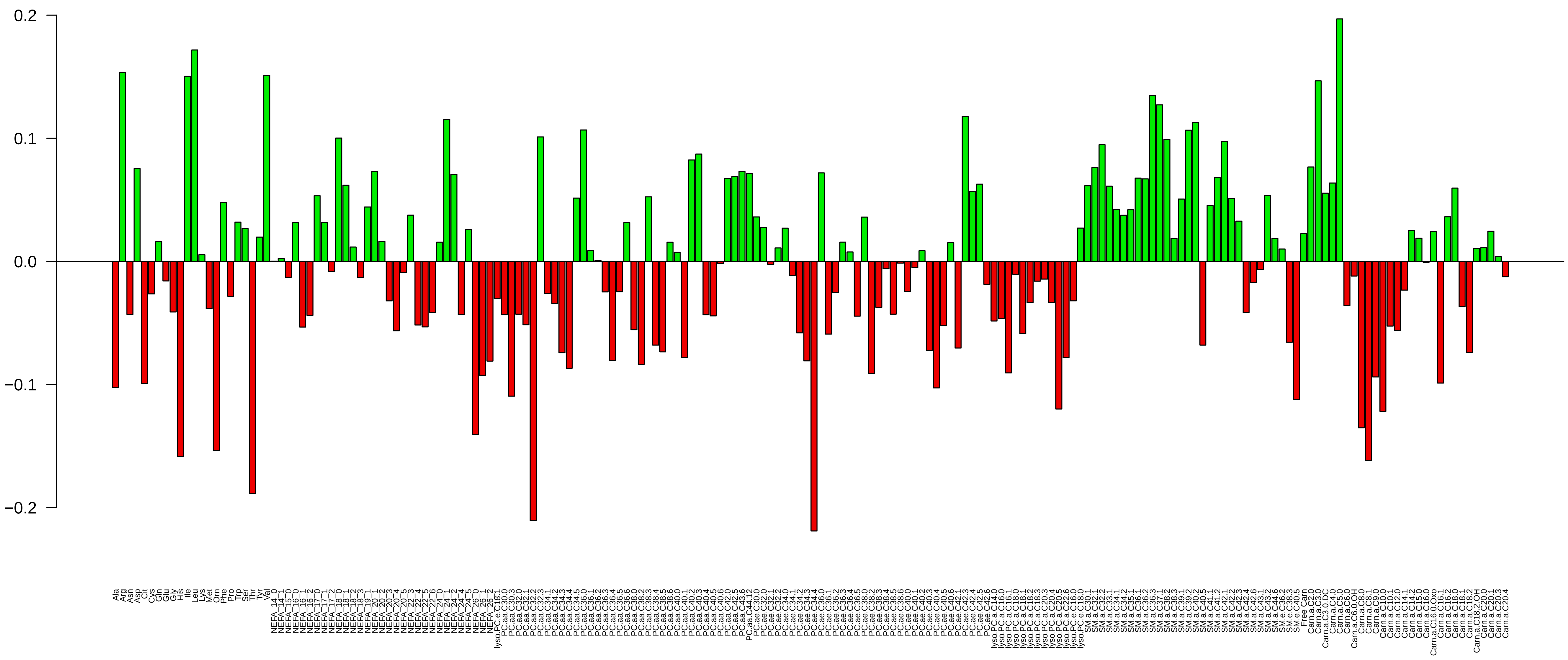

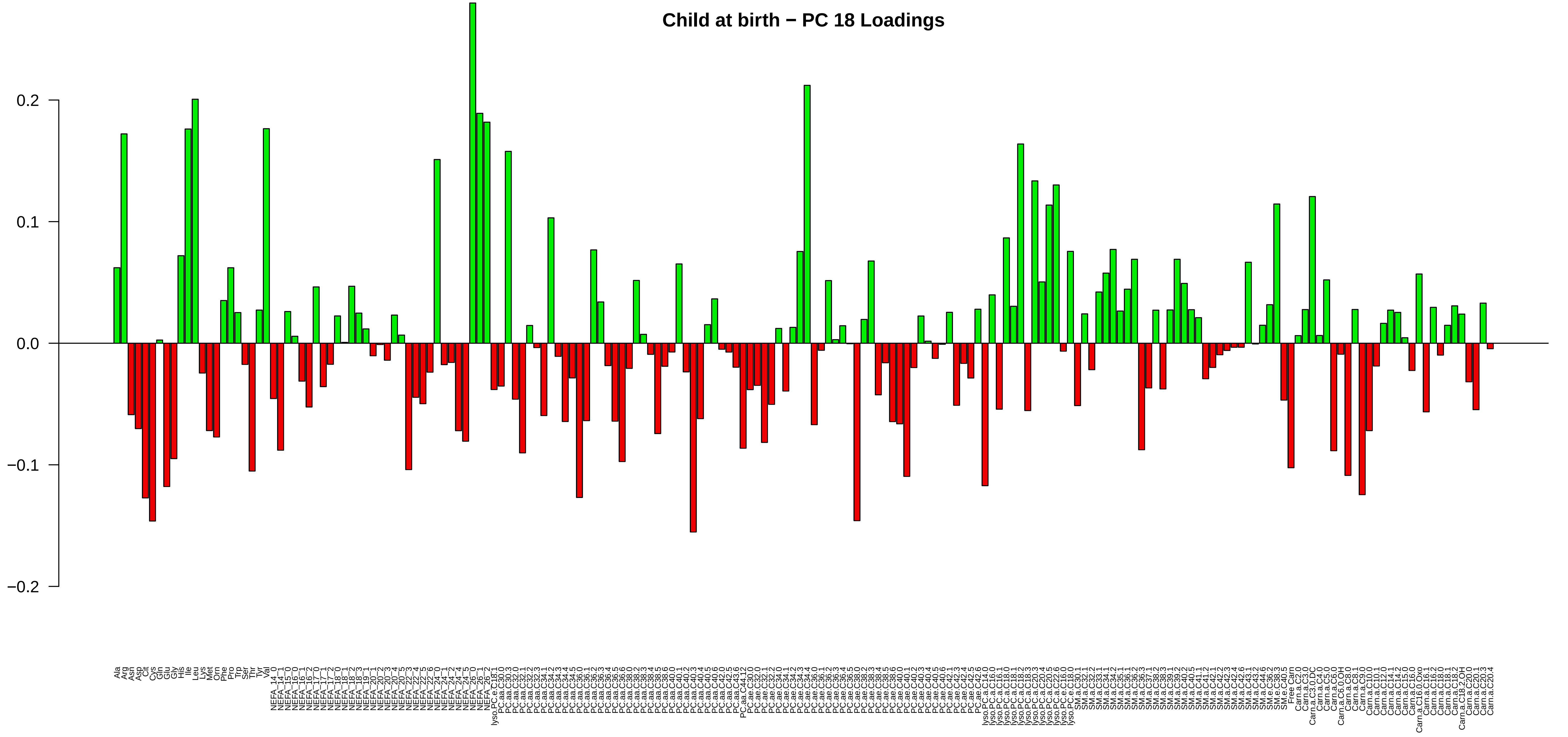
Child at birth − PC 18 Loadings
0.2
0.1
0.0
−0.1
−0.2

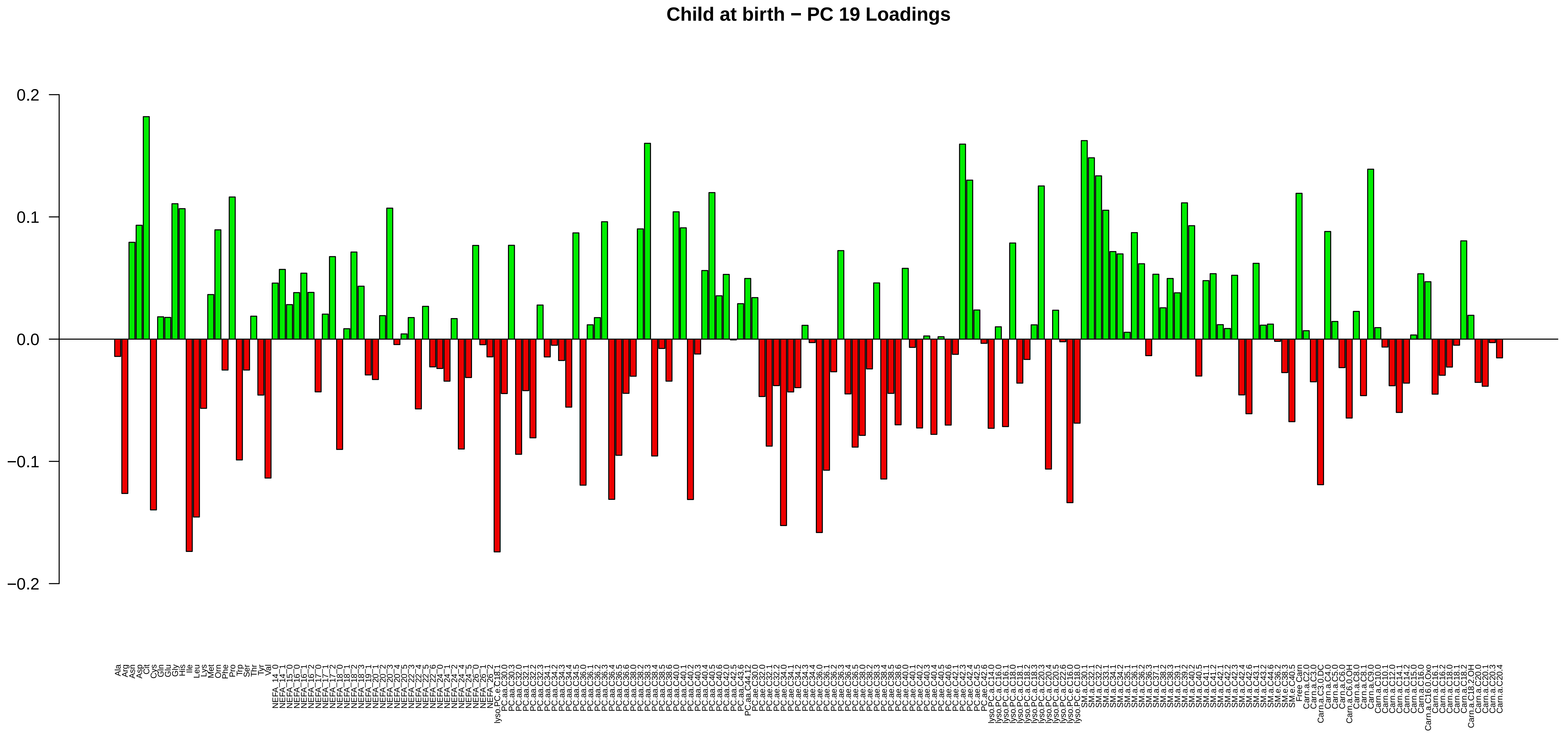
Child at birth − PC 19 Loadings
0.2
0.1
0.0
−0.1
−0.2

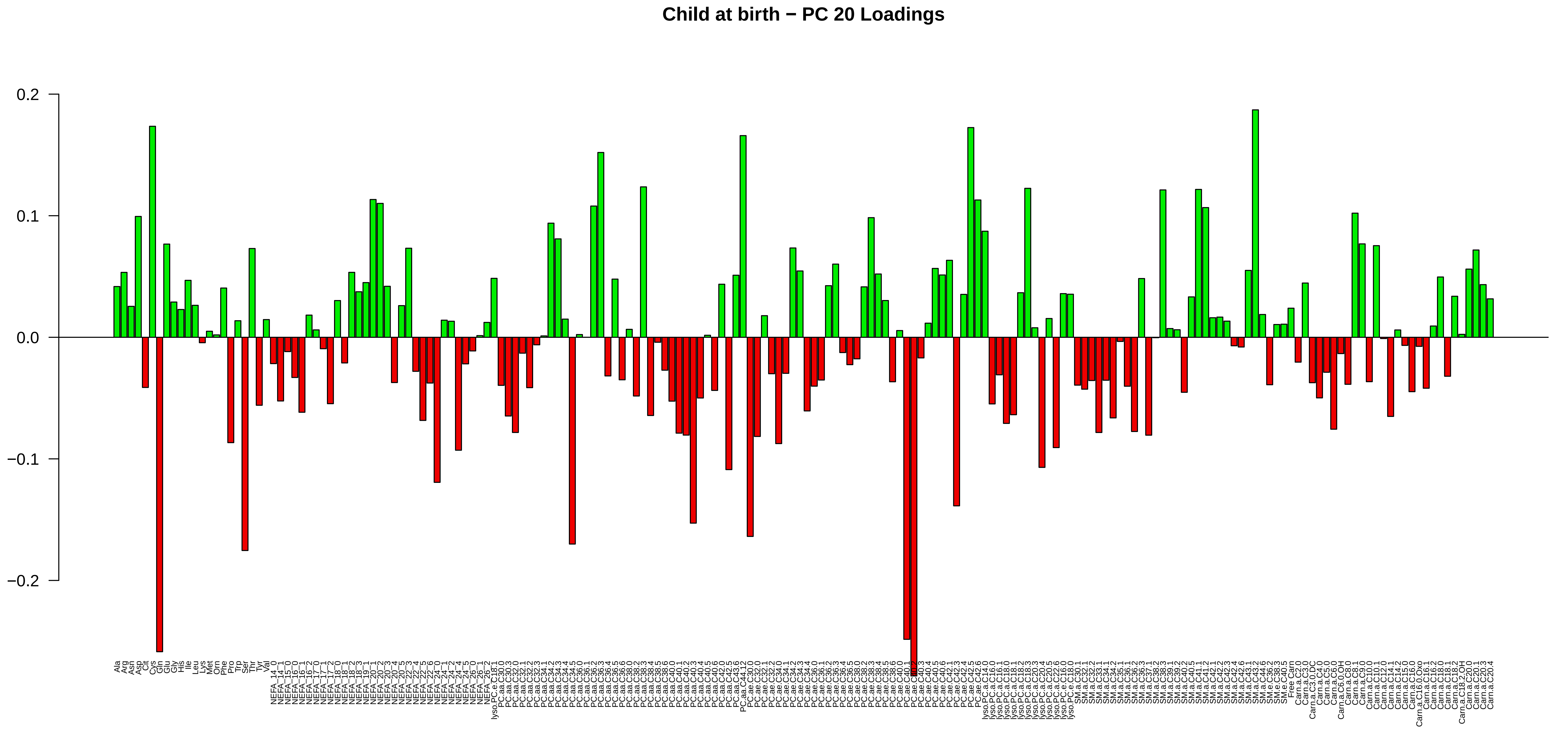
Child at birth − PC 20 Loadings
0.2
0.1
0.0
−0.1
−0.2

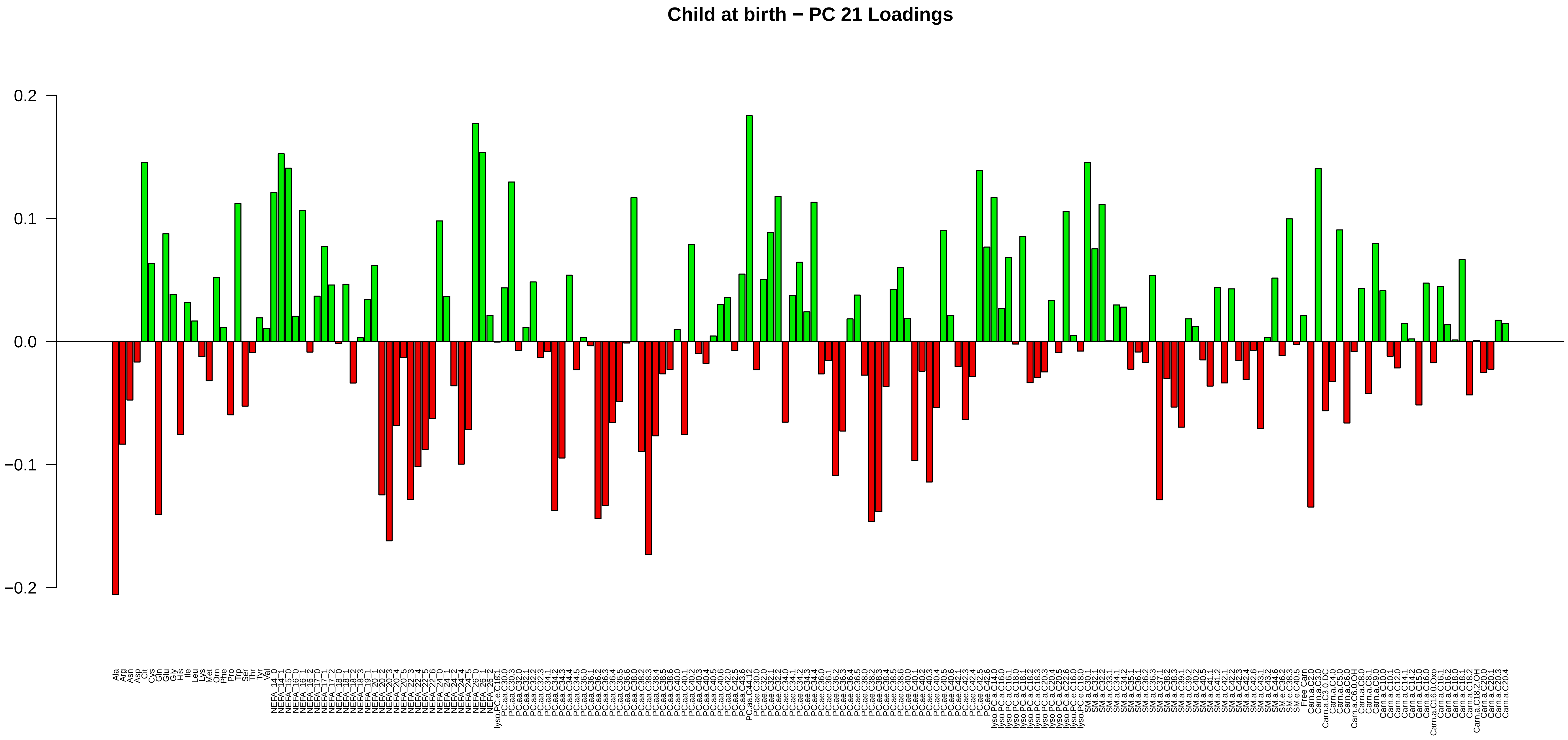
Child at birth − PC 21 Loadings
0.2
0.1
0.0
−0.1
−0.2

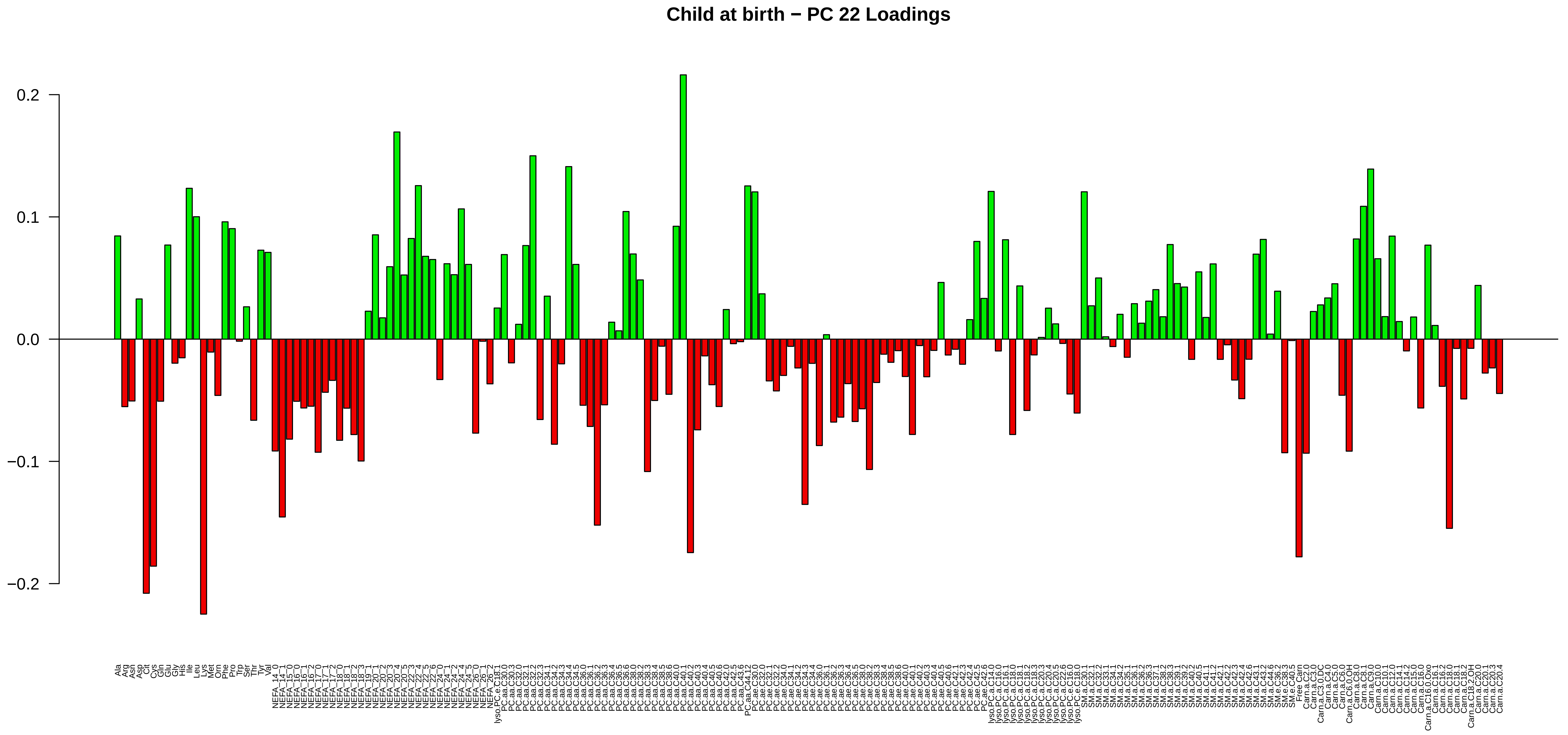
Child at birth − PC 22 Loadings
0.2
0.1
0.0
−0.1
−0.2

Child at birth − PC 23 Loadings

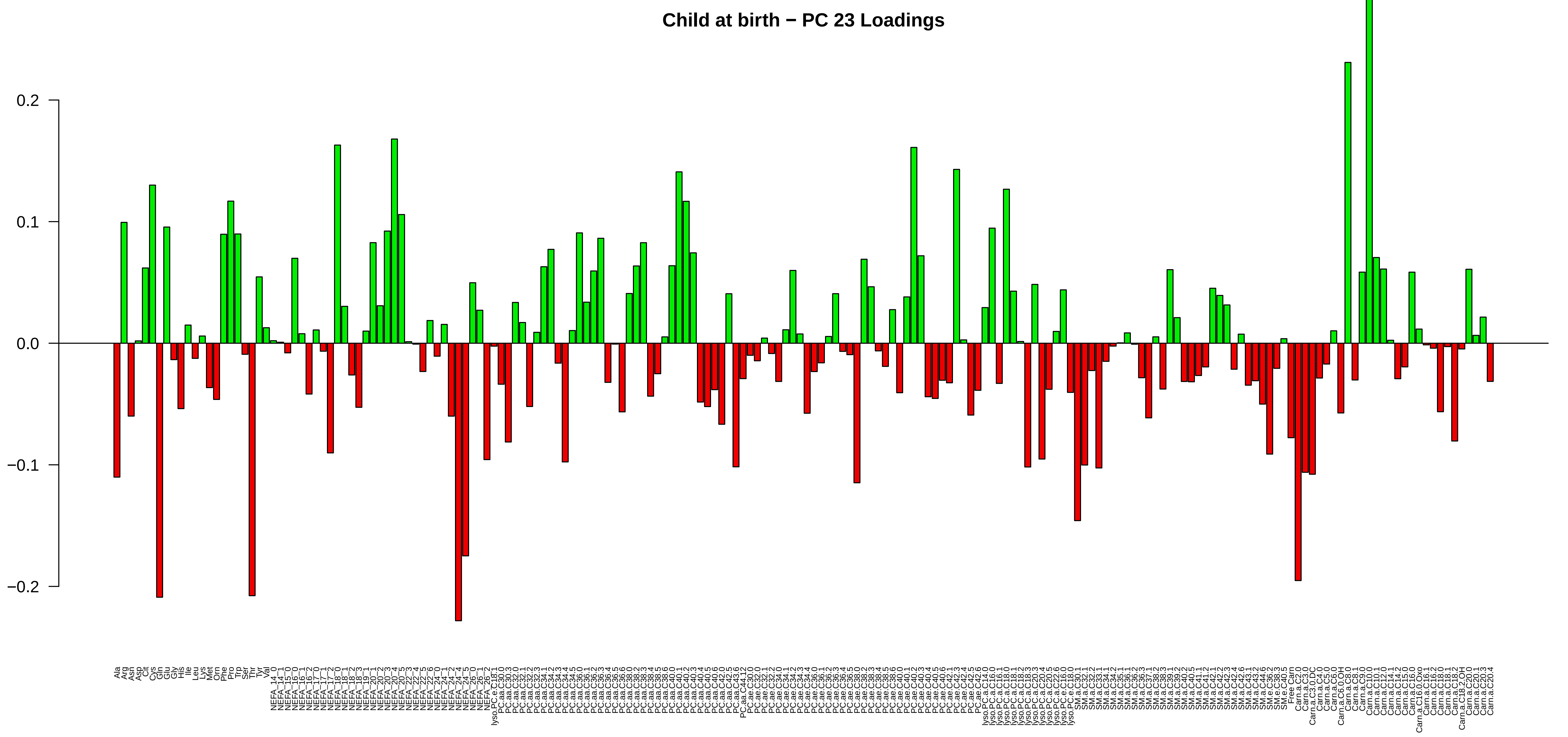

Child at birth − PC 24 Loadings

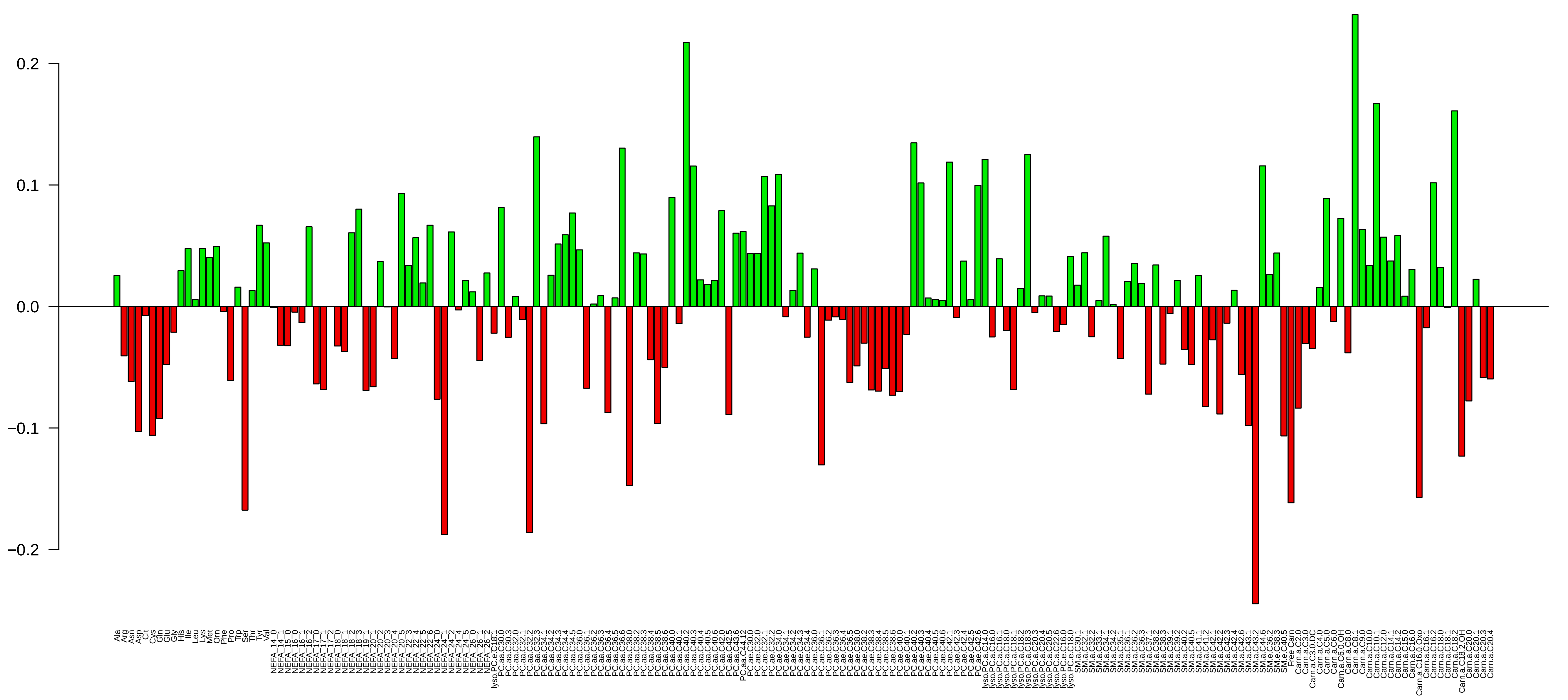

Child at birth − PC 25 Loadings

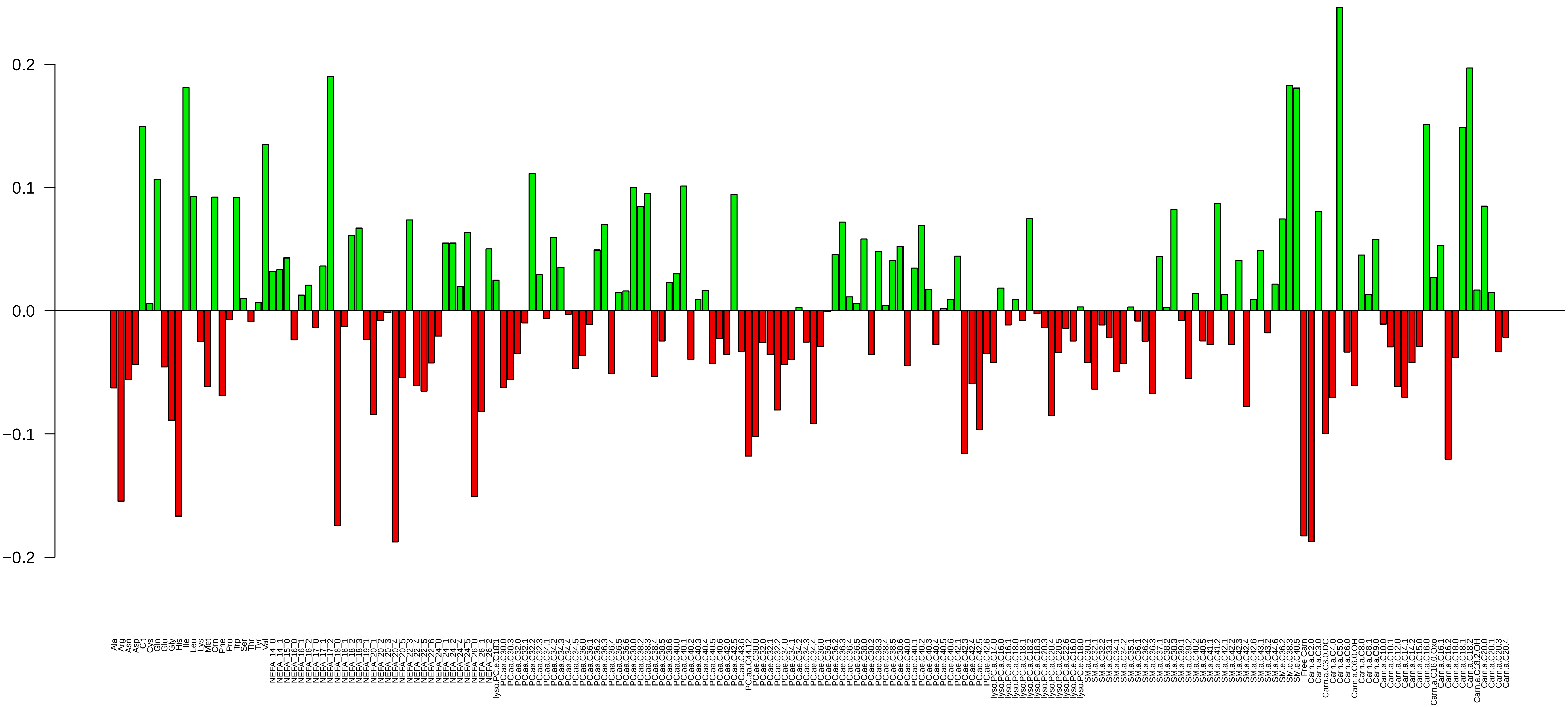

Supplement: Supplementary file 7 — Supplemental Figure S3 (PDF 106 kb) [file 11306_2020_1667_MOESM7_ESM.pdf]
