# Supplementary material for: A population-based resource for intergenerational metabolomics analyses in pregnant women and their children: the Generation R Study
Source: Metabolomics. 2020 Mar 23;16(4):43. doi: 10.1007/s11306-020-01667-1 (PMC7089886; doi:10.1007/s11306-020-01667-1)
Supplement: Supplementary file 9 — Supplemental Figure S5 (PDF 1357 kb) [file 11306_2020_1667_MOESM9_ESM.pdf]

Figure S5. Correlation networks for correlations between individual metabolite concentrations by time point

A. Mother early pregnancy

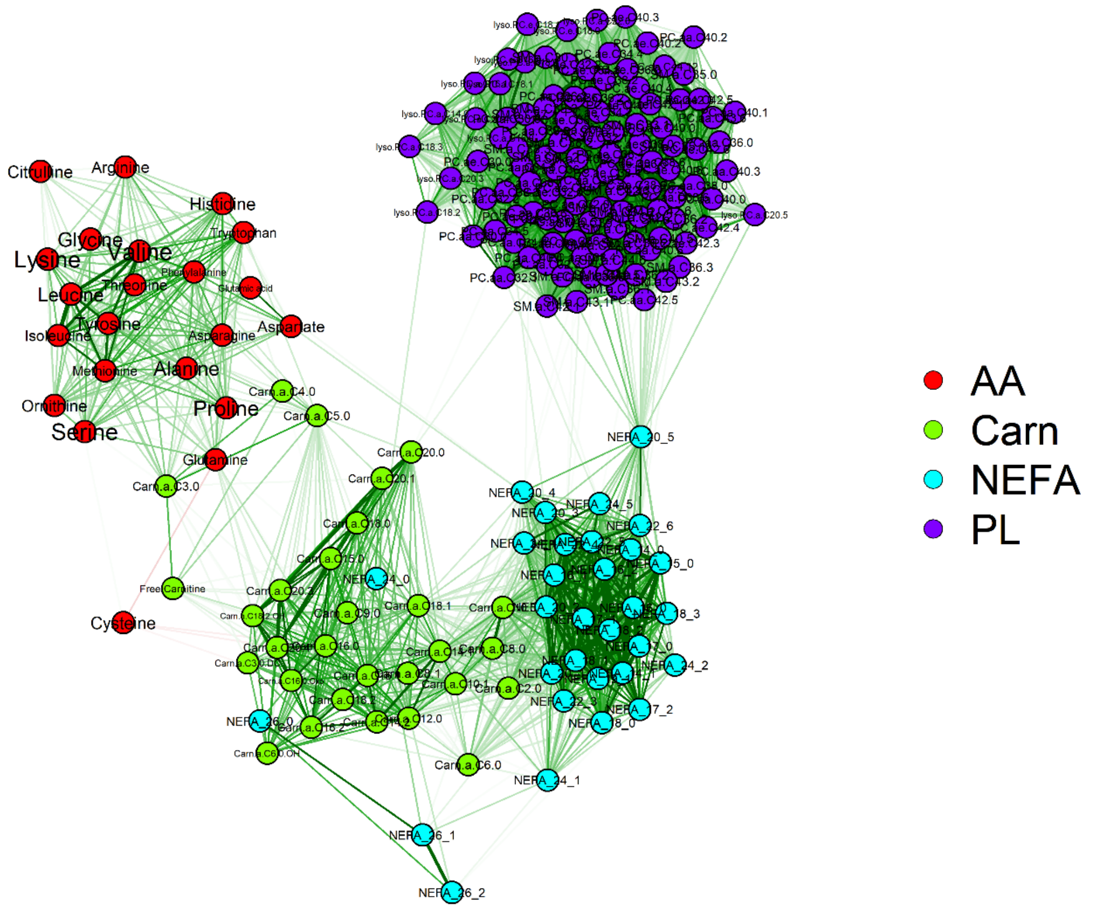

Lines represent Pearson's correlation coefficients between the individual metabolite concentrations within metabolite groups within and between metabolite groups. Green lines represent positive correlations and red lines represent negative correlations. The thickness of the lines indicates the strength of the correlations, with thinker lines for stronger correlations. Only correlation coefficients lower than -0.30 and higher than 0.30 are shown.
AA: amino acids, Carn: carnitines, NEFA: non-esterified fatty acids, PL: phospholipids.

Figure S5. Correlation networks for correlations between individual metabolite concentrations by time point (continued)

B. Child at birth

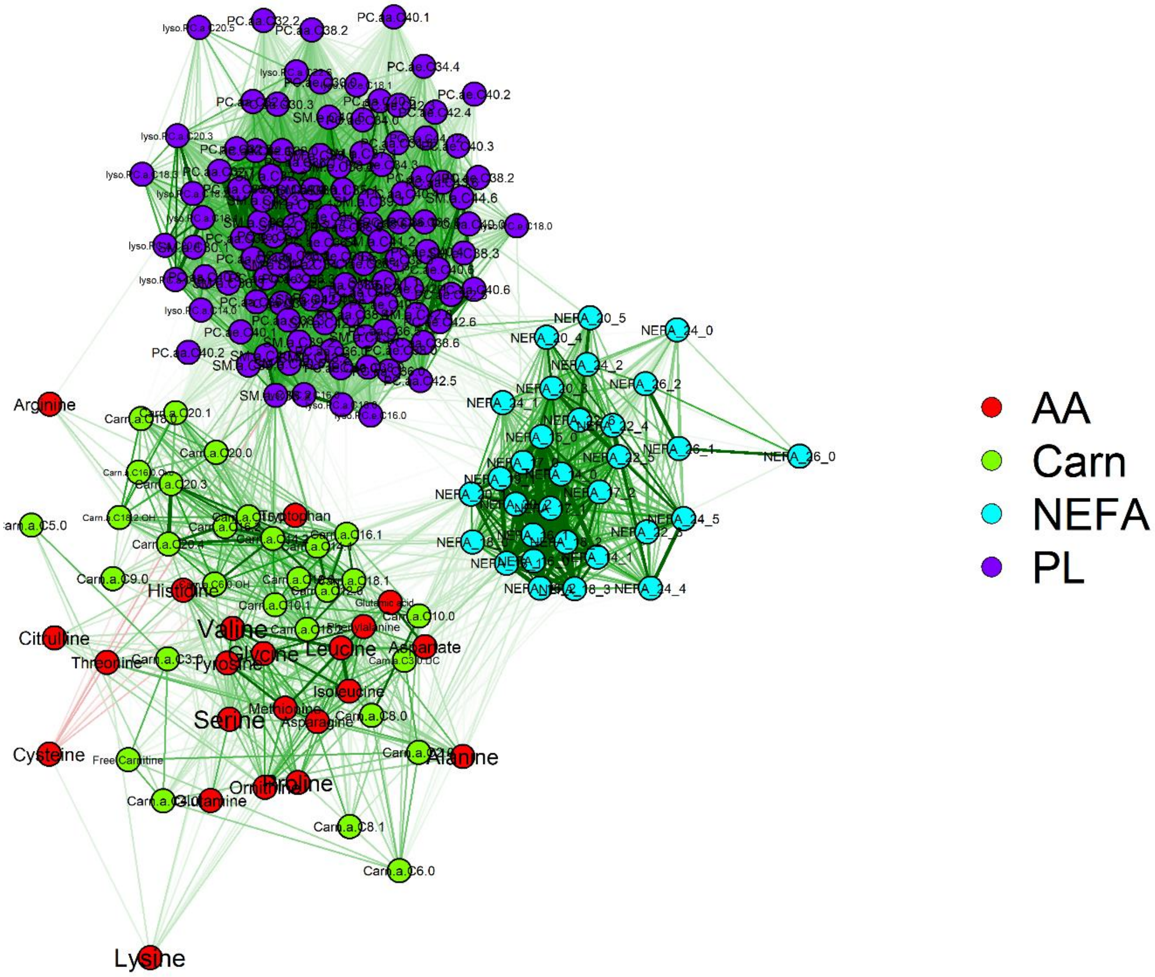

Lines represent Pearson's correlation coefficients between the individual metabolite concentrations within metabolite groups within and between metabolite groups. Green lines represent positive correlations and red lines represent negative correlations. The thickness of the lines indicates the strength of the correlations, with thinker lines for stronger correlations. Only correlation coefficients lower than -0.30 and higher than 0.30 are shown.

AA: amino acids, Carn: carnitines, NEFA: non-esterified fatty acids, PL: phospholipids.

Figure S5. Correlation networks for correlations between individual metabolite concentrations by time point (continued)

C. Child age 10 years

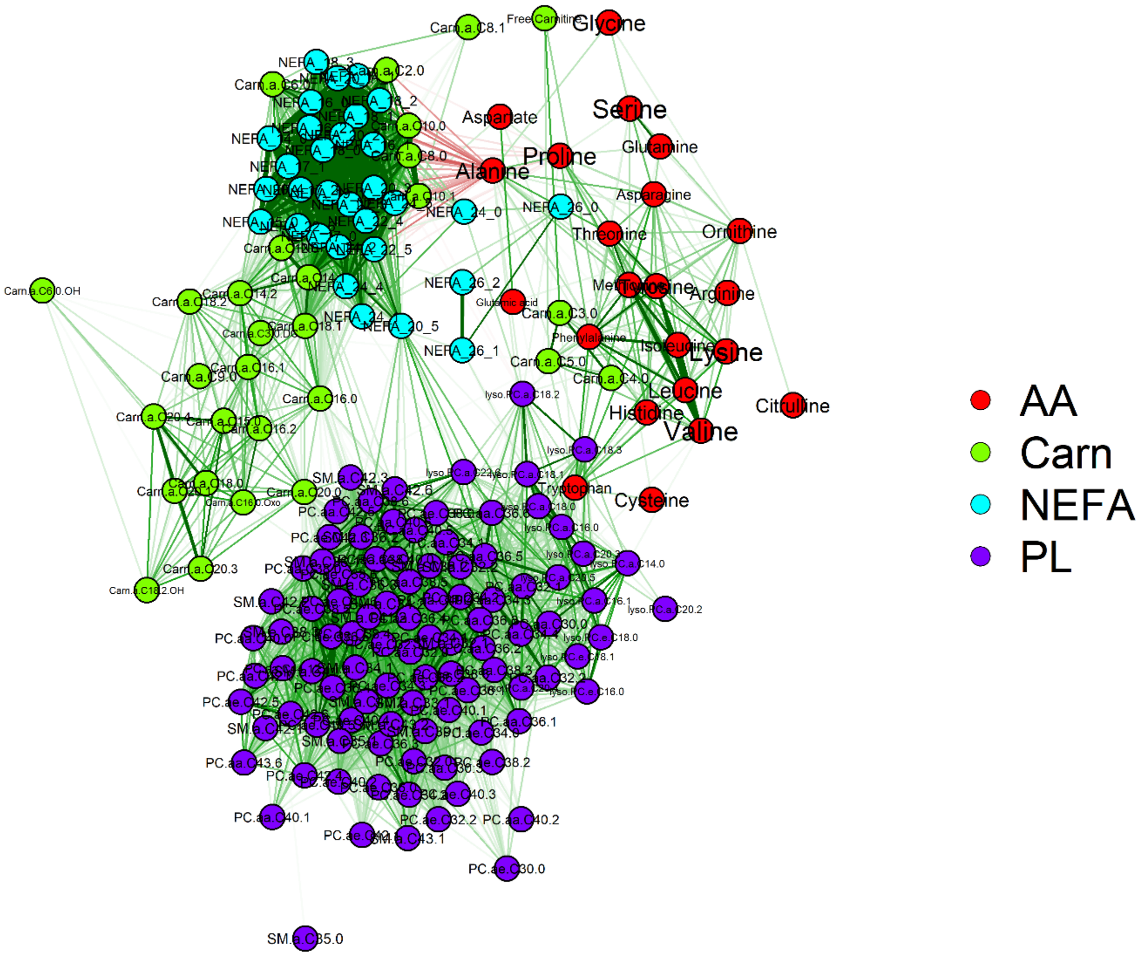

Lines represent Pearson's correlation coefficients between the individual metabolite concentrations within metabolite groups within and between metabolite groups. Green lines represent positive correlations and red lines represent negative correlations. The thickness of the lines indicates the strength of the correlations, with thinker lines for stronger correlations. Only correlation coefficients lower than -0.30 and higher than 0.30 are shown.

AA: amino acids, Carn: carnitines, NEFA: non-esterified fatty acids, PL: phospholipids.
