# Supplementary material for: A population-based resource for intergenerational metabolomics analyses in pregnant women and their children: the Generation R Study
Source: Metabolomics. 2020 Mar 23;16(4):43. doi: 10.1007/s11306-020-01667-1 (PMC7089886; doi:10.1007/s11306-020-01667-1)
Supplement: Supplementary file 10 — Supplemental Figure S6 (PDF 665 kb) [file 11306_2020_1667_MOESM10_ESM.pdf]

Figure S6. Circos plots of correlations between individual metabolite concentrations, stratified by child's sex

A. r < -0.15 and > 0.15

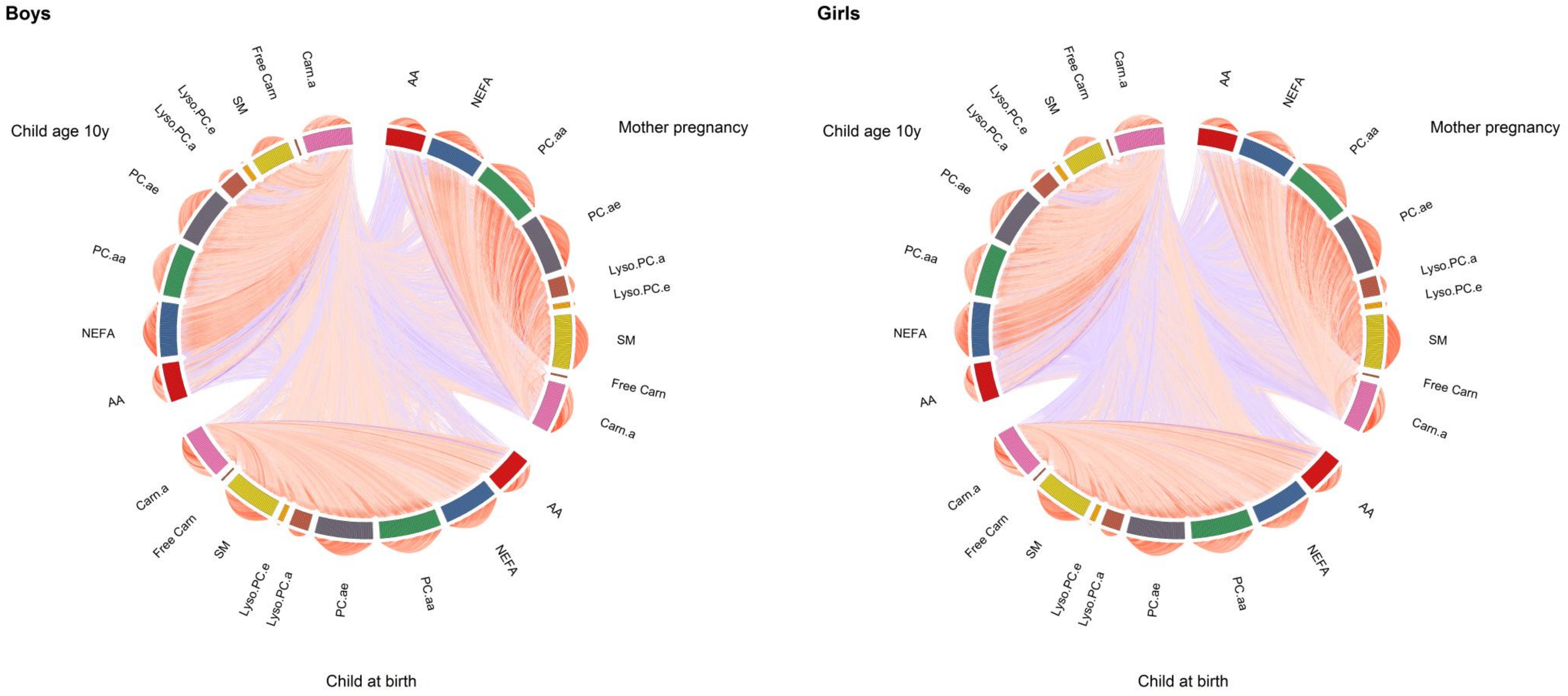

Lines represent Pearson's correlation coefficients between the individual metabolite concentrations within metabolite groups (outer circle), between metabolite groups (inner circle) and between time points (lines going through the middle of the circle), stratified by child's sex. Red lines represent positive correlations and blue lines represent negative correlations. The brightness of the lines indicates the strength of the correlations, with brighter colors for stronger correlations. Figure 2A shows only correlation coefficients lower than -0.15 and higher than 0.15 and Figure 2B shows only correlation coefficients lower than -0.30 and higher than 0.30.
AA: amino acids, NEFA: non-esterified fatty acids, PC.aa: diacyl-phosphatidylcholines, PC.ae: acyl-alkyl-phosphatidylcholines, Lyso.PC.a: acyl-lysophosphatidylcholines, Lyso.PC.e: alkyl-lysophosphatidylcholines, SM: sphingomyelines, Free Carn: free carnitine, Carn.a: acyl-carnitines.

Figure S6. Circos plots of correlations between individual metabolite concentrations, stratified by child's sex (continued)

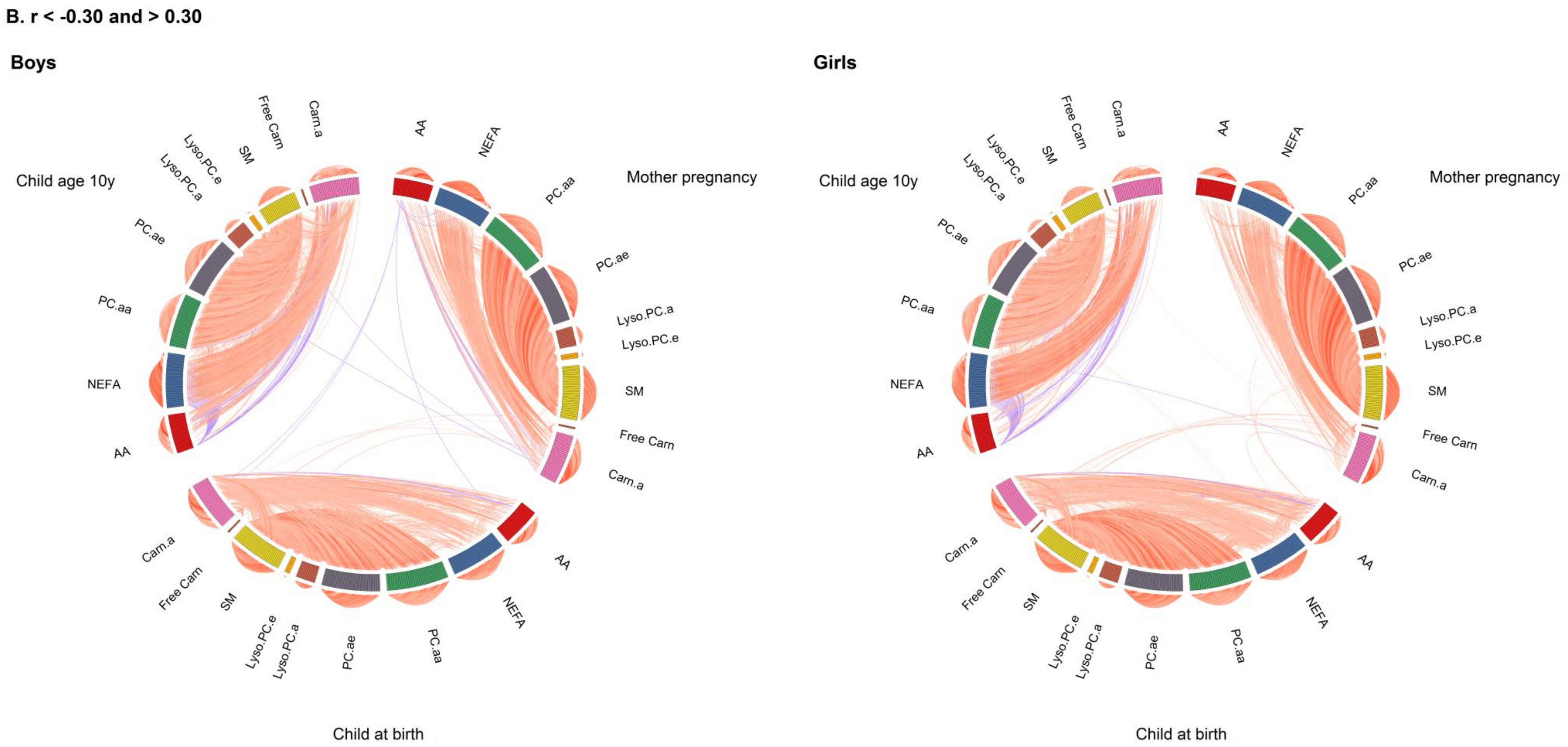

Lines represent Pearson's correlation coefficients between the individual metabolite concentrations within metabolite groups (outer circle), between metabolite groups (inner circle) and between time points (lines going through the middle of the circle), stratified by child's sex. Red lines represent positive correlations and blue lines represent negative correlations. The brightness of the lines indicates the strength of the correlations, with brighter colors for stronger correlations. Figure 2A shows only correlation coefficients lower than -0.15 and higher than 0.15 and Figure 2B shows only correlation coefficients lower than -0.30 and higher than 0.30.

AA: amino acids, NEFA: non-esterified fatty acids, PC.aa: diacyl-phosphatidylcholines, PC.ae: acyl-alkyl-phosphatidylcholines, Lyso.PC.a: acyl-lysophosphatidylcholines, Lyso.PC.e: alkyl-lysophosphatidylcholines, SM: sphingomyelines, Free Carn: free carnitine, Carn.a: acyl-carnitine.
