# Supplementary material for: A population-based resource for intergenerational metabolomics analyses in pregnant women and their children: the Generation R Study
Source: Metabolomics. 2020 Mar 23;16(4):43. doi: 10.1007/s11306-020-01667-1 (PMC7089886; doi:10.1007/s11306-020-01667-1)
Supplement: Supplementary file 11 — Supplemental Figure S7 (PDF 1942 kb) [file 11306_2020_1667_MOESM11_ESM.pdf]

Figure S7. Heatmap of median absolute correlation of individual metabolites within and between metabolite groups by time point, stratified by child's sex

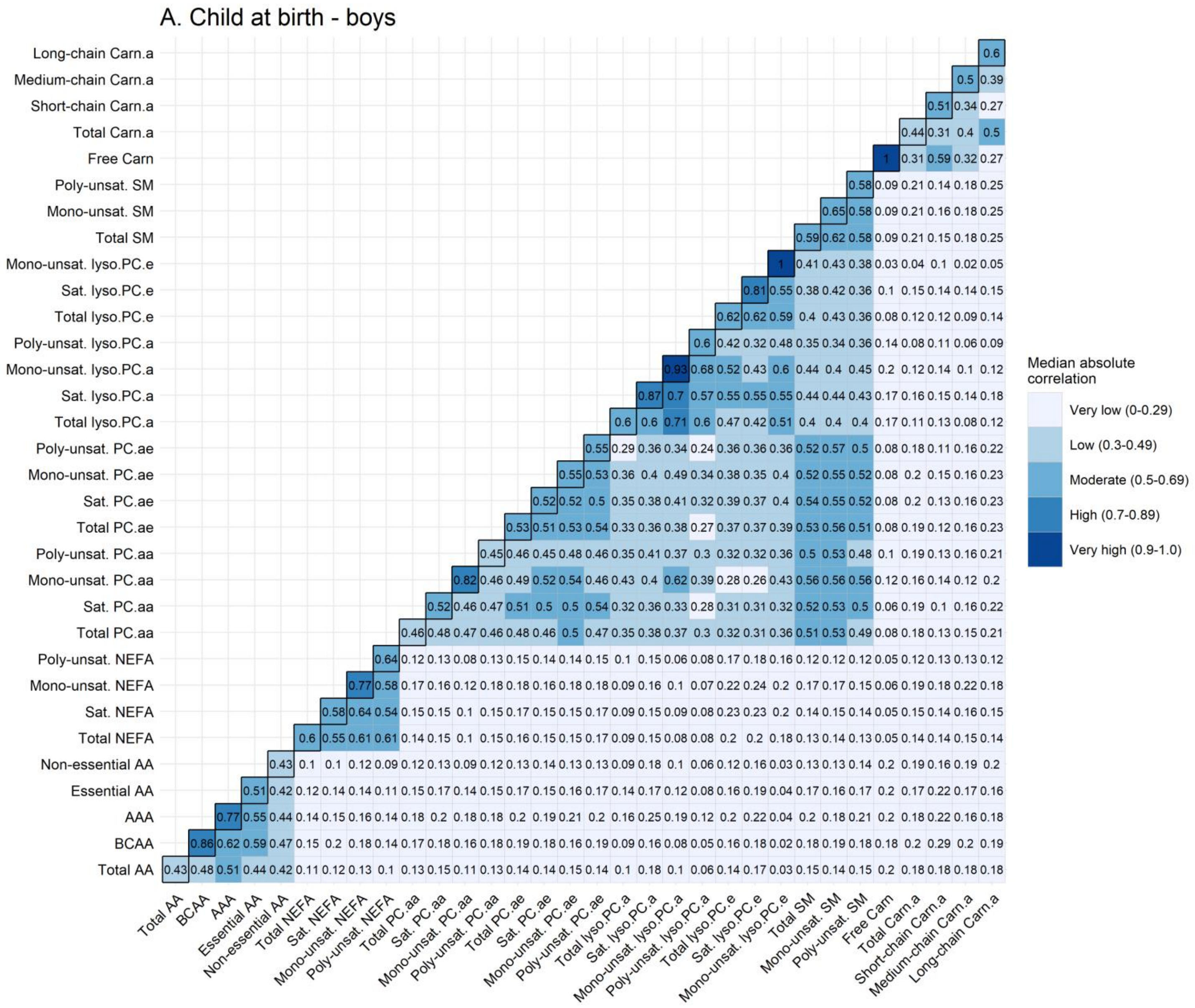

Values represent median absolute correlation coefficients of individual metabolite concentrations within metabolite groups (diagonal) and between metabolite groups (off-diagonal), stratified by child's sex. Mono-unsaturated lyso.PC.e, saturated SM and Free Carn include 1 metabolite, resulting in a correlation coefficient of 1 for within-group correlations. For child at birth, no data on saturated SM is available.

AA: amino acids, BCAA: branched-chain amino acids, AAA: aromatic amino acids, NEFA: non-esterified fatty acids, PC.aa: diacyl-phosphatidylcholines, PC.ae: acyl-alkyl-phosphatidylcholines, Lyso.PC.a: acyl-lysophosphatidylcholines, Lyso.PC.e: alkyl-lysophosphatidylcholines, SM: sphingomyelines, Free Carn: free carnitine, Carn.a: acyl-carnitines, Sat.: Saturated, Unsat.: Unsaturated.

Figure S7. Heatmap of median absolute correlation of individual metabolites within and between metabolite groups by time point, stratified by child's sex (continued)

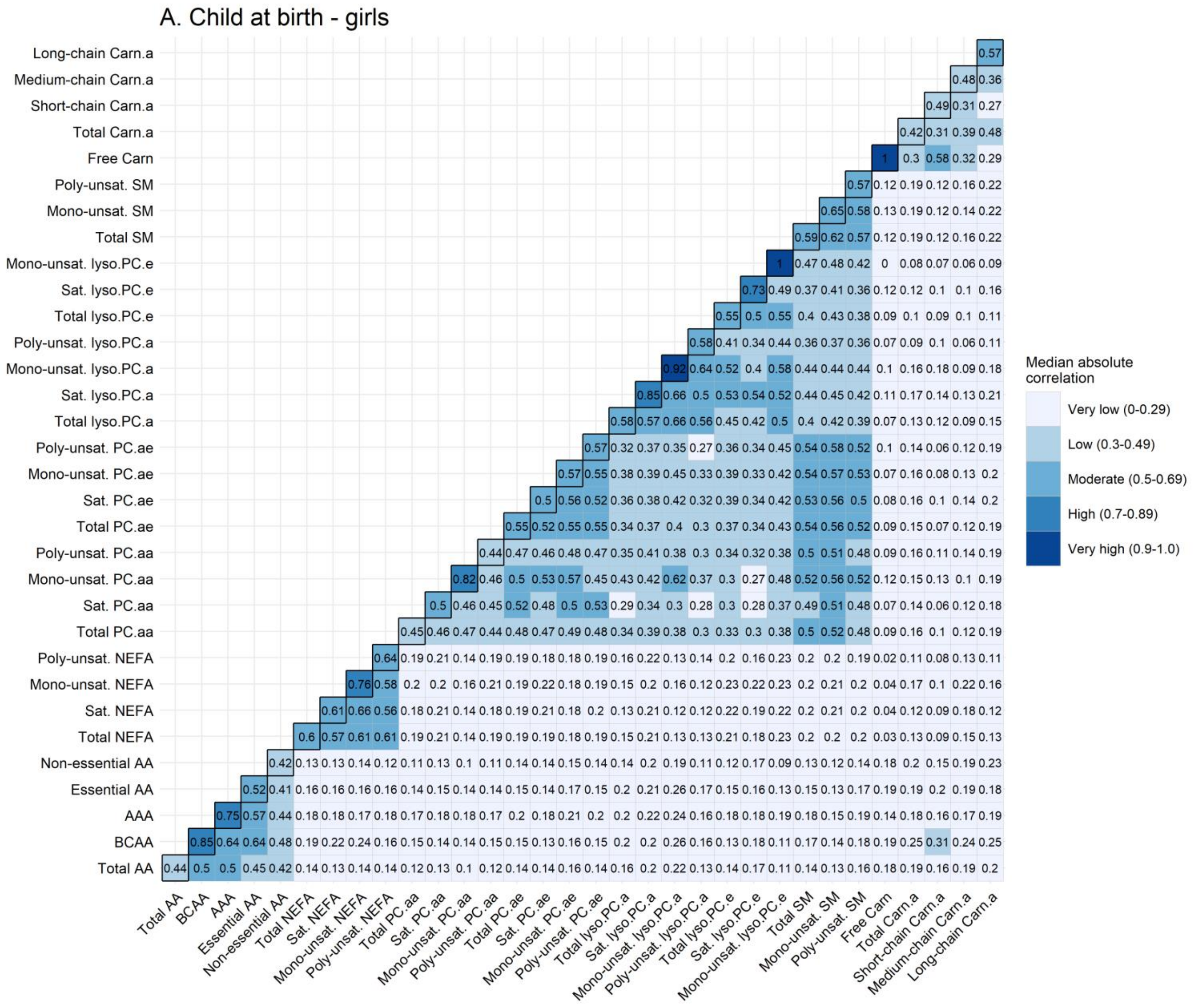

Values represent median absolute correlation coefficients of individual metabolite concentrations within metabolite groups (diagonal) and between metabolite groups (off-diagonal), stratified by child's sex. Mono-unsaturated lyso.PC.e, saturated SM and Free Carn include 1 metabolite, resulting in a correlation coefficient of 1 for within-group correlations. For child at birth, no data on saturated SM is available.

AA: amino acids, BCAA: branched-chain amino acids, AAA: aromatic amino acids, NEFA: non-esterified fatty acids, PC.aa: diacyl-phosphatidylcholines, PC.ae: acyl-alkyl-phosphatidylcholines, Lyso.PC.a: acyl-lysophosphatidylcholines, Lyso.PC.e: alkyl-lysophosphatidylcholines, SM: sphingomyelines, Free Carn: free carnitine, Carn.a: acyl-carnitines, Sat.: Saturated, Unsat.: Unsaturated.

Figure S7. Heatmap of median absolute correlation of individual metabolites within and between metabolite groups by time point, stratified by child's sex (continued)

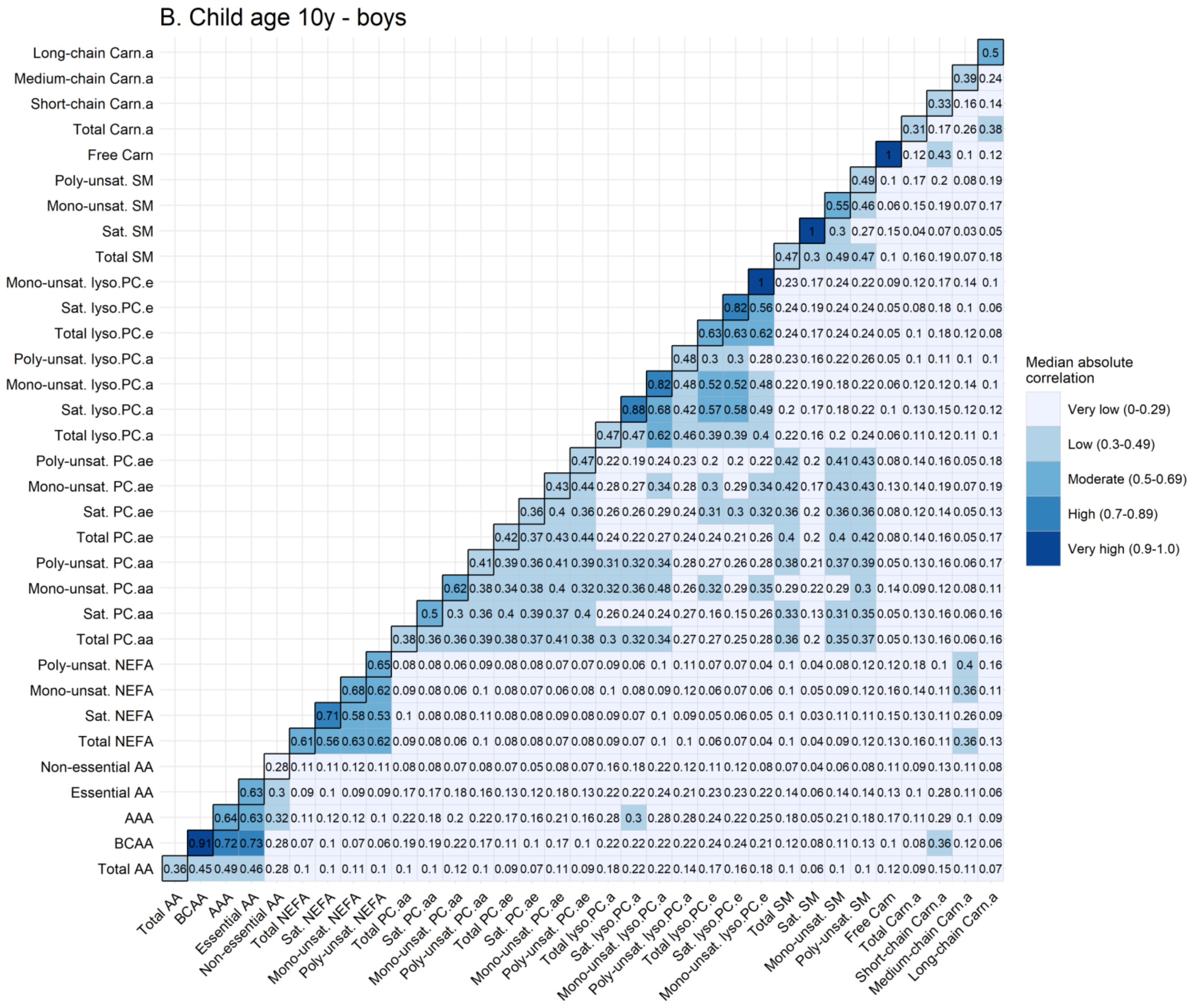

Values represent median absolute correlation coefficients of individual metabolite concentrations within metabolite groups (diagonal) and between metabolite groups (off-diagonal), stratified by child's sex. Mono-unsaturated lyso.PC.e, saturated SM and Free Carn include 1 metabolite, resulting in a correlation coefficient of 1 for within-group correlations. For child at birth, no data on saturated SM is available.

AA: amino acids, BCAA: branched-chain amino acids, AAA: aromatic amino acids, NEFA: non-esterified fatty acids, PC.aa: diacyl-phosphatidylcholines, PC.ae: acyl-alkyl-phosphatidylcholines, Lyso.PC.a: acyl-lysophosphatidylcholines, Lyso.PC.e: alkyl-lysophosphatidylcholines, SM: sphingomyelines, Free Carn: free carnitine, Carn.a: acyl-carnitines, Sat.: Saturated, Unsat.: Unsaturated.

Figure S7. Heatmap of median absolute correlation of individual metabolites within and between metabolite groups by time point, stratified by child's sex (continued)

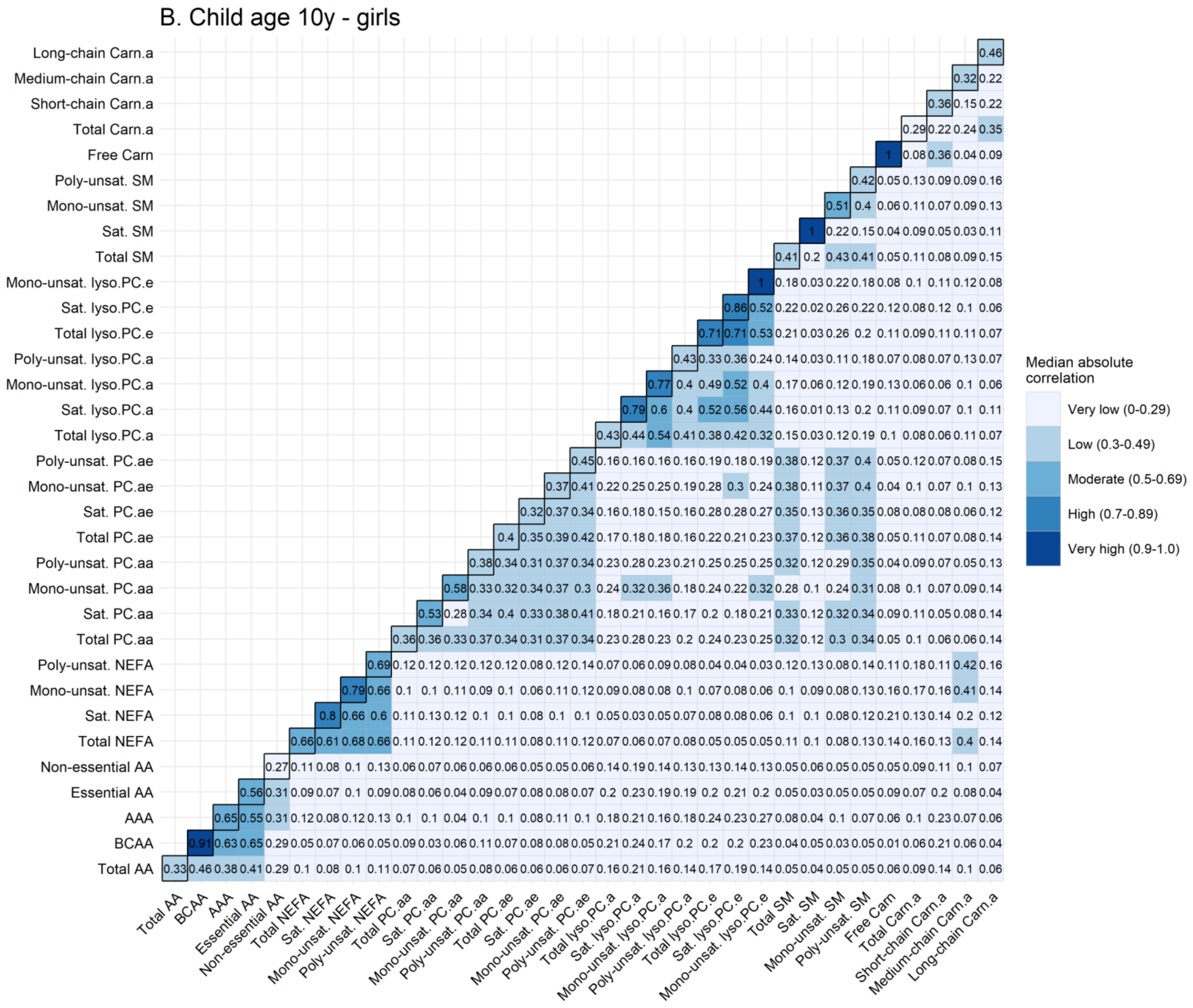

Values represent median absolute correlation coefficients of individual metabolite concentrations within metabolite groups (diagonal) and between metabolite groups (off-diagonal), stratified by child's sex. Mono-unsaturated lyso.PC.e, saturated SM and Free Carn include 1 metabolite, resulting in a correlation coefficient of 1 for within-group correlations. For child at birth, no data on saturated SM is available.

AA: amino acids, BCAA: branched-chain amino acids, AAA: aromatic amino acids, NEFA: non-esterified fatty acids, PC.aa: diacyl-phosphatidylcholines, PC.ae: acyl-alkyl-phosphatidylcholines, Lyso.PC.a: acyl-lysophosphatidylcholines, Lyso.PC.e: alkyl-lysophosphatidylcholines, SM: sphingomyelines, Free Carn: free carnitine, Carn.a: acyl-carnitines, Sat.: Saturated, Unsat.: Unsaturated.
